# MRI based radiomics enhances prediction of neurodevelopmental outcome in very preterm neonates

**DOI:** 10.1038/s41598-022-16066-w

**Published:** 2022-07-13

**Authors:** Matthias W. Wagner, Delvin So, Ting Guo, Lauren Erdman, Min Sheng, S. Ufkes, Ruth E. Grunau, Anne Synnes, Helen M. Branson, Vann Chau, Manohar M. Shroff, Birgit B. Ertl-Wagner, Steven P. Miller

**Affiliations:** 1 Department of Diagnostic Imaging, Division of Neuroradiology, The Hospital for Sick Children, 555 University Ave, Toronto, ON M5G 1X8 Canada; 2 Department of Medical Imaging, University of Toronto, Toronto, Canada; 3 Center for Computational Medicine, The Hospital for Sick Children, University of Toronto, Toronto, Canada; 4 Department of Neurology, The Hospital for Sick Children, University of Toronto, Toronto, Canada; 5 Department of Pediatrics, University of British Columbia and BC Women’s Hospital and Health Centre, Vancouver, Canada

**Keywords:** Medical research, Biomarkers, Diseases of the nervous system

## Abstract

To predict adverse neurodevelopmental outcome of very preterm neonates. A total of 166 preterm neonates born between 24–32 weeks’ gestation underwent brain MRI early in life. Radiomics features were extracted from T1- and T2- weighted images. Motor, cognitive, and language outcomes were assessed at a corrected age of 18 and 33 months and 4.5 years. Elastic Net was implemented to select the clinical and radiomic features that best predicted outcome. The area under the receiver operating characteristic (AUROC) curve was used to determine the predictive ability of each feature set. Clinical variables predicted cognitive outcome at 18 months with AUROC 0.76 and motor outcome at 4.5 years with AUROC 0.78. T1-radiomics features showed better prediction than T2-radiomics on the total motor outcome at 18 months and gross motor outcome at 33 months (AUROC: 0.81 vs 0.66 and 0.77 vs 0.7). T2-radiomics features were superior in two 4.5-year motor outcomes (AUROC: 0.78 vs 0.64 and 0.8 vs 0.57). Combining clinical parameters and radiomics features improved model performance in motor outcome at 4.5 years (AUROC: 0.84 vs 0.8). Radiomic features outperformed clinical variables for the prediction of adverse motor outcomes. Adding clinical variables to the radiomics model enhanced predictive performance.

## Introduction

Despite advances in medical care and improved survival, infants born preterm are at risk for abnormal brain development and long-term neurodevelopmental impairments^1^. These may arise in multiple functional domains including motor, cognitive, and language and continue beyond childhood and adolescence^1,2^. White matter injury (WMI) is identified in up to 50% of very preterm neonates and constitutes a characteristic brain injury pattern^3^. The severity of punctate WMI is best assessed on T1-weighted MRI acquired early in life before reaching term-equivalent age^4,5^. Previously, we found that, in very preterm neonates, WMI location and volume on early MRI predict cognitive and motor outcomes at 18 months of age^6^. However, manual volumetric segmentation of lesional burden relies on human experts and is cumbersome and time intensive.

Radiomics uses a quantitative set of features calculated from radiologic images to detect distinct quantifiable phenotypic differences of tissues^7^. Radiomic features quantify the intensity, texture and geometrical characteristics attributed to imaging data^8^. They are increasingly used for computer-aided pattern recognition and classification, as well as image-based diagnosis and prognosis^9^. Given the strong association between WMI and adverse outcomes, we hypothesized that radiomics predicts the neurodevelopmental outcome of preterm neonates at 18 months of age and beyond.

We therefore aimed to (i) investigate the predictive value of radiomics to adverse neurodevelopmental outcome of preterm neonates with or without WMI, and (ii) evaluate whether integrating the clinical variables, previously established as predictors of adverse neurodevelopmental outcome^10^, can enhance the prediction accuracy of radiomics in this population.

## Results

### Study population, clinical variables, and neurodevelopmental outcome

**Figure 1:**
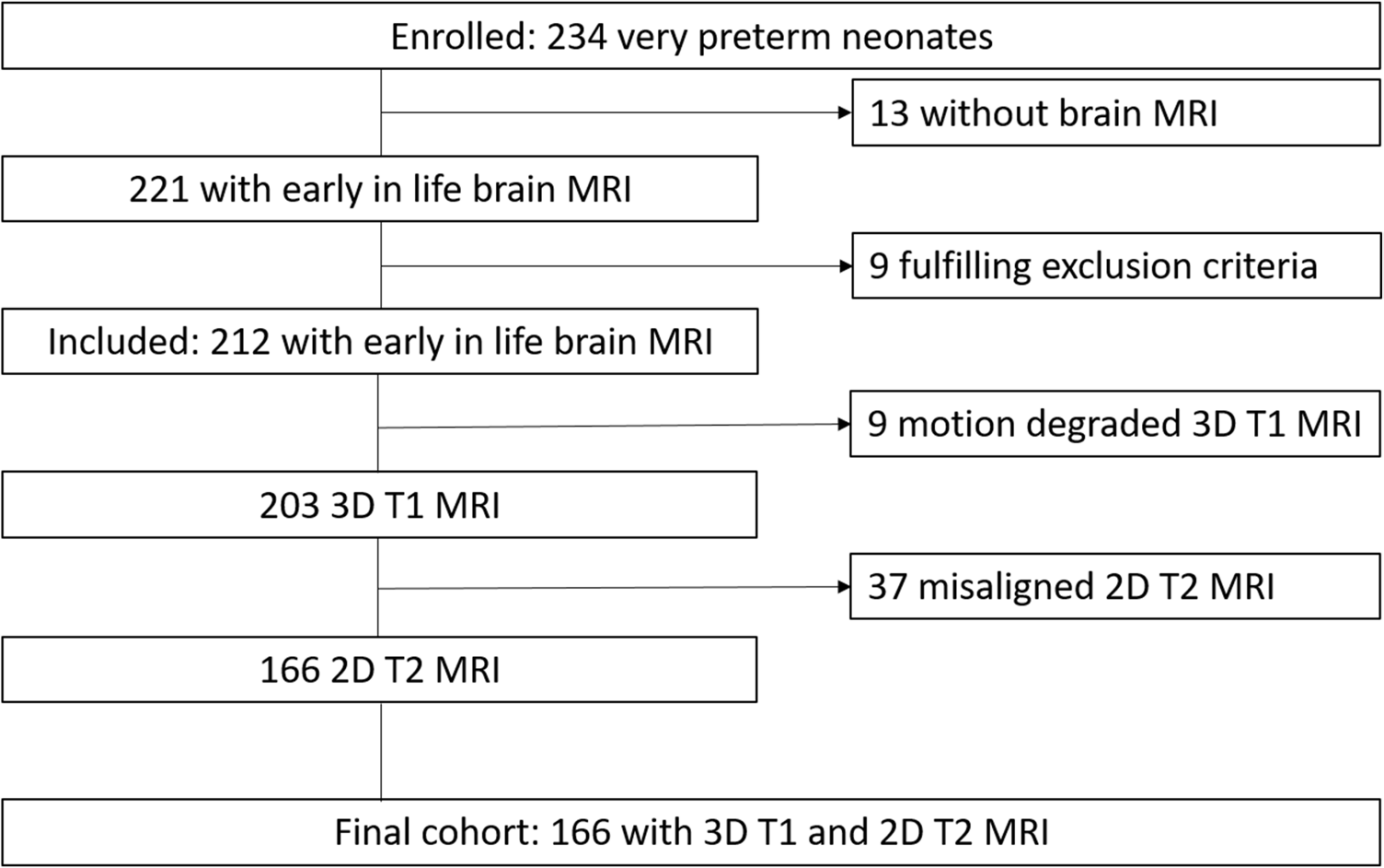
Patient and MRI sequence selection.

A total of 212 subjects were included in this study. Nine were excluded due to large parenchymal hemorrhagic infarctions, congenital malformations/syndromes, or antenatal infections. A non-motion degraded 3D T1-weighted sequence was available in 203 neonates and 166 of them had 2D axial T2-weighted sequence that were well aligned with their T1-weighted sequence. Therefore, the final study cohort consisted of 166 preterm neonates (median gestational age (GA): 28.1 weeks, 89 males) who had both T1- and T2 weighted sequences available (Fig. 1). Demographic and clinical variables of 166 preterm neonates are summarized in Table 1. No statistically significant differences (*p* > 0.05) in these variables were found between the 166 neonates included in the final analysis and those excluded (Supplemental Table 1). All available neurodevelopmental outcome parameters are summarized in Supplemental Table 2. Based on the early MRI of the brain, 36 neonates were identified with WMI (15 minimal, 14 moderate, 7 severe). Clinical factors as well as the level of maternal education did not differ in neonates with and without WMI (*p* > 0.05).

**Table 1:** Demographic and clinical variables.

Total		166 (100%)
	Male	89 (54%)
	Female	77 (46%)
Gestational age at birth (mean ± STD)	28 ± 2.2 weeks	
IVH grade 2–3	61 (37%)	
WMI	Total	36 (22%)
	Minimal	15 (9%)
	Moderate	14 (8%)
	Severe	7 (4%)
TCV (mean ± STD)	173.4 ± 38.6 cm3	
BPD		36 (22%)
NEC		
	Mild	30 (18%)
	Moderate	1 (0.01%)
	Severe	6 (4%)
Stoll's sepsis categorization		
	1	108 (65%)
	2	19 (11%)
	3	34 (20%)
	4	4 (2%)
	5	1 (0.01%)
ROP		
	Moderate	55 (33%)
	Severe	15 (9%)
DOL MRI 1 (mean ± STD)	29 ± 22 days	
Multiple infections	45 (27%)	
Maternal smoking		
	1 (< 20 cigarettes/day)	13 (8%)
	2 (> 20 cigarettes/day)	1 (0.01%)
Maternal illicit drug usage	13 (8%)	

*IVH* Intraventricular hemorrhage, *WMI* white matter injury, *TCV* total cerebral volume, *STD* standard deviation, *BDP* bronchopulmonary dysplasia, *NEC* necrotizing enterocolitis, *ROP* retinopathy of prematurity, *DOL MRI 1* day of life at 1st MRI.

**Table 2:** Area under the receiver operating characteristic for all Neurodevelopmental scores.

Outcome (N = 19)	Gestational age only	Clinical variables	T1-Radiomics	T2-Radiomics	T1/2-Radiomics	Clinical + radiomics
Bayley-III Cognitive Composite Score 18 M	0.72 (0.631–0.816)	**0.76 (0.66**–**0.854)**	0.63 (0.519–0.74)	0.69 (0.578–0.796)	0.69 (0.59–0.799)	**0.79 (0.708**–**0.874)**
Bayley-III Language Composite Score 18 M	0.66 (0.570–0.755)	0.6 (0.5–0.694)	0.64 (0.555–0.735)	0.5 (0.399–0.603)	0.66 (0.574–0.752)	0.64 (0.55–0.733)
Bayley-III Motor Composite Score 18 M	0.70 (0.615–0.789)	0.64 (0.551–0.736)	0.59 (0.493–0.687)	0.6 (0.5–0.692)	0.6 (0.507–0.702)	0.61 (0.509–0.704)
PDMS-2 fine motor quotient 18 M	0.69 (0.514–0.859)	0.58 (0.35–0.817)	0.52 (0.33–0.716)	0.49 (0.312–0.663)	0.34 (0.168–0.515)	0.45 (0.287–0.614)
PDMS-2 gross motor quotient 18 M	0.75 (0.665–0.828)	0.71 (0.626–0.8)	0.64 (0.55–0.737)	0.7 (0.604–0.786)	0.71 (0.618–0.794)	0.73 (0.647–0.818)
PDMS-2 total motor quotient 18 M	0.74 (0.657–0.83)	0.72 (0.618–0.826)	**0.81 (0.74**–**0.883)**	0.66 (0.56–0.759)	**0.81 (0.743**–**0.886)**	**0.83 (0.752**–**0.899)**
PDMS-2 fine motor quotient 33 M	0.69 (0.56–0.812)	0.53 (0.343–0.723)	0.55 (0.41–0.69)	0.54 (0.384–0.697)	0.56 (0.411–0.7)	0.53 (0.38–0.671)
PDMS-2 gross motor quotient 33 M	0.65 (0.537–0.762)	0.57 (0.443–0.697)	**0.77 (0.676**–**0.869)**	0.7 (0.595–0.807)	**0.75 (0.652**–**0.856)**	**0.75 (0.651**–**0.846)**
PDMS-2 total motor quotient 33 M	0.72 (0.619–0.827)	0.68 (0.555–0.807)	0.64 (0.52–0.766)	0.69 (0.565–0.813)	0.61 (0.478–0.743)	0.6 (0.471–0.728)
Bayley-III cognitive composite score 33 M	0.51 (0.353–0.667)	0.42 (0.261–0.575)	0.66 (0.503–0.81)	0.53 (0.387–0.678)	0.61 (0.436–0.779)	0.59 (0.418–0.765)
WPPSI-IV Full IQ 4.5Y	0.53 (0.397–0672)	0.49 (0.339–0.649)	0.58 (0.437–0.724)	0.49 (0.325–0.647)	0.51 (0.359–0.654)	0.51 (0.36–0.654)
Bayley-III Motor Composite Score 33 M	0.69 (0.58–0.792)	0.68 (0.548–0.817)	0.72 (0.621–0.822)	0.64 (0.505–0.78)	0.68 (0.567–0.79)	0.6 (0.477–0.727)
WPPSI-IV Performance IQ 4.5Y	0.66 (0.447–0.876)	0.53 (0.27–0.798)	0.6 (0.482–0.726)	0.7 (0.52–0.881)	0.68 (0.535–0.818)	0.6 (0.449–0.752)
WPPSI-IV Verbal IQ 4.5Y	0.55 (0.365–0.735)	0.5 (0.291–0.716)	0.52 (0.322–0.726)	0.37 (0.17–0.563)	0.49 (0.277–0.697)	0.48 (0.285–0.679)
PDMS-2 fine motor quotient 4.5Y	0.63 (0.455–0.811)	0.56 (0.369–0.757)	0.56 (0.376–0.739)	0.48 (0.293–0.666)	0.59 (0.412–0.759)	0.57 (0.396–0.745)
Bayley-III Language Composite Score 33 M	0.73 (0.561–0.889)	0.71 (0.492–0.919)	0.65 (0.468–0.824)	0.68 (0.506–0.854)	0.62 (0.394–0.856)	0.66 (0.457–0.863)
WPPSI-IV processing speed IQ 4.5Y	0.72 (0.594–0.838)	0.72 (0.585–0.845)	0.7 (0.566–0.829)	0.74 (0.612–0.872)	0.71 (0.583–0.836)	0.69 (0.565–0.823)
PDMS-2 gross motor quotient 4.5Y	**0.75 (0.579**–**0.912)**	**0.78 (0.629**–**0.927)**	0.64 (0.453–0.818)	**0.78 (0.635**–**0.934)**	**0.8 (0.659**–**0.932)**	**0.84 (0.729**–**0.956)**
PDMS-2 total motor quotient 4.5Y	0.66 (0.472–0.856)	0.73 (0.545–0.913)	0.57 (0.366–0.765)	**0.8 (0.654**–**0.945)**	0.69 (0.53–0.856)	0.71 (0.545–0.877)

Mean area under the receiver operating characteristic (AUROC) with 95% confidence interval (95% CI) are shown. AUROC ≥ 75% marked in bold. *PDMS-2* Peabody Developmental Motor Scales, second edition, *WPPSI-IV* Wechsler Primary and Preschool Scale of Intelligence, fourth edition, *IQ* intelligence quotient, *m* months, *y* years.

### Machine learning model performance in predicting neurodevelopmental outcomes

Table 2 shows the AUROC values for the prediction of adverse outcomes in preterm neonates based on 1) clinical variables, 2) T1-weighted radiomic features (T1-radiomics), 3) T2-weighted radiomic features (T2-radiomics), 4) combined T1- and T2-weighted radiomic features (T1/2-radiomics), and 5) combined clinical variables and T1/2-radiomics (clinical + radiomics). A clinically meaningful AUROC ≥ 0.75 ^11,12^ was reached for 5 of the 19 neurodevelopmental outcomes. Clinical variables alone reached an AUROC of 0.76 for the Bayley-III cognitive composite score at 18 months and 0.78 for the PDMS-2 gross motor quotient at 4.5 years. The T1-radiomics model achieved an AUROC of 0.81 for the PDMS-2 total motor quotient at 18 months and 0.77 for the PDMS-2 gross motor quotient at 33 months. The T2-radiomics model achieved an AUROC of 0.78 and 0.8 for the PDMS-2 gross and total motor quotient at 4.5 years, respectively. The T1/2-radiomics model reached an AUROC of 0.81 for the PDMS-2 total motor quotient at 18 months, 0.75 for the PDMS-2 gross motor quotient at 33 months, and 0.8 for the PDMS-2 gross motor quotient at 4.5 years. The combined clinical + radiomics prediction model achieved an AUROC of 0.79 for the Bayley-III cognitive composite score at 18 months, 0.83 for the PDMS-2 total motor quotient at 18 months, 0.75 for the PDMS-2 gross motor quotient at 33 months, and 0.84 for PDMS-2 gross motor quotient at 4.5 years. Supplementary Table 3 shows the random AUROC values based on permuted outcomes and suggests that these predicts are indeed significantly higher performing than random.

### Clinical and radiomic features

The five most relevant clinical, T1-radiomics, T2-radiomics, T1/2-radiomics, and clinical + radiomics parameters are shown in Supplemental Table 4.

The five best-performing *clinical variables* for Bayley-III cognitive composite score at 18 months were maternal smoking, NEC, multiple infections, GA at birth, and ROP. For PDMS-2 gross motor quotient at 4.5 years, the five best performing clinical parameters were maternal smoking, BPD, day of life at first MRI (DOL MRI1), Stoll’s sepsis categorization 2, and ROP.

The five best performing *T1-radiomics features* for PDMS-2 total motor quotient at 18 months were wavelet and gradient variants of one gray level size zone matrix (GLSZM) and four first order features. Similarly, for the PDMS-2 gross motor quotient at 33 months, the best features were all wavelet variants of four GLSZM and one first order feature.

The five best performing *T2-radiomics features* for the PDMS-2 gross motor quotient at 4.5 years were wavelet variants and local binary patterns of first order and GLSZM features. In addition, the original shape feature “Elongation” was found to be important. For the PDMS-2 total motor quotient at 4.5 years, the five best performing parameters were variants of first order and wavelet variants of GLSZM features.

The five best performing *T1/2-radiomics features* for the PDMS-2 total motor quotient at 18 months were wavelet variants of a first order and two GLSZM T1-radiomics features, a gradient variant of a first order T1-radiomics feature, and a wavelet variant of a T2-radiomics first order feature. For the PDMS-2 gross motor quotient at 33 months, the five best performing combined T1/2 radiomics features were all wavelet variants of three GLSZM and two first order features. For the PDMS-2 gross motor quotient at 4.5 years, they were wavelet variants of one GLSZM and one first order T1-radiomics feature and wavelet and two local binary pattern variants of two GLSZM and one first order T2-radiomics feature. A wavelet variant of GLSZM T1-radiomics feature was the top predictor among all for the PDMS-2 gross motor quotient at 4.5 years.

The top 5 performing combined *clinical* + *radiomics features* for the Bayley-III cognitive composite score at 18 months were maternal smoking, NEC, multiple infections, Stoll’s sepsis categorization 4, and a wavelet variant of one GLSZM T1-radiomics feature. For the PDMS-2 total motor quotient at 18 months, they were: ROP, multiple infections, NEC, a wavelet variant of one GLSZM T1-radiomics features, and Stoll’s sepsis categorization 3. For the PDMS-2 gross motor quotient at 33 months, they were multiple infections, wavelet variants of three GLSZM T1-radiomics features, and one root mean squared of a first order T2-radiomics feature. For the PDMS-2 gross motor quotient at 4.5 years, they were BPD, NEC, ROP, and wavelet variants of one GLSZM T1-radiomics feature and one GLSZM T2-radiomics feature.

## Discussion

In this prospective cohort of 166 very preterm neonates (36 with WMI), we applied fully automated radiomic feature analysis on T1- and T2-weighted sequences to predict neurodevelopmental outcomes. Radiomic features outperformed clinical variables for the prediction of adverse motor outcomes at 18 months, 33 months, and 4.5 years. Adding clinical variables to the radiomics model further enhanced its predictive strength for motor and cognitive outcomes.

Recently, Shin and Nam et al. used radiomics to predict Bayley II psychomotor outcome in 46 preterm neonates^13^. They found that near or term-equivalent synthetic T1-weighted images have a high diagnostic performance for the prediction of poor psychomotor outcome at 12 months corrected age. Notably, only 1 of 46 neonates had severe WMI. Our study is different from Shin and Nam et al. in several aspects. While we included a larger cohort for radiomics analysis (n = 166) with a higher percentage of WMI, we used conventional T1- and T2-weighted sequences, which are the mainstay of MRI acquisition. Our MRIs were acquired early in life (median PMA: 32 weeks, IQR: 30.4–33.6) when WMI is most readily visualized prior to reaching term-equivalent age. Also, we provide a more comprehensive analysis with regard to the neurodevelopmental assessments of motor, cognitive, and language skills, using several age-appropriate standard scores, including Bayley-III scales, PDMS-2, and WPPSI-IV, at three time points from toddlerhood to pre-school age, while Shin and Nam et al. predicted outcomes at 12 months corrected age only, where long term outcome prediction is limited^14^.

Our model performed best at predicting adverse motor outcome at 4.5 years of age, which has been shown to correlate with motor impairment in adolescence^15^. In our study, radiomics features predicted adverse outcome at the level of clinical variables or above. Adding clinical variables to radiomics features further enhanced outcome prediction. T2-radiomics had a better predictive value compared to T1-radiomics in the 4.5-year age group, while T1-radiomics performed at the level or slightly above T2-radiomics in the 18- and 33-month group. This finding needs to be interpreted in conjunction with the top performing radiomic features. The co-dominant feature sets using T1-weighted imaging were filters of first order and GLSZM features. GLSZM features assess variants of interconnected voxels of the same gray value independent of the angle of connectedness. Prior studies showed that multifocal WMI in preterm neonates is accompanied by altered white matter microstructure and disrupted white matter maturation^6,16^. Assuming that the WMI was the main driver of the adverse outcome prediction, we hypothesize, that the GLSZM quantify the degree and architecture of the T1-weighted shortening effect of the WMI. This is supported by the other top predictive features: kurtosis, minimum signal intensity, skewness, maximum signal intensity. These histogram features quantify the histogram curve shape (kurtosis, skewness) and quantify the highest (maximum) and lowest (minimum) intensity value in the images further emphasizing the key role of T1-weighted signal intensity differences in outcome prediction.

Due to the decreasing incidence of periventricular leukomalacia, diffuse white matter changes are now the most common abnormality of the preterm brain^17^. Using MRI acquired at term-equivalent age, Parikh et al. showed that T2-weighted quantification of diffuse white matter abnormality was a significant prognostic biomarker of motor development at 3 years of age in very preterm infants^18^. In our study, the two top performing features for motor outcome prediction at 4.5 years of life were a filtered variant of the first order feature “Maximum signal intensity” indicating that abnormally increased T2-weighted signal intensity in the brain plays an important role for motor outcome prediction at 4.5 years. The importance of increased T2-weighted signal is further supported by another top performing T2-radiomics feature: a variant of the first order histogram parameter 90th percentile. Notably, the shape feature elongation was predictive of outcome for both gross and total motor outcomes at 4.5 years. Elongation measures the ratio between the minor and major axes of the region of interest^19^. When applying this calculation to the whole brain in 2D plane, this translates to a surrogate of the brain circumference. When comparing to the predictions discussed above, the combined T1/2-radiomics model did not yield further relevant increases in predictive performance. However, it does further emphasize the importance of first order and GLSZM features in motor outcome prediction at 4.5 years.

Of all models tested in our study, the combined model of clinical parameters and radiomics yielded the best AUROC values. Except for the gross motor quotient at 33 months, outcome parameters were predicted with a higher AUROC using the combined model. Clinical variables predictive of outcome were similar to outcome prediction without radiomics. Regarding radiomics features, we again noted first order and GLSZM features to be the most predictive. Additionally, T2-radiomics were more influential in the outcome prediction at older age.

There are some limitations to this study. First, although our cohort is one of the largest very preterm study cohorts, the data set was still relatively small (n = 166) for employing machine learning techniques. Second, we adopted LOOCV method to evaluate the predictive value of our approach for outcomes and did not use another independent cohort dataset to validate the model performance. Third, longer term follow-up is under way and has not yet been available.

In this prospective cohort of very preterm neonates with and without WMI imaged early in life, we applied fully automated radiomic feature analysis on T1- and T2-weighted sequences to predict neurodevelopmental outcomes. Radiomic features outperformed clinical variables for the prediction of adverse motor outcomes at 18 months, 33 months, and 4.5 years. Adding clinical variables to the radiomics model enhanced its predictive strength for motor and cognitive outcomes. MRI-based radiomics of preterm brain MRI improves neurodevelopmental outcome prediction beyond 18 and 33 months.

## Methods

### Study participants and MRI protocol

Over a 7-year period (2006–2012), 234 very preterm neonates (122 males) born between 24–32 weeks’ gestation (median 27.7 weeks) were admitted to the neonatal intensive care unit at British Columbia’s Women’s Hospital, Vancouver, Canada and were enrolled prospectively. A total of 221 early (median postmenstrual age: 32 weeks, interquartile range [IQR] 30.4–33.6) brain MRIs were acquired on a Siemens MAGNETOM Avanto 1.5 T MRI scanner (Erlangen, Germany). All newborns were scanned without pharmacological sedation using an MR-compatible isolette (Lammers Medical Technology, Lübeck, Germany) and specialized neonatal head coil (Advanced Imaging Research, Cleveland, OH) as soon as they were clinically stable. The MR imaging protocol included a three dimensional (3D) volumetric T1-weighted sequence (repetition time [TR]: 36 ms, echo time [TE]: 9.2 ms, field of view [FOV]: 200 mm, slice thickness: 1 mm, no gap), and a two dimensional (2D) axial fast spin echo T2-weighted sequence (TR: 4610 ms, TE: 107 ms, FOV: 160 mm, slice thickness: 4 mm, gap: 0.2 mm)^6,20^. Neonates with large parenchymal hemorrhagic infarctions (> 2 cm), congenital malformations/syndromes, or antenatal infections were excluded.

### Clinical variables

Clinical data about pregnancy, delivery, and perinatal course were collected through systematic prospective chart reviews. This included clinical factors previously associated with impaired neurodevelopmental outcomes such as presence of moderate to severe intraventricular hemorrhage (IVH grade 2–3), severity of WMI (minimal: ≤ 3 lesions of < 2 mm, moderate: > 3 lesions or lesions > 2 mm and < 5% hemispheric involvement, severe: > 5% of the hemisphere according to^6^), total cerebral volume (TCV), bronchopulmonary dysplasia (BPD) (defined as the need for supplemental oxygen at 36 weeks’ postmenstrual age), with/without multiple infections (≥ 3 infections), necrotizing enterocolitis (NEC), retinopathy of prematurity (ROP), and Stoll’s sepsis categorization^21^. Variables for individuals who were missing maternal smoking (n = 2) and maternal illicit drug usage (n = 2) were imputed using the mode, grouped by alcohol status where applicable. Multiple infections (n = 1) and BPD (n = 3) were imputed with random forest with birth GA. Imputations were performed using the simputation R package. https://cran.r-project.org/web/packages/simputation/simputation.pdf.

### Neurodevelopmental outcomes

A total of 183 (86%) 169 infants (80%), and 169 (80%) infants had a clinical follow-up at 18 and 33 months corrected age (CA), as well as 4.5 years, respectively (median age 18.7 months, interquartile range [IQR] 18.3–19.2, median age 34.7 months, IQR 33.8–36.2, and median age 4.83 years, IQR 4.75–4.91). The Bayley Scales of Infant and Toddler Development, third edition (Bayley-III) including standardized composite scores for motor, cognitive, and language skills with means of 100 and standard deviation (SD) of 15^22^ were used to assess children’s neurodevelopmental abilities at 18 and 33 months CA. A score of more than 1 SD below the mean (i.e. < 85) was considered to reflect adverse outcome. A score equal to or above 85 was considered typical neurodevelopmental outcome. In addition, the Peabody Developmental Motor Scales, second edition (PDMS-2) was used to assess total, gross and fine motor quotient values in children at the three follow-ups as a more robust assessment of motor impairment^23^. Having scores of less than 80 was considered as being clinically impaired. The Wechsler Primary and Preschool Scale of Intelligence, fourth edition (WPPSI-IV) was used to evaluate cognitive function of the study participants at 4.5 years of age. With a mean of 100 and SD of 15, the full-scale IQ of less than 85 was considered to reflect adverse cognitive outcome at 4.5 years. Assessments were carried out by qualified examiners who were blinded to the MRI of the preterm neonates.

### Radiomic feature extraction process

MRI data were exported to an offline workstation. A brain mask of the cerebrum was created for the early-in-life T1-weighted image of each neonate using MAGeT-Brain pipeline^24^. The segmented brain masks were reviewed and manually revised for areas that were not accurately segmented by the automatic pipeline. After resampling and co-registering the T2-weighted sequence to the T1-weighted image of the same subject, the brain mask was applied to the T1- and T2-weighted sequences and non-brain tissue components were removed. Then, bias field correction and z-score normalization were used to standardize the range of all image features^25,26^. Radiomics features were defined according to the PyRadiomics Python package, version 3.0^19^. All features available from the PyRadiomics package were considered after removing features with zero variance. A total of 1428 and 1056 radiomic features remained from the T1- and T2-weighted images, respectively. Radiomic features included histogram, shape, and texture features with and without local binary pattern, Laplacian of Gaussian filter, and wavelet-based filter. Since the T2-weighted sequence was not considered a 3D sequence, 3D wavelet-based filters were not extracted for these images. All extracted features are summarized in Supplemental Tables 5 and 6.

### Machine learning model and statistical analysis

Elastic Net was implemented as a form of penalized logistic regression consisting of least absolute shrinkage and selection operator (LASSO) and Ridge regression. It includes hyperparameters, which determine the degree to which the sum of either the squared (Ridge) or absolute (LASSO) slopes is penalized. Elastic Net is well suited to select the features that best predict the outcome^27,28^. For each of the 19 outcomes, we examined the predictive ability of the following feature sets: 1) clinical variables, 2) T1-weighted radiomic features (T1-radiomics), 3) T2-weighted radiomic features (T2-radiomics), 4) combined T1- and T2-weighted radiomic features (T1/2-radiomics), and 5) combined clinical variables and T1/2-radiomics (clinical + radiomics). A leave-one-out cross-validation (LOOCV) scheme was used with the R caret package, with remaining parameters passed to “glmnet” as default^29^. The area under the receiver operating characteristic (AUROC) curve was used to determine the predictive ability of each feature set. 95% confidence intervals were calculated using the pROC R package^30^. An additional model run was conducted on permuted outcomes to enable a comparison of our results with random predictions. An AUROC ≥ 0.75 was considered as clinically meaningful ^11,12^.

### Ethics approval and subject consent

The Clinical Research Ethics Board at the University of British Columbia and BC Children’s and Women’s Hospitals reviewed and approved the study protocol. The study protocol was performed in accordance with the Declaration of Helsinki. A parent or legal guardian provided written informed consent.

## Supplementary Information

Supplementary Information.

## Data Availability

The datasets generated during and/or analysed during the current study are available from the corresponding author on reasonable request.
