# Supplementary material for: MRI based radiomics enhances prediction of neurodevelopmental outcome in very preterm neonates
Source: Sci Rep. 2022 Jul 13;12:11872. doi: 10.1038/s41598-022-16066-w (PMC9279296; doi:10.1038/s41598-022-16066-w)
Supplement: Supplementary file 1 — Supplementary Information. [file 41598_2022_16066_MOESM1_ESM.docx]

| Supplemental Table 1. Demographic and clinical variables for neonates included and excluded in the final analysis | | | | |
| --- | --- | --- | --- | --- |
|  | **Included N=166** | **Excluded N=68** | **P** |  |
| Male sex, n (%) | 89 (54%) | 33 (49%) | 0.57 |  |
| Gestational age at birth,  weeks, median [IQR] | 28.1 [26-29.7]​ | 27.5 [26.1-29]​ | ​0.26 |  |
| IVH grade 2-3, n (%) | 61 (36.7%)​ | 14 (30.4%)​ | ​0.49 |  |
| TCV, ml, median [IQR]​ | 168.7 [146.8-199.7]​ | 169.6 [146.5-201.6]​ | ​0.86 |  |
| BPD, n (%) | 36 (22%)​ | 19 (29%)​ | 0.3 |  |
| NEC | 37 (22.3%)​ | 14 (20.9%) | 0.86 |  |
| Stoll's sepsis score, n (%) |  |  |  |  |
| None | 108 (65.1%) | 46 (68.7%) |  |  |
| Mild/moderate | 53 (31.9%) | 19 (28.4%) | 0.89 |  |
| Severe | 5 (3%) | 2 (3%) |  |  |
| ROP, n (%) |  |  |  |  |
| None | 96 (58%) | 38 (58%) |  |  |
| Mild/moderate | 55 (33%) | 21 (32%) | 0.93 |  |
| Severe | 15 (9%) | 7 (11%) |  |  |
| Maternal smoking, n (%) |  |  |  |  |
| None | 147 (91.3%) | 60 (89.6%) |  |  |
| <20 per day | 13 (8.1%) | 6 (9%) | 0.79 |  |
| ≥20 per day | 1 (0.6%) | 1 (1.5%) |  |  |
| Maternal illicit drug  usage, n (%) | 13 (8%) | 2 (3%) | 0.24 |  |

Legend: Interquartile range (IQR), intraventricular hemorrhage (IVH), total cerebral volume (TCV), bronchopulmonary dysplasia (BDP), necrotizing enterocolitis (NEC), retinopathy of prematurity (ROP).

| Supplemental Table 2. Available neurodevelopmental scores, number of observations, and binarized outcomes | | | | |
| --- | --- | --- | --- | --- |
|  |  |  |  |  |
| Outcome (n=19) | **Patients with both T1- and T2-weighted sequences** | | | |
|  | **All** | **Favorable** | **Adverse** | **Prevalence (%)** |
| Bayley-III cognitive composite score 18m | 143 | 111 | 32 | 22.38 |
| Bayley-III language composite score 18m | 142 | 89 | 53 | 37.32 |
| Bayley-III motor composite score 18m | 142 | 88 | 54 | 38.03 |
| PDMS-2 fine motor quotient 18m | 140 | 129 | 11 | 7.86 |
| PDMS-2 gross motor quotient 18m | 140 | 84 | 56 | 40.00 |
| PDMS-2 total motor quotient 18m | 140 | 100 | 40 | 28.57 |
| Bayley-III cognitive composite score 33m | 132 | 116 | 16 | 12.12 |
| Bayley-III language composite score 33m | 115 | 104 | 11 | 9.57 |
| Bayley-III motor composite score 33m | 127 | 103 | 24 | 18.90 |
| PDMS-2 fine motor quotient 33m | 136 | 121 | 15 | 11.03 |
| PDMS-2 gross motor quotient 33m | 134 | 106 | 28 | 20.90 |
| PDMS-2 total motor quotient 33m | 134 | 110 | 24 | 17.91 |
| WPPSI-IV full IQ 4.5y | 132 | 114 | 18 | 13.64 |
| WPPSI-IV performance IQ 4.5y | 125 | 117 | 8 | 6.40 |
| WPPSI-IV verbal IQ 4.5y | 124 | 112 | 12 | 9.68 |
| WPPSI-IV processing speed IQ 4.5y | 111 | 94 | 17 | 15.32 |
| PDMS-2 fine motor quotient 4.5y | 122 | 110 | 12 | 9.84 |
| PDMS-2 gross motor quotient 4.5y | 45 | 30 | 15 | 33.33 |
| PDMS-2 total motor quotient 4.5y | 44 | 32 | 12 | 27.27 |

Legend: Peabody Developmental Motor Scales, second edition (PDMS-2), Wechsler Primary and Preschool Scale of Intelligence, fourth edition (WPPSI-IV), Intelligence Quotient (IQ), months (M), years (Y).

| Supplemental Table 3. Permuted aurocS WITH ci. AUROC > 0.7 sHOWN IN BOLD. | | | | | | |
| --- | --- | --- | --- | --- | --- | --- |
| Outcome (n=19) | **Gestational age only** | **Clinical variables** | **T1-radiomics** | **T2-radiomics** | **T1/2-radiomics** | **Clinical + radiomics** |
| Bayley-III cognitive composite score 18m | 0.59 (0.48 – 0.70) | 0.51 (0.38 – 0.64) | 0.63 (0.52 - 0.74) | 0.61 (0.50 - 0.72) | 0.47 (0.36 – 0.59) | 0.57 (0.45 – 0.69) |
| Bayley-III language composite score 18m | 0.55 (0.45 – 0.65) | 0.58 (0.48 – 0.67) | 0.53 (0.43 – 0.63) | 0.56 (0.46 – 0.67) | 0.53 (0.43 – 0.63) | 0.54 (0.44 – 0.64) |
| Bayley-III motor composite score 18m | 0.49 (0.40 – 0.59) | 0.47 (0.37 – 0.57) | 0.54 (0.44 – 0.64) | 0.44 (0.34 – 0.53) | 0.55 (0.46 – 0.65) | 0.53 (0.43 – 0.63) |
| PDMS-2 fine motor quotient 18m | 0.62 (0.47 – 0.77) | 0.54 (0.34 – 0.74) | 0.56 (0.36 – 0.77) | 0.59 (0.39 – 0.80) | 0.61 (0.51 – 0.71) | 0.52 (0.32 – 0.72) |
| PDMS-2 gross motor quotient 18m | 0.56 (0.47 – 0.66) | 0.52 (0.42 – 0.62) | 0.54 (0.44 – 0.64) | 0.48 (0.39 – 0.58) | 0.56 (0.46 – 0.66) | 0.57 (0.47 – 0.67) |
| PDMS-2 total motor quotient 18m | 0.56 (0.46 – 0.67) | 0.56 (0.45 – 0.68) | 0.53 (0.42 – 0.63) | 0.56 (0.45 – 0.67) | 0.53 (0.43 – 0.63) | 0.55 (0.45 – 0.66) |
| PDMS-2 fine motor quotient 33m | 0.54 (0.38 – 0.71) | **0.73 (0.59 – 0.87)** | 0.53 (0.37 – 0.68) | 0.58 (0.43 – 0.73) | 0.54 (0.38 – 0.70) | 0.50 (0.32 – 0.68) |
| PDMS-2 gross motor quotient 33m | 0.55 (0.43 – 0.68) | 0.56 (0.45 – 0.67) | 0.50 (0.38 – 0.62) | 0.55 (0.42 – 0.68) | 0.54 (0.42 – 0.65) | 0.52 (0.41 – 0.62) |
| PDMS-2 total motor quotient 33m | 0.53 (0.41 – 0.66) | 0.55 (0.41 – 0.70) | 0.56 (0.43 – 0.69) | 0.55 (0.42 – 0.69) | 0.50 (0.36 – 0.63) | 0.63 (0.51 – 0.76) |
| Bayley-III cognitive composite score 33m | 0.56 (0.41 – 0.71) | 0.56 (0.41 – 0.71) | 0.68 (0.54 – 0.82) | 0.67 (0.52 – 0.83) | 0.55 (0.41 – 0.70) | 0.52 (0.36 – 0.67) |
| WPPSI-IV full IQ 4.5y | 0.45 (0.30 – 0.61) | 0.47 (0.32 – 0.63) | 0.56 (0.41 – 0.71) | 0.61 (0.48 – 0.75) | 0.49 (0.35 – 0.63) | 0.62 (0.47 – 0.78) |
| Bayley-III motor composite score 33m | 0.60 (0.48 – 0.73) | 0.53 (0.40 – 0.66) | 0.50 (0.36 – 0.64) | 0.50 (0.37 – 0.64) | 0.57 (0.45 – 0.69) | 0.55 (0.41 – 0.70) |
| WPPSI-IV performance IQ 4.5y | 0.48 (0.27 – 0.68) | 0.57 (0.39 – 0.76) | 0.49 (0.34 – 0.63) | **0.70 (0.56 – 0.84)** | 0.57 (0.37 – 0.76) | 0.55 (0.31 – 0.79) |
| WPPSI-IV verbal IQ 4.5y | 0.53 (0.36 – 0.70) | 0.52 (0.35 – 0.69) | 0.63 (0.49 – 0.77) | 0.55 (0.38 – 0.72) | 0.48 (0.31 – 0.64) | 0.56 (0.38 – 0.75) |
| PDMS-2 fine motor quotient 4.5y | 0.58 (0.40 – 0.75) | 0.49 (0.33 – 0.66) | 0.65 (0.50 – 0.80) | 0.67 (0.51 – 0.83) | 0.60 (0.40 – 0.81) | 0.56 (0.39 – 0.73) |
| Bayley-III language composite score 33m | 0.56 (0.34 – 0.77) | 0.51 (0.29 – 0.73) | 0.64 (0.45 – 0.83) | 0.53 (0.33 – 0.73) | 0.52 (0.35 – 0.69) | 0.62 (0.50 – 0.75) |
| WPPSI-IV processing speed IQ 4.5y | 0.49 (0.32 – 0.65) | 0.60 (0.43 – 0.77) | 0.51 (0.36 – 0.67) | 0.50 (0.34 – 0.66) | 0.55 (0.39 – 0.70) | 0.55 (0.41 – 0.69) |
| PDMS-2 gross motor quotient 4.5y | 0.44 (0.36 – 0.73) | 0.57 (0.40 – 0.75) | 0.55 (0.36 – 0.75) | 0.48 (0.30 – 0.67) | 0.50 (0.29 – 0.71) | 0.52 (0.33 – 0.70) |
| PDMS-2 total motor quotient 4.5y | 0.58 (0.41 – 0.76) | 0.57 (0.38 – 0.75) | 0.56 (0.36 – 0.76) | 0.60 (0.41 – 0.79) | 0.59 (0.41 – 0.77) | 0.61 (0.44 – 0.78) |

| Supplemental Table 4. top 5 radiomic or clinical features for adverse neurodevelopmental outcomes auroc ≥ 0.75 | | | | | | |
| --- | --- | --- | --- | --- | --- | --- |
| *Clinical variables* | *AUROC (95% CI)* | *Top 1* | *Top 2* | *Top 3* | *Top 4* | *Top 5* |
| Bayley-III cognitive composite score 18m | **0.76 (0.665 - 0.854)** | Maternal smoking 1 | NEC (moderate) | Multiple infections | Gestational age at birth | ROP (severe) |
| PDMS-2 gross motor quotient 4.5y | **0.78 (0.629 - 0.927)** | Maternal smoking 1 | BPD | DOL MRI 1 | Stoll's sepsis score 2 | ROP (severe) |
|  |  |  |  |  |  |  |
| *T1-radiomics* |  |  |  |  |  |  |
| PDMS-2 total motor quotient 18m | **0.81 (0.74 - 0.883)** | wavelet-LHH glszm SizeZoneNonUniformityNormalized | gradient firstorder Minimum | wavelet-LHH firstorder Skewness | wavelet-HHL firstorder Skewness | wavelet-LHL firstorder Maximum |
| PDMS-2 gross motor quotient 33m | **0.77 (0.676 - 0.869)** | wavelet-HHH firstorder Kurtosis | wavelet-HLH glszm ZonePercentage | wavelet-HHH glszm SizeZoneNonUniformityNormalized | wavelet-HHH glszm HighGrayLevelZoneEmphasis | wavelet-HHH glszm LowGrayLevelZoneEmphasis |
|  |  |  |  |  |  |  |
| *T2-radiomics* |  |  |  |  |  |  |
| PDMS-2 gross motor quotient 4.5y | **0.78 (0.635 - 0.934)** | wavelet-LH firstorder Maximum | lbp-2D firstorder 90Percentile | original shape Elongation | wavelet-LH firstorder TotalEnergy | wavelet-HH glszm HighGrayLevelZoneEmphasis |
| PDMS-2 total motor quotient 4.5y | **0.8 (0.654 - 0.945)** | wavelet-LH firstorder Maximum | original shape Elongation | wavelet-LL glszm SmallAreaLowGrayLevelEmphasis | wavelet-HL firstorder Skewness | square firstorder Minimum |
|  |  |  |  |  |  |  |
| *T1/2-radiomics* |  |  |  |  |  |  |
| PDMS-2 total motor quotient 18m | **0.81 (0.743 - 0.886)** | T1-wavelet-LHH glszm SizeZoneNonUniformityNormalized | T1-gradient firstorder Minimum | T1-wavelet-LLL firstorder Minimum | T2-wavelet-HH firstorder Skewness | T1-wavelet-HHH glszm ZoneVariance |
| PDMS-2 gross motor quotient 33m | **0.75 (0.652 - 0.856)** | T1-wavelet-HLH firstorder Skewness | T1-wavelet-LHH glszm SizeZoneNonUniformityNormalized | T1-wavelet-HHH firstorder Kurtosis | T1-wavelet-HHL glszm SizeZoneNonUniformityNormalized | T1-wavelet-HHH glszm HighGrayLevelZoneEmphasis |
| PDMS-2 gross motor quotient 4.5y | **0.8 (0.659 - 0.932)** | T1-wavelet-HHH glszm SmallAreaHighGrayLevelEmphasis | T2-lbp-3D-k firstorder 10Percentile | T2-wavelet-HH glszm ZonePercentage | T1-wavelet-HHL firstorder Skewness | T2-wavelet-LL glszm SmallAreaLowGrayLevelEmphasis |
|  |  |  |  |  |  |  |
| *Clinical + radiomics* |  |  |  |  |  |  |
| Bayley-III cognitive composite score 18m | **0.79 (0.708 - 0.874)** | Maternal smoking 1 | NEC (moderate) | Multiple infections | Stoll's sepsis score 4 | T1 wavelet-LHH glszm SizeZoneNonUniformityNormalized |
| PDMS-2 total motor quotient 18m | **0.83 (0.752 - 0.899)** | ROP (severe) | Multiple infections | NEC (mild) | T1 wavelet-LHH glszm SizeZoneNonUniformityNormalized | Stoll's sepsis score 3 |
| PDMS-2 gross motor quotient 33m | **0.75 (0.651 - 0.846)** | Multiple infections | T1 wavelet-HHH glszm SizeZoneNonUniformityNormalized | T1 wavelet-LHH glszm SizeZoneNonUniformityNormalized | T1 wavelet-HHL glszm SizeZoneNonUniformityNormalized | T2 square firstorder Energy |
| PDMS-2 gross motor quotient 4.5y | **0.84 (0.729 - 0.956)** | BPD | NEC (mild) | T1 wavelet-HHH glszm SmallAreaHighGrayLevelEmphasis | ROP (severe) | T2 wavelet-HH glszm ZonePercentage |

Legend: Area under the receiver operating characteristic (AUROC), confidence interval (CI), Peabody Developmental Motor Scales, second edition (PDMS-2), Wechsler Primary and Preschool Scale of Intelligence, fourth edition (WPPSI-IV), intelligence quotient (IQ), months (m), years (y), bronchopulmonary dysplasia (BPD), retinopathy of prematurity (ROP), necrotizing enterocolitis (NEC), day of life at 1st MRI (DOL MRI 1). Top features determined by absolute value of the coefficient in each predictive model.

| Supplemental Table 5. T1-radiomics features | | | |
| --- | --- | --- | --- |
|  |  |  |  |
| Filter | **Feature type** | **Feature** |  |
| original | shape | Elongation |  |
| original | shape | Flatness |  |
| original | shape | LeastAxisLength |  |
| original | shape | MajorAxisLength |  |
| original | shape | Maximum2DDiameterColumn |  |
| original | shape | Maximum2DDiameterRow |  |
| original | shape | Maximum2DDiameterSlice |  |
| original | shape | Maximum3DDiameter |  |
| original | shape | MeshVolume |  |
| original | shape | MinorAxisLength |  |
| original | shape | Sphericity |  |
| original | shape | SurfaceArea |  |
| original | shape | SurfaceVolumeRatio |  |
| original | shape | VoxelVolume |  |
| original | glcm | Autocorrelation |  |
| original | glcm | ClusterProminence |  |
| original | glcm | ClusterShade |  |
| original | glcm | ClusterTendency |  |
| original | glcm | Contrast |  |
| original | glcm | Correlation |  |
| original | glcm | DifferenceAverage |  |
| original | glcm | DifferenceEntropy |  |
| original | glcm | DifferenceVariance |  |
| original | glcm | Id |  |
| original | glcm | Idm |  |
| original | glcm | Idmn |  |
| original | glcm | Idn |  |
| original | glcm | Imc1 |  |
| original | glcm | Imc2 |  |
| original | glcm | InverseVariance |  |
| original | glcm | JointAverage |  |
| original | glcm | JointEnergy |  |
| original | glcm | JointEntropy |  |
| original | glcm | MCC |  |
| original | glcm | MaximumProbability |  |
| original | glcm | SumAverage |  |
| original | glcm | SumEntropy |  |
| original | glcm | SumSquares |  |
| original | glrlm | GrayLevelNonUniformity |  |
| original | glrlm | GrayLevelNonUniformityNormalized |  |
| original | glrlm | GrayLevelVariance |  |
| original | glrlm | HighGrayLevelRunEmphasis |  |
| original | glrlm | LongRunEmphasis |  |
| original | glrlm | LongRunHighGrayLevelEmphasis |  |
| original | glrlm | LongRunLowGrayLevelEmphasis |  |
| original | glrlm | LowGrayLevelRunEmphasis |  |
| original | glrlm | RunEntropy |  |
| original | glrlm | RunLengthNonUniformity |  |
| original | glrlm | RunLengthNonUniformityNormalized |  |
| original | glrlm | RunPercentage |  |
| original | glrlm | RunVariance |  |
| original | glrlm | ShortRunEmphasis |  |
| original | glrlm | ShortRunHighGrayLevelEmphasis |  |
| original | glrlm | ShortRunLowGrayLevelEmphasis |  |
| original | firstorder | 10Percentile |  |
| original | firstorder | 90Percentile |  |
| original | firstorder | Energy |  |
| original | firstorder | Entropy |  |
| original | firstorder | InterquartileRange |  |
| original | firstorder | Kurtosis |  |
| original | firstorder | Maximum |  |
| original | firstorder | MeanAbsoluteDeviation |  |
| original | firstorder | Mean |  |
| original | firstorder | Median |  |
| original | firstorder | Minimum |  |
| original | firstorder | Range |  |
| original | firstorder | RobustMeanAbsoluteDeviation |  |
| original | firstorder | RootMeanSquared |  |
| original | firstorder | Skewness |  |
| original | firstorder | TotalEnergy |  |
| original | firstorder | Uniformity |  |
| original | firstorder | Variance |  |
| original | glszm | GrayLevelNonUniformity |  |
| original | glszm | GrayLevelNonUniformityNormalized |  |
| original | glszm | GrayLevelVariance |  |
| original | glszm | HighGrayLevelZoneEmphasis |  |
| original | glszm | LargeAreaEmphasis |  |
| original | glszm | LargeAreaHighGrayLevelEmphasis |  |
| original | glszm | LargeAreaLowGrayLevelEmphasis |  |
| original | glszm | LowGrayLevelZoneEmphasis |  |
| original | glszm | SizeZoneNonUniformity |  |
| original | glszm | SizeZoneNonUniformityNormalized |  |
| original | glszm | SmallAreaEmphasis |  |
| original | glszm | SmallAreaHighGrayLevelEmphasis |  |
| original | glszm | SmallAreaLowGrayLevelEmphasis |  |
| original | glszm | ZoneEntropy |  |
| original | glszm | ZonePercentage |  |
| original | glszm | ZoneVariance |  |
| original | gldm | DependenceEntropy |  |
| original | gldm | DependenceNonUniformity |  |
| original | gldm | DependenceNonUniformityNormalized |  |
| original | gldm | DependenceVariance |  |
| original | gldm | GrayLevelNonUniformity |  |
| original | gldm | GrayLevelVariance |  |
| original | gldm | HighGrayLevelEmphasis |  |
| original | gldm | LargeDependenceEmphasis |  |
| original | gldm | LargeDependenceHighGrayLevelEmphasis |  |
| original | gldm | LargeDependenceLowGrayLevelEmphasis |  |
| original | gldm | LowGrayLevelEmphasis |  |
| original | gldm | SmallDependenceEmphasis |  |
| original | gldm | SmallDependenceHighGrayLevelEmphasis |  |
| original | gldm | SmallDependenceLowGrayLevelEmphasis |  |
| original | ngtdm | Busyness |  |
| original | ngtdm | Coarseness |  |
| original | ngtdm | Complexity |  |
| original | ngtdm | Contrast |  |
| original | ngtdm | Strength |  |
| wavelet-LLH | glcm | Autocorrelation |  |
| wavelet-LLH | glcm | ClusterProminence |  |
| wavelet-LLH | glcm | ClusterShade |  |
| wavelet-LLH | glcm | ClusterTendency |  |
| wavelet-LLH | glcm | Contrast |  |
| wavelet-LLH | glcm | Correlation |  |
| wavelet-LLH | glcm | DifferenceAverage |  |
| wavelet-LLH | glcm | DifferenceEntropy |  |
| wavelet-LLH | glcm | DifferenceVariance |  |
| wavelet-LLH | glcm | Id |  |
| wavelet-LLH | glcm | Idm |  |
| wavelet-LLH | glcm | Idmn |  |
| wavelet-LLH | glcm | Idn |  |
| wavelet-LLH | glcm | Imc1 |  |
| wavelet-LLH | glcm | Imc2 |  |
| wavelet-LLH | glcm | InverseVariance |  |
| wavelet-LLH | glcm | JointAverage |  |
| wavelet-LLH | glcm | JointEnergy |  |
| wavelet-LLH | glcm | JointEntropy |  |
| wavelet-LLH | glcm | MCC |  |
| wavelet-LLH | glcm | MaximumProbability |  |
| wavelet-LLH | glcm | SumAverage |  |
| wavelet-LLH | glcm | SumEntropy |  |
| wavelet-LLH | glcm | SumSquares |  |
| wavelet-LLH | glrlm | GrayLevelNonUniformity |  |
| wavelet-LLH | glrlm | GrayLevelNonUniformityNormalized |  |
| wavelet-LLH | glrlm | GrayLevelVariance |  |
| wavelet-LLH | glrlm | HighGrayLevelRunEmphasis |  |
| wavelet-LLH | glrlm | LongRunEmphasis |  |
| wavelet-LLH | glrlm | LongRunHighGrayLevelEmphasis |  |
| wavelet-LLH | glrlm | LongRunLowGrayLevelEmphasis |  |
| wavelet-LLH | glrlm | LowGrayLevelRunEmphasis |  |
| wavelet-LLH | glrlm | RunEntropy |  |
| wavelet-LLH | glrlm | RunLengthNonUniformity |  |
| wavelet-LLH | glrlm | RunLengthNonUniformityNormalized |  |
| wavelet-LLH | glrlm | RunPercentage |  |
| wavelet-LLH | glrlm | RunVariance |  |
| wavelet-LLH | glrlm | ShortRunEmphasis |  |
| wavelet-LLH | glrlm | ShortRunHighGrayLevelEmphasis |  |
| wavelet-LLH | glrlm | ShortRunLowGrayLevelEmphasis |  |
| wavelet-LLH | firstorder | 10Percentile |  |
| wavelet-LLH | firstorder | 90Percentile |  |
| wavelet-LLH | firstorder | Energy |  |
| wavelet-LLH | firstorder | Entropy |  |
| wavelet-LLH | firstorder | InterquartileRange |  |
| wavelet-LLH | firstorder | Kurtosis |  |
| wavelet-LLH | firstorder | Maximum |  |
| wavelet-LLH | firstorder | MeanAbsoluteDeviation |  |
| wavelet-LLH | firstorder | Mean |  |
| wavelet-LLH | firstorder | Median |  |
| wavelet-LLH | firstorder | Minimum |  |
| wavelet-LLH | firstorder | Range |  |
| wavelet-LLH | firstorder | RobustMeanAbsoluteDeviation |  |
| wavelet-LLH | firstorder | RootMeanSquared |  |
| wavelet-LLH | firstorder | Skewness |  |
| wavelet-LLH | firstorder | TotalEnergy |  |
| wavelet-LLH | firstorder | Uniformity |  |
| wavelet-LLH | firstorder | Variance |  |
| wavelet-LLH | glszm | GrayLevelNonUniformity |  |
| wavelet-LLH | glszm | GrayLevelNonUniformityNormalized |  |
| wavelet-LLH | glszm | GrayLevelVariance |  |
| wavelet-LLH | glszm | HighGrayLevelZoneEmphasis |  |
| wavelet-LLH | glszm | LargeAreaEmphasis |  |
| wavelet-LLH | glszm | LargeAreaHighGrayLevelEmphasis |  |
| wavelet-LLH | glszm | LargeAreaLowGrayLevelEmphasis |  |
| wavelet-LLH | glszm | LowGrayLevelZoneEmphasis |  |
| wavelet-LLH | glszm | SizeZoneNonUniformity |  |
| wavelet-LLH | glszm | SizeZoneNonUniformityNormalized |  |
| wavelet-LLH | glszm | SmallAreaEmphasis |  |
| wavelet-LLH | glszm | SmallAreaHighGrayLevelEmphasis |  |
| wavelet-LLH | glszm | SmallAreaLowGrayLevelEmphasis |  |
| wavelet-LLH | glszm | ZoneEntropy |  |
| wavelet-LLH | glszm | ZonePercentage |  |
| wavelet-LLH | glszm | ZoneVariance |  |
| wavelet-LLH | gldm | DependenceEntropy |  |
| wavelet-LLH | gldm | DependenceNonUniformity |  |
| wavelet-LLH | gldm | DependenceNonUniformityNormalized |  |
| wavelet-LLH | gldm | DependenceVariance |  |
| wavelet-LLH | gldm | GrayLevelNonUniformity |  |
| wavelet-LLH | gldm | GrayLevelVariance |  |
| wavelet-LLH | gldm | HighGrayLevelEmphasis |  |
| wavelet-LLH | gldm | LargeDependenceEmphasis |  |
| wavelet-LLH | gldm | LargeDependenceHighGrayLevelEmphasis |  |
| wavelet-LLH | gldm | LargeDependenceLowGrayLevelEmphasis |  |
| wavelet-LLH | gldm | LowGrayLevelEmphasis |  |
| wavelet-LLH | gldm | SmallDependenceEmphasis |  |
| wavelet-LLH | gldm | SmallDependenceHighGrayLevelEmphasis |  |
| wavelet-LLH | gldm | SmallDependenceLowGrayLevelEmphasis |  |
| wavelet-LLH | ngtdm | Busyness |  |
| wavelet-LLH | ngtdm | Coarseness |  |
| wavelet-LLH | ngtdm | Complexity |  |
| wavelet-LLH | ngtdm | Contrast |  |
| wavelet-LLH | ngtdm | Strength |  |
| wavelet-LHL | glcm | Autocorrelation |  |
| wavelet-LHL | glcm | ClusterProminence |  |
| wavelet-LHL | glcm | ClusterShade |  |
| wavelet-LHL | glcm | ClusterTendency |  |
| wavelet-LHL | glcm | Contrast |  |
| wavelet-LHL | glcm | Correlation |  |
| wavelet-LHL | glcm | DifferenceAverage |  |
| wavelet-LHL | glcm | DifferenceEntropy |  |
| wavelet-LHL | glcm | DifferenceVariance |  |
| wavelet-LHL | glcm | Id |  |
| wavelet-LHL | glcm | Idm |  |
| wavelet-LHL | glcm | Idmn |  |
| wavelet-LHL | glcm | Idn |  |
| wavelet-LHL | glcm | Imc1 |  |
| wavelet-LHL | glcm | Imc2 |  |
| wavelet-LHL | glcm | InverseVariance |  |
| wavelet-LHL | glcm | JointAverage |  |
| wavelet-LHL | glcm | JointEnergy |  |
| wavelet-LHL | glcm | JointEntropy |  |
| wavelet-LHL | glcm | MCC |  |
| wavelet-LHL | glcm | MaximumProbability |  |
| wavelet-LHL | glcm | SumAverage |  |
| wavelet-LHL | glcm | SumEntropy |  |
| wavelet-LHL | glcm | SumSquares |  |
| wavelet-LHL | glrlm | GrayLevelNonUniformity |  |
| wavelet-LHL | glrlm | GrayLevelNonUniformityNormalized |  |
| wavelet-LHL | glrlm | GrayLevelVariance |  |
| wavelet-LHL | glrlm | HighGrayLevelRunEmphasis |  |
| wavelet-LHL | glrlm | LongRunEmphasis |  |
| wavelet-LHL | glrlm | LongRunHighGrayLevelEmphasis |  |
| wavelet-LHL | glrlm | LongRunLowGrayLevelEmphasis |  |
| wavelet-LHL | glrlm | LowGrayLevelRunEmphasis |  |
| wavelet-LHL | glrlm | RunEntropy |  |
| wavelet-LHL | glrlm | RunLengthNonUniformity |  |
| wavelet-LHL | glrlm | RunLengthNonUniformityNormalized |  |
| wavelet-LHL | glrlm | RunPercentage |  |
| wavelet-LHL | glrlm | RunVariance |  |
| wavelet-LHL | glrlm | ShortRunEmphasis |  |
| wavelet-LHL | glrlm | ShortRunHighGrayLevelEmphasis |  |
| wavelet-LHL | glrlm | ShortRunLowGrayLevelEmphasis |  |
| wavelet-LHL | firstorder | 10Percentile |  |
| wavelet-LHL | firstorder | 90Percentile |  |
| wavelet-LHL | firstorder | Energy |  |
| wavelet-LHL | firstorder | Entropy |  |
| wavelet-LHL | firstorder | InterquartileRange |  |
| wavelet-LHL | firstorder | Kurtosis |  |
| wavelet-LHL | firstorder | Maximum |  |
| wavelet-LHL | firstorder | MeanAbsoluteDeviation |  |
| wavelet-LHL | firstorder | Mean |  |
| wavelet-LHL | firstorder | Median |  |
| wavelet-LHL | firstorder | Minimum |  |
| wavelet-LHL | firstorder | Range |  |
| wavelet-LHL | firstorder | RobustMeanAbsoluteDeviation |  |
| wavelet-LHL | firstorder | RootMeanSquared |  |
| wavelet-LHL | firstorder | Skewness |  |
| wavelet-LHL | firstorder | TotalEnergy |  |
| wavelet-LHL | firstorder | Uniformity |  |
| wavelet-LHL | firstorder | Variance |  |
| wavelet-LHL | glszm | GrayLevelNonUniformity |  |
| wavelet-LHL | glszm | GrayLevelNonUniformityNormalized |  |
| wavelet-LHL | glszm | GrayLevelVariance |  |
| wavelet-LHL | glszm | HighGrayLevelZoneEmphasis |  |
| wavelet-LHL | glszm | LargeAreaEmphasis |  |
| wavelet-LHL | glszm | LargeAreaHighGrayLevelEmphasis |  |
| wavelet-LHL | glszm | LargeAreaLowGrayLevelEmphasis |  |
| wavelet-LHL | glszm | LowGrayLevelZoneEmphasis |  |
| wavelet-LHL | glszm | SizeZoneNonUniformity |  |
| wavelet-LHL | glszm | SizeZoneNonUniformityNormalized |  |
| wavelet-LHL | glszm | SmallAreaEmphasis |  |
| wavelet-LHL | glszm | SmallAreaHighGrayLevelEmphasis |  |
| wavelet-LHL | glszm | SmallAreaLowGrayLevelEmphasis |  |
| wavelet-LHL | glszm | ZoneEntropy |  |
| wavelet-LHL | glszm | ZonePercentage |  |
| wavelet-LHL | glszm | ZoneVariance |  |
| wavelet-LHL | gldm | DependenceEntropy |  |
| wavelet-LHL | gldm | DependenceNonUniformity |  |
| wavelet-LHL | gldm | DependenceNonUniformityNormalized |  |
| wavelet-LHL | gldm | DependenceVariance |  |
| wavelet-LHL | gldm | GrayLevelNonUniformity |  |
| wavelet-LHL | gldm | GrayLevelVariance |  |
| wavelet-LHL | gldm | HighGrayLevelEmphasis |  |
| wavelet-LHL | gldm | LargeDependenceEmphasis |  |
| wavelet-LHL | gldm | LargeDependenceHighGrayLevelEmphasis |  |
| wavelet-LHL | gldm | LargeDependenceLowGrayLevelEmphasis |  |
| wavelet-LHL | gldm | LowGrayLevelEmphasis |  |
| wavelet-LHL | gldm | SmallDependenceEmphasis |  |
| wavelet-LHL | gldm | SmallDependenceHighGrayLevelEmphasis |  |
| wavelet-LHL | gldm | SmallDependenceLowGrayLevelEmphasis |  |
| wavelet-LHL | ngtdm | Busyness |  |
| wavelet-LHL | ngtdm | Coarseness |  |
| wavelet-LHL | ngtdm | Complexity |  |
| wavelet-LHL | ngtdm | Contrast |  |
| wavelet-LHL | ngtdm | Strength |  |
| wavelet-LHH | glcm | Autocorrelation |  |
| wavelet-LHH | glcm | ClusterProminence |  |
| wavelet-LHH | glcm | ClusterShade |  |
| wavelet-LHH | glcm | ClusterTendency |  |
| wavelet-LHH | glcm | Contrast |  |
| wavelet-LHH | glcm | Correlation |  |
| wavelet-LHH | glcm | DifferenceAverage |  |
| wavelet-LHH | glcm | DifferenceEntropy |  |
| wavelet-LHH | glcm | DifferenceVariance |  |
| wavelet-LHH | glcm | Id |  |
| wavelet-LHH | glcm | Idm |  |
| wavelet-LHH | glcm | Idmn |  |
| wavelet-LHH | glcm | Idn |  |
| wavelet-LHH | glcm | Imc1 |  |
| wavelet-LHH | glcm | Imc2 |  |
| wavelet-LHH | glcm | InverseVariance |  |
| wavelet-LHH | glcm | JointAverage |  |
| wavelet-LHH | glcm | JointEnergy |  |
| wavelet-LHH | glcm | JointEntropy |  |
| wavelet-LHH | glcm | MCC |  |
| wavelet-LHH | glcm | MaximumProbability |  |
| wavelet-LHH | glcm | SumAverage |  |
| wavelet-LHH | glcm | SumEntropy |  |
| wavelet-LHH | glcm | SumSquares |  |
| wavelet-LHH | glrlm | GrayLevelNonUniformity |  |
| wavelet-LHH | glrlm | GrayLevelNonUniformityNormalized |  |
| wavelet-LHH | glrlm | GrayLevelVariance |  |
| wavelet-LHH | glrlm | HighGrayLevelRunEmphasis |  |
| wavelet-LHH | glrlm | LongRunEmphasis |  |
| wavelet-LHH | glrlm | LongRunHighGrayLevelEmphasis |  |
| wavelet-LHH | glrlm | LongRunLowGrayLevelEmphasis |  |
| wavelet-LHH | glrlm | LowGrayLevelRunEmphasis |  |
| wavelet-LHH | glrlm | RunEntropy |  |
| wavelet-LHH | glrlm | RunLengthNonUniformity |  |
| wavelet-LHH | glrlm | RunLengthNonUniformityNormalized |  |
| wavelet-LHH | glrlm | RunPercentage |  |
| wavelet-LHH | glrlm | RunVariance |  |
| wavelet-LHH | glrlm | ShortRunEmphasis |  |
| wavelet-LHH | glrlm | ShortRunHighGrayLevelEmphasis |  |
| wavelet-LHH | glrlm | ShortRunLowGrayLevelEmphasis |  |
| wavelet-LHH | firstorder | 10Percentile |  |
| wavelet-LHH | firstorder | 90Percentile |  |
| wavelet-LHH | firstorder | Energy |  |
| wavelet-LHH | firstorder | Entropy |  |
| wavelet-LHH | firstorder | InterquartileRange |  |
| wavelet-LHH | firstorder | Kurtosis |  |
| wavelet-LHH | firstorder | Maximum |  |
| wavelet-LHH | firstorder | MeanAbsoluteDeviation |  |
| wavelet-LHH | firstorder | Mean |  |
| wavelet-LHH | firstorder | Median |  |
| wavelet-LHH | firstorder | Minimum |  |
| wavelet-LHH | firstorder | Range |  |
| wavelet-LHH | firstorder | RobustMeanAbsoluteDeviation |  |
| wavelet-LHH | firstorder | RootMeanSquared |  |
| wavelet-LHH | firstorder | Skewness |  |
| wavelet-LHH | firstorder | TotalEnergy |  |
| wavelet-LHH | firstorder | Uniformity |  |
| wavelet-LHH | firstorder | Variance |  |
| wavelet-LHH | glszm | GrayLevelNonUniformity |  |
| wavelet-LHH | glszm | GrayLevelNonUniformityNormalized |  |
| wavelet-LHH | glszm | GrayLevelVariance |  |
| wavelet-LHH | glszm | HighGrayLevelZoneEmphasis |  |
| wavelet-LHH | glszm | LargeAreaEmphasis |  |
| wavelet-LHH | glszm | LargeAreaHighGrayLevelEmphasis |  |
| wavelet-LHH | glszm | LargeAreaLowGrayLevelEmphasis |  |
| wavelet-LHH | glszm | LowGrayLevelZoneEmphasis |  |
| wavelet-LHH | glszm | SizeZoneNonUniformity |  |
| wavelet-LHH | glszm | SizeZoneNonUniformityNormalized |  |
| wavelet-LHH | glszm | SmallAreaEmphasis |  |
| wavelet-LHH | glszm | SmallAreaHighGrayLevelEmphasis |  |
| wavelet-LHH | glszm | SmallAreaLowGrayLevelEmphasis |  |
| wavelet-LHH | glszm | ZoneEntropy |  |
| wavelet-LHH | glszm | ZonePercentage |  |
| wavelet-LHH | glszm | ZoneVariance |  |
| wavelet-LHH | gldm | DependenceEntropy |  |
| wavelet-LHH | gldm | DependenceNonUniformity |  |
| wavelet-LHH | gldm | DependenceNonUniformityNormalized |  |
| wavelet-LHH | gldm | DependenceVariance |  |
| wavelet-LHH | gldm | GrayLevelNonUniformity |  |
| wavelet-LHH | gldm | GrayLevelVariance |  |
| wavelet-LHH | gldm | HighGrayLevelEmphasis |  |
| wavelet-LHH | gldm | LargeDependenceEmphasis |  |
| wavelet-LHH | gldm | LargeDependenceHighGrayLevelEmphasis |  |
| wavelet-LHH | gldm | LargeDependenceLowGrayLevelEmphasis |  |
| wavelet-LHH | gldm | LowGrayLevelEmphasis |  |
| wavelet-LHH | gldm | SmallDependenceEmphasis |  |
| wavelet-LHH | gldm | SmallDependenceHighGrayLevelEmphasis |  |
| wavelet-LHH | gldm | SmallDependenceLowGrayLevelEmphasis |  |
| wavelet-LHH | ngtdm | Busyness |  |
| wavelet-LHH | ngtdm | Coarseness |  |
| wavelet-LHH | ngtdm | Complexity |  |
| wavelet-LHH | ngtdm | Contrast |  |
| wavelet-LHH | ngtdm | Strength |  |
| wavelet-HLL | glcm | Autocorrelation |  |
| wavelet-HLL | glcm | ClusterProminence |  |
| wavelet-HLL | glcm | ClusterShade |  |
| wavelet-HLL | glcm | ClusterTendency |  |
| wavelet-HLL | glcm | Contrast |  |
| wavelet-HLL | glcm | Correlation |  |
| wavelet-HLL | glcm | DifferenceAverage |  |
| wavelet-HLL | glcm | DifferenceEntropy |  |
| wavelet-HLL | glcm | DifferenceVariance |  |
| wavelet-HLL | glcm | Id |  |
| wavelet-HLL | glcm | Idm |  |
| wavelet-HLL | glcm | Idmn |  |
| wavelet-HLL | glcm | Idn |  |
| wavelet-HLL | glcm | Imc1 |  |
| wavelet-HLL | glcm | Imc2 |  |
| wavelet-HLL | glcm | InverseVariance |  |
| wavelet-HLL | glcm | JointAverage |  |
| wavelet-HLL | glcm | JointEnergy |  |
| wavelet-HLL | glcm | JointEntropy |  |
| wavelet-HLL | glcm | MCC |  |
| wavelet-HLL | glcm | MaximumProbability |  |
| wavelet-HLL | glcm | SumAverage |  |
| wavelet-HLL | glcm | SumEntropy |  |
| wavelet-HLL | glcm | SumSquares |  |
| wavelet-HLL | glrlm | GrayLevelNonUniformity |  |
| wavelet-HLL | glrlm | GrayLevelNonUniformityNormalized |  |
| wavelet-HLL | glrlm | GrayLevelVariance |  |
| wavelet-HLL | glrlm | HighGrayLevelRunEmphasis |  |
| wavelet-HLL | glrlm | LongRunEmphasis |  |
| wavelet-HLL | glrlm | LongRunHighGrayLevelEmphasis |  |
| wavelet-HLL | glrlm | LongRunLowGrayLevelEmphasis |  |
| wavelet-HLL | glrlm | LowGrayLevelRunEmphasis |  |
| wavelet-HLL | glrlm | RunEntropy |  |
| wavelet-HLL | glrlm | RunLengthNonUniformity |  |
| wavelet-HLL | glrlm | RunLengthNonUniformityNormalized |  |
| wavelet-HLL | glrlm | RunPercentage |  |
| wavelet-HLL | glrlm | RunVariance |  |
| wavelet-HLL | glrlm | ShortRunEmphasis |  |
| wavelet-HLL | glrlm | ShortRunHighGrayLevelEmphasis |  |
| wavelet-HLL | glrlm | ShortRunLowGrayLevelEmphasis |  |
| wavelet-HLL | firstorder | 10Percentile |  |
| wavelet-HLL | firstorder | 90Percentile |  |
| wavelet-HLL | firstorder | Energy |  |
| wavelet-HLL | firstorder | Entropy |  |
| wavelet-HLL | firstorder | InterquartileRange |  |
| wavelet-HLL | firstorder | Kurtosis |  |
| wavelet-HLL | firstorder | Maximum |  |
| wavelet-HLL | firstorder | MeanAbsoluteDeviation |  |
| wavelet-HLL | firstorder | Mean |  |
| wavelet-HLL | firstorder | Median |  |
| wavelet-HLL | firstorder | Minimum |  |
| wavelet-HLL | firstorder | Range |  |
| wavelet-HLL | firstorder | RobustMeanAbsoluteDeviation |  |
| wavelet-HLL | firstorder | RootMeanSquared |  |
| wavelet-HLL | firstorder | Skewness |  |
| wavelet-HLL | firstorder | TotalEnergy |  |
| wavelet-HLL | firstorder | Uniformity |  |
| wavelet-HLL | firstorder | Variance |  |
| wavelet-HLL | glszm | GrayLevelNonUniformity |  |
| wavelet-HLL | glszm | GrayLevelNonUniformityNormalized |  |
| wavelet-HLL | glszm | GrayLevelVariance |  |
| wavelet-HLL | glszm | HighGrayLevelZoneEmphasis |  |
| wavelet-HLL | glszm | LargeAreaEmphasis |  |
| wavelet-HLL | glszm | LargeAreaHighGrayLevelEmphasis |  |
| wavelet-HLL | glszm | LargeAreaLowGrayLevelEmphasis |  |
| wavelet-HLL | glszm | LowGrayLevelZoneEmphasis |  |
| wavelet-HLL | glszm | SizeZoneNonUniformity |  |
| wavelet-HLL | glszm | SizeZoneNonUniformityNormalized |  |
| wavelet-HLL | glszm | SmallAreaEmphasis |  |
| wavelet-HLL | glszm | SmallAreaHighGrayLevelEmphasis |  |
| wavelet-HLL | glszm | SmallAreaLowGrayLevelEmphasis |  |
| wavelet-HLL | glszm | ZoneEntropy |  |
| wavelet-HLL | glszm | ZonePercentage |  |
| wavelet-HLL | glszm | ZoneVariance |  |
| wavelet-HLL | gldm | DependenceEntropy |  |
| wavelet-HLL | gldm | DependenceNonUniformity |  |
| wavelet-HLL | gldm | DependenceNonUniformityNormalized |  |
| wavelet-HLL | gldm | DependenceVariance |  |
| wavelet-HLL | gldm | GrayLevelNonUniformity |  |
| wavelet-HLL | gldm | GrayLevelVariance |  |
| wavelet-HLL | gldm | HighGrayLevelEmphasis |  |
| wavelet-HLL | gldm | LargeDependenceEmphasis |  |
| wavelet-HLL | gldm | LargeDependenceHighGrayLevelEmphasis |  |
| wavelet-HLL | gldm | LargeDependenceLowGrayLevelEmphasis |  |
| wavelet-HLL | gldm | LowGrayLevelEmphasis |  |
| wavelet-HLL | gldm | SmallDependenceEmphasis |  |
| wavelet-HLL | gldm | SmallDependenceHighGrayLevelEmphasis |  |
| wavelet-HLL | gldm | SmallDependenceLowGrayLevelEmphasis |  |
| wavelet-HLL | ngtdm | Busyness |  |
| wavelet-HLL | ngtdm | Coarseness |  |
| wavelet-HLL | ngtdm | Complexity |  |
| wavelet-HLL | ngtdm | Contrast |  |
| wavelet-HLL | ngtdm | Strength |  |
| wavelet-HLH | glcm | Autocorrelation |  |
| wavelet-HLH | glcm | ClusterProminence |  |
| wavelet-HLH | glcm | ClusterShade |  |
| wavelet-HLH | glcm | ClusterTendency |  |
| wavelet-HLH | glcm | Contrast |  |
| wavelet-HLH | glcm | Correlation |  |
| wavelet-HLH | glcm | DifferenceAverage |  |
| wavelet-HLH | glcm | DifferenceEntropy |  |
| wavelet-HLH | glcm | DifferenceVariance |  |
| wavelet-HLH | glcm | Id |  |
| wavelet-HLH | glcm | Idm |  |
| wavelet-HLH | glcm | Idmn |  |
| wavelet-HLH | glcm | Idn |  |
| wavelet-HLH | glcm | Imc1 |  |
| wavelet-HLH | glcm | Imc2 |  |
| wavelet-HLH | glcm | InverseVariance |  |
| wavelet-HLH | glcm | JointAverage |  |
| wavelet-HLH | glcm | JointEnergy |  |
| wavelet-HLH | glcm | JointEntropy |  |
| wavelet-HLH | glcm | MCC |  |
| wavelet-HLH | glcm | MaximumProbability |  |
| wavelet-HLH | glcm | SumAverage |  |
| wavelet-HLH | glcm | SumEntropy |  |
| wavelet-HLH | glcm | SumSquares |  |
| wavelet-HLH | glrlm | GrayLevelNonUniformity |  |
| wavelet-HLH | glrlm | GrayLevelNonUniformityNormalized |  |
| wavelet-HLH | glrlm | GrayLevelVariance |  |
| wavelet-HLH | glrlm | HighGrayLevelRunEmphasis |  |
| wavelet-HLH | glrlm | LongRunEmphasis |  |
| wavelet-HLH | glrlm | LongRunHighGrayLevelEmphasis |  |
| wavelet-HLH | glrlm | LongRunLowGrayLevelEmphasis |  |
| wavelet-HLH | glrlm | LowGrayLevelRunEmphasis |  |
| wavelet-HLH | glrlm | RunEntropy |  |
| wavelet-HLH | glrlm | RunLengthNonUniformity |  |
| wavelet-HLH | glrlm | RunLengthNonUniformityNormalized |  |
| wavelet-HLH | glrlm | RunPercentage |  |
| wavelet-HLH | glrlm | RunVariance |  |
| wavelet-HLH | glrlm | ShortRunEmphasis |  |
| wavelet-HLH | glrlm | ShortRunHighGrayLevelEmphasis |  |
| wavelet-HLH | glrlm | ShortRunLowGrayLevelEmphasis |  |
| wavelet-HLH | firstorder | 10Percentile |  |
| wavelet-HLH | firstorder | 90Percentile |  |
| wavelet-HLH | firstorder | Energy |  |
| wavelet-HLH | firstorder | Entropy |  |
| wavelet-HLH | firstorder | InterquartileRange |  |
| wavelet-HLH | firstorder | Kurtosis |  |
| wavelet-HLH | firstorder | Maximum |  |
| wavelet-HLH | firstorder | MeanAbsoluteDeviation |  |
| wavelet-HLH | firstorder | Mean |  |
| wavelet-HLH | firstorder | Median |  |
| wavelet-HLH | firstorder | Minimum |  |
| wavelet-HLH | firstorder | Range |  |
| wavelet-HLH | firstorder | RobustMeanAbsoluteDeviation |  |
| wavelet-HLH | firstorder | RootMeanSquared |  |
| wavelet-HLH | firstorder | Skewness |  |
| wavelet-HLH | firstorder | TotalEnergy |  |
| wavelet-HLH | firstorder | Uniformity |  |
| wavelet-HLH | firstorder | Variance |  |
| wavelet-HLH | glszm | GrayLevelNonUniformity |  |
| wavelet-HLH | glszm | GrayLevelNonUniformityNormalized |  |
| wavelet-HLH | glszm | GrayLevelVariance |  |
| wavelet-HLH | glszm | HighGrayLevelZoneEmphasis |  |
| wavelet-HLH | glszm | LargeAreaEmphasis |  |
| wavelet-HLH | glszm | LargeAreaHighGrayLevelEmphasis |  |
| wavelet-HLH | glszm | LargeAreaLowGrayLevelEmphasis |  |
| wavelet-HLH | glszm | LowGrayLevelZoneEmphasis |  |
| wavelet-HLH | glszm | SizeZoneNonUniformity |  |
| wavelet-HLH | glszm | SizeZoneNonUniformityNormalized |  |
| wavelet-HLH | glszm | SmallAreaEmphasis |  |
| wavelet-HLH | glszm | SmallAreaHighGrayLevelEmphasis |  |
| wavelet-HLH | glszm | SmallAreaLowGrayLevelEmphasis |  |
| wavelet-HLH | glszm | ZoneEntropy |  |
| wavelet-HLH | glszm | ZonePercentage |  |
| wavelet-HLH | glszm | ZoneVariance |  |
| wavelet-HLH | gldm | DependenceEntropy |  |
| wavelet-HLH | gldm | DependenceNonUniformity |  |
| wavelet-HLH | gldm | DependenceNonUniformityNormalized |  |
| wavelet-HLH | gldm | DependenceVariance |  |
| wavelet-HLH | gldm | GrayLevelNonUniformity |  |
| wavelet-HLH | gldm | GrayLevelVariance |  |
| wavelet-HLH | gldm | HighGrayLevelEmphasis |  |
| wavelet-HLH | gldm | LargeDependenceEmphasis |  |
| wavelet-HLH | gldm | LargeDependenceHighGrayLevelEmphasis |  |
| wavelet-HLH | gldm | LargeDependenceLowGrayLevelEmphasis |  |
| wavelet-HLH | gldm | LowGrayLevelEmphasis |  |
| wavelet-HLH | gldm | SmallDependenceEmphasis |  |
| wavelet-HLH | gldm | SmallDependenceHighGrayLevelEmphasis |  |
| wavelet-HLH | gldm | SmallDependenceLowGrayLevelEmphasis |  |
| wavelet-HLH | ngtdm | Busyness |  |
| wavelet-HLH | ngtdm | Coarseness |  |
| wavelet-HLH | ngtdm | Complexity |  |
| wavelet-HLH | ngtdm | Contrast |  |
| wavelet-HLH | ngtdm | Strength |  |
| wavelet-HHL | glcm | Autocorrelation |  |
| wavelet-HHL | glcm | ClusterProminence |  |
| wavelet-HHL | glcm | ClusterShade |  |
| wavelet-HHL | glcm | ClusterTendency |  |
| wavelet-HHL | glcm | Contrast |  |
| wavelet-HHL | glcm | Correlation |  |
| wavelet-HHL | glcm | DifferenceAverage |  |
| wavelet-HHL | glcm | DifferenceEntropy |  |
| wavelet-HHL | glcm | DifferenceVariance |  |
| wavelet-HHL | glcm | Id |  |
| wavelet-HHL | glcm | Idm |  |
| wavelet-HHL | glcm | Idmn |  |
| wavelet-HHL | glcm | Idn |  |
| wavelet-HHL | glcm | Imc1 |  |
| wavelet-HHL | glcm | Imc2 |  |
| wavelet-HHL | glcm | InverseVariance |  |
| wavelet-HHL | glcm | JointAverage |  |
| wavelet-HHL | glcm | JointEnergy |  |
| wavelet-HHL | glcm | JointEntropy |  |
| wavelet-HHL | glcm | MCC |  |
| wavelet-HHL | glcm | MaximumProbability |  |
| wavelet-HHL | glcm | SumAverage |  |
| wavelet-HHL | glcm | SumEntropy |  |
| wavelet-HHL | glcm | SumSquares |  |
| wavelet-HHL | glrlm | GrayLevelNonUniformity |  |
| wavelet-HHL | glrlm | GrayLevelNonUniformityNormalized |  |
| wavelet-HHL | glrlm | GrayLevelVariance |  |
| wavelet-HHL | glrlm | HighGrayLevelRunEmphasis |  |
| wavelet-HHL | glrlm | LongRunEmphasis |  |
| wavelet-HHL | glrlm | LongRunHighGrayLevelEmphasis |  |
| wavelet-HHL | glrlm | LongRunLowGrayLevelEmphasis |  |
| wavelet-HHL | glrlm | LowGrayLevelRunEmphasis |  |
| wavelet-HHL | glrlm | RunEntropy |  |
| wavelet-HHL | glrlm | RunLengthNonUniformity |  |
| wavelet-HHL | glrlm | RunLengthNonUniformityNormalized |  |
| wavelet-HHL | glrlm | RunPercentage |  |
| wavelet-HHL | glrlm | RunVariance |  |
| wavelet-HHL | glrlm | ShortRunEmphasis |  |
| wavelet-HHL | glrlm | ShortRunHighGrayLevelEmphasis |  |
| wavelet-HHL | glrlm | ShortRunLowGrayLevelEmphasis |  |
| wavelet-HHL | firstorder | 10Percentile |  |
| wavelet-HHL | firstorder | 90Percentile |  |
| wavelet-HHL | firstorder | Energy |  |
| wavelet-HHL | firstorder | Entropy |  |
| wavelet-HHL | firstorder | InterquartileRange |  |
| wavelet-HHL | firstorder | Kurtosis |  |
| wavelet-HHL | firstorder | Maximum |  |
| wavelet-HHL | firstorder | MeanAbsoluteDeviation |  |
| wavelet-HHL | firstorder | Mean |  |
| wavelet-HHL | firstorder | Median |  |
| wavelet-HHL | firstorder | Minimum |  |
| wavelet-HHL | firstorder | Range |  |
| wavelet-HHL | firstorder | RobustMeanAbsoluteDeviation |  |
| wavelet-HHL | firstorder | RootMeanSquared |  |
| wavelet-HHL | firstorder | Skewness |  |
| wavelet-HHL | firstorder | TotalEnergy |  |
| wavelet-HHL | firstorder | Uniformity |  |
| wavelet-HHL | firstorder | Variance |  |
| wavelet-HHL | glszm | GrayLevelNonUniformity |  |
| wavelet-HHL | glszm | GrayLevelNonUniformityNormalized |  |
| wavelet-HHL | glszm | GrayLevelVariance |  |
| wavelet-HHL | glszm | HighGrayLevelZoneEmphasis |  |
| wavelet-HHL | glszm | LargeAreaEmphasis |  |
| wavelet-HHL | glszm | LargeAreaHighGrayLevelEmphasis |  |
| wavelet-HHL | glszm | LargeAreaLowGrayLevelEmphasis |  |
| wavelet-HHL | glszm | LowGrayLevelZoneEmphasis |  |
| wavelet-HHL | glszm | SizeZoneNonUniformity |  |
| wavelet-HHL | glszm | SizeZoneNonUniformityNormalized |  |
| wavelet-HHL | glszm | SmallAreaEmphasis |  |
| wavelet-HHL | glszm | SmallAreaHighGrayLevelEmphasis |  |
| wavelet-HHL | glszm | SmallAreaLowGrayLevelEmphasis |  |
| wavelet-HHL | glszm | ZoneEntropy |  |
| wavelet-HHL | glszm | ZonePercentage |  |
| wavelet-HHL | glszm | ZoneVariance |  |
| wavelet-HHL | gldm | DependenceEntropy |  |
| wavelet-HHL | gldm | DependenceNonUniformity |  |
| wavelet-HHL | gldm | DependenceNonUniformityNormalized |  |
| wavelet-HHL | gldm | DependenceVariance |  |
| wavelet-HHL | gldm | GrayLevelNonUniformity |  |
| wavelet-HHL | gldm | GrayLevelVariance |  |
| wavelet-HHL | gldm | HighGrayLevelEmphasis |  |
| wavelet-HHL | gldm | LargeDependenceEmphasis |  |
| wavelet-HHL | gldm | LargeDependenceHighGrayLevelEmphasis |  |
| wavelet-HHL | gldm | LargeDependenceLowGrayLevelEmphasis |  |
| wavelet-HHL | gldm | LowGrayLevelEmphasis |  |
| wavelet-HHL | gldm | SmallDependenceEmphasis |  |
| wavelet-HHL | gldm | SmallDependenceHighGrayLevelEmphasis |  |
| wavelet-HHL | gldm | SmallDependenceLowGrayLevelEmphasis |  |
| wavelet-HHL | ngtdm | Busyness |  |
| wavelet-HHL | ngtdm | Coarseness |  |
| wavelet-HHL | ngtdm | Complexity |  |
| wavelet-HHL | ngtdm | Contrast |  |
| wavelet-HHL | ngtdm | Strength |  |
| wavelet-HHH | glcm | Autocorrelation |  |
| wavelet-HHH | glcm | ClusterProminence |  |
| wavelet-HHH | glcm | ClusterShade |  |
| wavelet-HHH | glcm | ClusterTendency |  |
| wavelet-HHH | glcm | Contrast |  |
| wavelet-HHH | glcm | Correlation |  |
| wavelet-HHH | glcm | DifferenceAverage |  |
| wavelet-HHH | glcm | DifferenceEntropy |  |
| wavelet-HHH | glcm | DifferenceVariance |  |
| wavelet-HHH | glcm | Id |  |
| wavelet-HHH | glcm | Idm |  |
| wavelet-HHH | glcm | Idmn |  |
| wavelet-HHH | glcm | Idn |  |
| wavelet-HHH | glcm | Imc1 |  |
| wavelet-HHH | glcm | Imc2 |  |
| wavelet-HHH | glcm | InverseVariance |  |
| wavelet-HHH | glcm | JointAverage |  |
| wavelet-HHH | glcm | JointEnergy |  |
| wavelet-HHH | glcm | JointEntropy |  |
| wavelet-HHH | glcm | MCC |  |
| wavelet-HHH | glcm | MaximumProbability |  |
| wavelet-HHH | glcm | SumAverage |  |
| wavelet-HHH | glcm | SumEntropy |  |
| wavelet-HHH | glcm | SumSquares |  |
| wavelet-HHH | glrlm | GrayLevelNonUniformity |  |
| wavelet-HHH | glrlm | GrayLevelNonUniformityNormalized |  |
| wavelet-HHH | glrlm | GrayLevelVariance |  |
| wavelet-HHH | glrlm | HighGrayLevelRunEmphasis |  |
| wavelet-HHH | glrlm | LongRunEmphasis |  |
| wavelet-HHH | glrlm | LongRunHighGrayLevelEmphasis |  |
| wavelet-HHH | glrlm | LongRunLowGrayLevelEmphasis |  |
| wavelet-HHH | glrlm | LowGrayLevelRunEmphasis |  |
| wavelet-HHH | glrlm | RunEntropy |  |
| wavelet-HHH | glrlm | RunLengthNonUniformity |  |
| wavelet-HHH | glrlm | RunLengthNonUniformityNormalized |  |
| wavelet-HHH | glrlm | RunPercentage |  |
| wavelet-HHH | glrlm | RunVariance |  |
| wavelet-HHH | glrlm | ShortRunEmphasis |  |
| wavelet-HHH | glrlm | ShortRunHighGrayLevelEmphasis |  |
| wavelet-HHH | glrlm | ShortRunLowGrayLevelEmphasis |  |
| wavelet-HHH | firstorder | 10Percentile |  |
| wavelet-HHH | firstorder | 90Percentile |  |
| wavelet-HHH | firstorder | Energy |  |
| wavelet-HHH | firstorder | Entropy |  |
| wavelet-HHH | firstorder | InterquartileRange |  |
| wavelet-HHH | firstorder | Kurtosis |  |
| wavelet-HHH | firstorder | Maximum |  |
| wavelet-HHH | firstorder | MeanAbsoluteDeviation |  |
| wavelet-HHH | firstorder | Mean |  |
| wavelet-HHH | firstorder | Median |  |
| wavelet-HHH | firstorder | Minimum |  |
| wavelet-HHH | firstorder | Range |  |
| wavelet-HHH | firstorder | RobustMeanAbsoluteDeviation |  |
| wavelet-HHH | firstorder | RootMeanSquared |  |
| wavelet-HHH | firstorder | Skewness |  |
| wavelet-HHH | firstorder | TotalEnergy |  |
| wavelet-HHH | firstorder | Uniformity |  |
| wavelet-HHH | firstorder | Variance |  |
| wavelet-HHH | glszm | GrayLevelNonUniformity |  |
| wavelet-HHH | glszm | GrayLevelNonUniformityNormalized |  |
| wavelet-HHH | glszm | GrayLevelVariance |  |
| wavelet-HHH | glszm | HighGrayLevelZoneEmphasis |  |
| wavelet-HHH | glszm | LargeAreaEmphasis |  |
| wavelet-HHH | glszm | LargeAreaHighGrayLevelEmphasis |  |
| wavelet-HHH | glszm | LargeAreaLowGrayLevelEmphasis |  |
| wavelet-HHH | glszm | LowGrayLevelZoneEmphasis |  |
| wavelet-HHH | glszm | SizeZoneNonUniformity |  |
| wavelet-HHH | glszm | SizeZoneNonUniformityNormalized |  |
| wavelet-HHH | glszm | SmallAreaEmphasis |  |
| wavelet-HHH | glszm | SmallAreaHighGrayLevelEmphasis |  |
| wavelet-HHH | glszm | SmallAreaLowGrayLevelEmphasis |  |
| wavelet-HHH | glszm | ZoneEntropy |  |
| wavelet-HHH | glszm | ZonePercentage |  |
| wavelet-HHH | glszm | ZoneVariance |  |
| wavelet-HHH | gldm | DependenceEntropy |  |
| wavelet-HHH | gldm | DependenceNonUniformity |  |
| wavelet-HHH | gldm | DependenceNonUniformityNormalized |  |
| wavelet-HHH | gldm | DependenceVariance |  |
| wavelet-HHH | gldm | GrayLevelNonUniformity |  |
| wavelet-HHH | gldm | GrayLevelVariance |  |
| wavelet-HHH | gldm | HighGrayLevelEmphasis |  |
| wavelet-HHH | gldm | LargeDependenceEmphasis |  |
| wavelet-HHH | gldm | LargeDependenceHighGrayLevelEmphasis |  |
| wavelet-HHH | gldm | LargeDependenceLowGrayLevelEmphasis |  |
| wavelet-HHH | gldm | LowGrayLevelEmphasis |  |
| wavelet-HHH | gldm | SmallDependenceEmphasis |  |
| wavelet-HHH | gldm | SmallDependenceHighGrayLevelEmphasis |  |
| wavelet-HHH | gldm | SmallDependenceLowGrayLevelEmphasis |  |
| wavelet-HHH | ngtdm | Busyness |  |
| wavelet-HHH | ngtdm | Coarseness |  |
| wavelet-HHH | ngtdm | Complexity |  |
| wavelet-HHH | ngtdm | Contrast |  |
| wavelet-HHH | ngtdm | Strength |  |
| wavelet-LLL | glcm | Autocorrelation |  |
| wavelet-LLL | glcm | ClusterProminence |  |
| wavelet-LLL | glcm | ClusterShade |  |
| wavelet-LLL | glcm | ClusterTendency |  |
| wavelet-LLL | glcm | Contrast |  |
| wavelet-LLL | glcm | Correlation |  |
| wavelet-LLL | glcm | DifferenceAverage |  |
| wavelet-LLL | glcm | DifferenceEntropy |  |
| wavelet-LLL | glcm | DifferenceVariance |  |
| wavelet-LLL | glcm | Id |  |
| wavelet-LLL | glcm | Idm |  |
| wavelet-LLL | glcm | Idmn |  |
| wavelet-LLL | glcm | Idn |  |
| wavelet-LLL | glcm | Imc1 |  |
| wavelet-LLL | glcm | Imc2 |  |
| wavelet-LLL | glcm | InverseVariance |  |
| wavelet-LLL | glcm | JointAverage |  |
| wavelet-LLL | glcm | JointEnergy |  |
| wavelet-LLL | glcm | JointEntropy |  |
| wavelet-LLL | glcm | MCC |  |
| wavelet-LLL | glcm | MaximumProbability |  |
| wavelet-LLL | glcm | SumAverage |  |
| wavelet-LLL | glcm | SumEntropy |  |
| wavelet-LLL | glcm | SumSquares |  |
| wavelet-LLL | glrlm | GrayLevelNonUniformity |  |
| wavelet-LLL | glrlm | GrayLevelNonUniformityNormalized |  |
| wavelet-LLL | glrlm | GrayLevelVariance |  |
| wavelet-LLL | glrlm | HighGrayLevelRunEmphasis |  |
| wavelet-LLL | glrlm | LongRunEmphasis |  |
| wavelet-LLL | glrlm | LongRunHighGrayLevelEmphasis |  |
| wavelet-LLL | glrlm | LongRunLowGrayLevelEmphasis |  |
| wavelet-LLL | glrlm | LowGrayLevelRunEmphasis |  |
| wavelet-LLL | glrlm | RunEntropy |  |
| wavelet-LLL | glrlm | RunLengthNonUniformity |  |
| wavelet-LLL | glrlm | RunLengthNonUniformityNormalized |  |
| wavelet-LLL | glrlm | RunPercentage |  |
| wavelet-LLL | glrlm | RunVariance |  |
| wavelet-LLL | glrlm | ShortRunEmphasis |  |
| wavelet-LLL | glrlm | ShortRunHighGrayLevelEmphasis |  |
| wavelet-LLL | glrlm | ShortRunLowGrayLevelEmphasis |  |
| wavelet-LLL | firstorder | 10Percentile |  |
| wavelet-LLL | firstorder | 90Percentile |  |
| wavelet-LLL | firstorder | Energy |  |
| wavelet-LLL | firstorder | Entropy |  |
| wavelet-LLL | firstorder | InterquartileRange |  |
| wavelet-LLL | firstorder | Kurtosis |  |
| wavelet-LLL | firstorder | Maximum |  |
| wavelet-LLL | firstorder | MeanAbsoluteDeviation |  |
| wavelet-LLL | firstorder | Mean |  |
| wavelet-LLL | firstorder | Median |  |
| wavelet-LLL | firstorder | Minimum |  |
| wavelet-LLL | firstorder | Range |  |
| wavelet-LLL | firstorder | RobustMeanAbsoluteDeviation |  |
| wavelet-LLL | firstorder | RootMeanSquared |  |
| wavelet-LLL | firstorder | Skewness |  |
| wavelet-LLL | firstorder | TotalEnergy |  |
| wavelet-LLL | firstorder | Uniformity |  |
| wavelet-LLL | firstorder | Variance |  |
| wavelet-LLL | glszm | GrayLevelNonUniformity |  |
| wavelet-LLL | glszm | GrayLevelNonUniformityNormalized |  |
| wavelet-LLL | glszm | GrayLevelVariance |  |
| wavelet-LLL | glszm | HighGrayLevelZoneEmphasis |  |
| wavelet-LLL | glszm | LargeAreaEmphasis |  |
| wavelet-LLL | glszm | LargeAreaHighGrayLevelEmphasis |  |
| wavelet-LLL | glszm | LargeAreaLowGrayLevelEmphasis |  |
| wavelet-LLL | glszm | LowGrayLevelZoneEmphasis |  |
| wavelet-LLL | glszm | SizeZoneNonUniformity |  |
| wavelet-LLL | glszm | SizeZoneNonUniformityNormalized |  |
| wavelet-LLL | glszm | SmallAreaEmphasis |  |
| wavelet-LLL | glszm | SmallAreaHighGrayLevelEmphasis |  |
| wavelet-LLL | glszm | SmallAreaLowGrayLevelEmphasis |  |
| wavelet-LLL | glszm | ZoneEntropy |  |
| wavelet-LLL | glszm | ZonePercentage |  |
| wavelet-LLL | glszm | ZoneVariance |  |
| wavelet-LLL | gldm | DependenceEntropy |  |
| wavelet-LLL | gldm | DependenceNonUniformity |  |
| wavelet-LLL | gldm | DependenceNonUniformityNormalized |  |
| wavelet-LLL | gldm | DependenceVariance |  |
| wavelet-LLL | gldm | GrayLevelNonUniformity |  |
| wavelet-LLL | gldm | GrayLevelVariance |  |
| wavelet-LLL | gldm | HighGrayLevelEmphasis |  |
| wavelet-LLL | gldm | LargeDependenceEmphasis |  |
| wavelet-LLL | gldm | LargeDependenceHighGrayLevelEmphasis |  |
| wavelet-LLL | gldm | LargeDependenceLowGrayLevelEmphasis |  |
| wavelet-LLL | gldm | LowGrayLevelEmphasis |  |
| wavelet-LLL | gldm | SmallDependenceEmphasis |  |
| wavelet-LLL | gldm | SmallDependenceHighGrayLevelEmphasis |  |
| wavelet-LLL | gldm | SmallDependenceLowGrayLevelEmphasis |  |
| wavelet-LLL | ngtdm | Busyness |  |
| wavelet-LLL | ngtdm | Coarseness |  |
| wavelet-LLL | ngtdm | Complexity |  |
| wavelet-LLL | ngtdm | Contrast |  |
| wavelet-LLL | ngtdm | Strength |  |
| square | glrlm | GrayLevelNonUniformity |  |
| square | glrlm | LongRunEmphasis |  |
| square | glrlm | LongRunHighGrayLevelEmphasis |  |
| square | glrlm | LongRunLowGrayLevelEmphasis |  |
| square | glrlm | RunEntropy |  |
| square | glrlm | RunLengthNonUniformity |  |
| square | glrlm | RunLengthNonUniformityNormalized |  |
| square | glrlm | RunPercentage |  |
| square | glrlm | RunVariance |  |
| square | glrlm | ShortRunEmphasis |  |
| square | glrlm | ShortRunHighGrayLevelEmphasis |  |
| square | glrlm | ShortRunLowGrayLevelEmphasis |  |
| square | firstorder | 10Percentile |  |
| square | firstorder | 90Percentile |  |
| square | firstorder | Energy |  |
| square | firstorder | InterquartileRange |  |
| square | firstorder | Kurtosis |  |
| square | firstorder | Maximum |  |
| square | firstorder | MeanAbsoluteDeviation |  |
| square | firstorder | Mean |  |
| square | firstorder | Median |  |
| square | firstorder | Minimum |  |
| square | firstorder | Range |  |
| square | firstorder | RobustMeanAbsoluteDeviation |  |
| square | firstorder | RootMeanSquared |  |
| square | firstorder | Skewness |  |
| square | firstorder | TotalEnergy |  |
| square | firstorder | Variance |  |
| square | glszm | GrayLevelNonUniformity |  |
| square | glszm | LargeAreaEmphasis |  |
| square | glszm | LargeAreaHighGrayLevelEmphasis |  |
| square | glszm | LargeAreaLowGrayLevelEmphasis |  |
| square | glszm | SizeZoneNonUniformity |  |
| square | glszm | SizeZoneNonUniformityNormalized |  |
| square | glszm | SmallAreaEmphasis |  |
| square | glszm | SmallAreaHighGrayLevelEmphasis |  |
| square | glszm | SmallAreaLowGrayLevelEmphasis |  |
| square | glszm | ZoneEntropy |  |
| square | glszm | ZonePercentage |  |
| square | glszm | ZoneVariance |  |
| square | gldm | DependenceEntropy |  |
| square | gldm | DependenceNonUniformity |  |
| square | gldm | DependenceNonUniformityNormalized |  |
| square | gldm | DependenceVariance |  |
| square | gldm | GrayLevelNonUniformity |  |
| square | gldm | LargeDependenceEmphasis |  |
| square | gldm | LargeDependenceHighGrayLevelEmphasis |  |
| square | gldm | LargeDependenceLowGrayLevelEmphasis |  |
| square | gldm | SmallDependenceEmphasis |  |
| square | gldm | SmallDependenceHighGrayLevelEmphasis |  |
| square | gldm | SmallDependenceLowGrayLevelEmphasis |  |
| squareroot | glcm | Autocorrelation |  |
| squareroot | glcm | ClusterProminence |  |
| squareroot | glcm | ClusterShade |  |
| squareroot | glcm | ClusterTendency |  |
| squareroot | glcm | Contrast |  |
| squareroot | glcm | Correlation |  |
| squareroot | glcm | DifferenceAverage |  |
| squareroot | glcm | DifferenceEntropy |  |
| squareroot | glcm | DifferenceVariance |  |
| squareroot | glcm | Id |  |
| squareroot | glcm | Idm |  |
| squareroot | glcm | Idmn |  |
| squareroot | glcm | Idn |  |
| squareroot | glcm | Imc1 |  |
| squareroot | glcm | Imc2 |  |
| squareroot | glcm | InverseVariance |  |
| squareroot | glcm | JointAverage |  |
| squareroot | glcm | JointEnergy |  |
| squareroot | glcm | JointEntropy |  |
| squareroot | glcm | MCC |  |
| squareroot | glcm | MaximumProbability |  |
| squareroot | glcm | SumAverage |  |
| squareroot | glcm | SumEntropy |  |
| squareroot | glcm | SumSquares |  |
| squareroot | glrlm | GrayLevelNonUniformity |  |
| squareroot | glrlm | GrayLevelNonUniformityNormalized |  |
| squareroot | glrlm | GrayLevelVariance |  |
| squareroot | glrlm | HighGrayLevelRunEmphasis |  |
| squareroot | glrlm | LongRunEmphasis |  |
| squareroot | glrlm | LongRunHighGrayLevelEmphasis |  |
| squareroot | glrlm | LongRunLowGrayLevelEmphasis |  |
| squareroot | glrlm | LowGrayLevelRunEmphasis |  |
| squareroot | glrlm | RunEntropy |  |
| squareroot | glrlm | RunLengthNonUniformity |  |
| squareroot | glrlm | RunLengthNonUniformityNormalized |  |
| squareroot | glrlm | RunPercentage |  |
| squareroot | glrlm | RunVariance |  |
| squareroot | glrlm | ShortRunEmphasis |  |
| squareroot | glrlm | ShortRunHighGrayLevelEmphasis |  |
| squareroot | glrlm | ShortRunLowGrayLevelEmphasis |  |
| squareroot | firstorder | 10Percentile |  |
| squareroot | firstorder | 90Percentile |  |
| squareroot | firstorder | Energy |  |
| squareroot | firstorder | Entropy |  |
| squareroot | firstorder | InterquartileRange |  |
| squareroot | firstorder | Kurtosis |  |
| squareroot | firstorder | Maximum |  |
| squareroot | firstorder | MeanAbsoluteDeviation |  |
| squareroot | firstorder | Mean |  |
| squareroot | firstorder | Median |  |
| squareroot | firstorder | Minimum |  |
| squareroot | firstorder | Range |  |
| squareroot | firstorder | RobustMeanAbsoluteDeviation |  |
| squareroot | firstorder | RootMeanSquared |  |
| squareroot | firstorder | Skewness |  |
| squareroot | firstorder | TotalEnergy |  |
| squareroot | firstorder | Uniformity |  |
| squareroot | firstorder | Variance |  |
| squareroot | glszm | GrayLevelNonUniformity |  |
| squareroot | glszm | GrayLevelNonUniformityNormalized |  |
| squareroot | glszm | GrayLevelVariance |  |
| squareroot | glszm | HighGrayLevelZoneEmphasis |  |
| squareroot | glszm | LargeAreaEmphasis |  |
| squareroot | glszm | LargeAreaHighGrayLevelEmphasis |  |
| squareroot | glszm | LargeAreaLowGrayLevelEmphasis |  |
| squareroot | glszm | LowGrayLevelZoneEmphasis |  |
| squareroot | glszm | SizeZoneNonUniformity |  |
| squareroot | glszm | SizeZoneNonUniformityNormalized |  |
| squareroot | glszm | SmallAreaEmphasis |  |
| squareroot | glszm | SmallAreaHighGrayLevelEmphasis |  |
| squareroot | glszm | SmallAreaLowGrayLevelEmphasis |  |
| squareroot | glszm | ZoneEntropy |  |
| squareroot | glszm | ZonePercentage |  |
| squareroot | glszm | ZoneVariance |  |
| squareroot | gldm | DependenceEntropy |  |
| squareroot | gldm | DependenceNonUniformity |  |
| squareroot | gldm | DependenceNonUniformityNormalized |  |
| squareroot | gldm | DependenceVariance |  |
| squareroot | gldm | GrayLevelNonUniformity |  |
| squareroot | gldm | GrayLevelVariance |  |
| squareroot | gldm | HighGrayLevelEmphasis |  |
| squareroot | gldm | LargeDependenceEmphasis |  |
| squareroot | gldm | LargeDependenceHighGrayLevelEmphasis |  |
| squareroot | gldm | LargeDependenceLowGrayLevelEmphasis |  |
| squareroot | gldm | LowGrayLevelEmphasis |  |
| squareroot | gldm | SmallDependenceEmphasis |  |
| squareroot | gldm | SmallDependenceHighGrayLevelEmphasis |  |
| squareroot | gldm | SmallDependenceLowGrayLevelEmphasis |  |
| squareroot | ngtdm | Busyness |  |
| squareroot | ngtdm | Coarseness |  |
| squareroot | ngtdm | Complexity |  |
| squareroot | ngtdm | Contrast |  |
| squareroot | ngtdm | Strength |  |
| logarithm | glcm | Autocorrelation |  |
| logarithm | glcm | ClusterProminence |  |
| logarithm | glcm | ClusterShade |  |
| logarithm | glcm | ClusterTendency |  |
| logarithm | glcm | Contrast |  |
| logarithm | glcm | Correlation |  |
| logarithm | glcm | DifferenceAverage |  |
| logarithm | glcm | DifferenceEntropy |  |
| logarithm | glcm | DifferenceVariance |  |
| logarithm | glcm | Id |  |
| logarithm | glcm | Idm |  |
| logarithm | glcm | Idmn |  |
| logarithm | glcm | Idn |  |
| logarithm | glcm | Imc1 |  |
| logarithm | glcm | Imc2 |  |
| logarithm | glcm | InverseVariance |  |
| logarithm | glcm | JointAverage |  |
| logarithm | glcm | JointEnergy |  |
| logarithm | glcm | JointEntropy |  |
| logarithm | glcm | MCC |  |
| logarithm | glcm | MaximumProbability |  |
| logarithm | glcm | SumAverage |  |
| logarithm | glcm | SumEntropy |  |
| logarithm | glcm | SumSquares |  |
| logarithm | glrlm | GrayLevelNonUniformity |  |
| logarithm | glrlm | GrayLevelNonUniformityNormalized |  |
| logarithm | glrlm | GrayLevelVariance |  |
| logarithm | glrlm | HighGrayLevelRunEmphasis |  |
| logarithm | glrlm | LongRunEmphasis |  |
| logarithm | glrlm | LongRunHighGrayLevelEmphasis |  |
| logarithm | glrlm | LongRunLowGrayLevelEmphasis |  |
| logarithm | glrlm | LowGrayLevelRunEmphasis |  |
| logarithm | glrlm | RunEntropy |  |
| logarithm | glrlm | RunLengthNonUniformity |  |
| logarithm | glrlm | RunLengthNonUniformityNormalized |  |
| logarithm | glrlm | RunPercentage |  |
| logarithm | glrlm | RunVariance |  |
| logarithm | glrlm | ShortRunEmphasis |  |
| logarithm | glrlm | ShortRunHighGrayLevelEmphasis |  |
| logarithm | glrlm | ShortRunLowGrayLevelEmphasis |  |
| logarithm | firstorder | 10Percentile |  |
| logarithm | firstorder | 90Percentile |  |
| logarithm | firstorder | Energy |  |
| logarithm | firstorder | Entropy |  |
| logarithm | firstorder | InterquartileRange |  |
| logarithm | firstorder | Kurtosis |  |
| logarithm | firstorder | Maximum |  |
| logarithm | firstorder | MeanAbsoluteDeviation |  |
| logarithm | firstorder | Mean |  |
| logarithm | firstorder | Median |  |
| logarithm | firstorder | Minimum |  |
| logarithm | firstorder | Range |  |
| logarithm | firstorder | RobustMeanAbsoluteDeviation |  |
| logarithm | firstorder | RootMeanSquared |  |
| logarithm | firstorder | Skewness |  |
| logarithm | firstorder | TotalEnergy |  |
| logarithm | firstorder | Uniformity |  |
| logarithm | firstorder | Variance |  |
| logarithm | glszm | GrayLevelNonUniformity |  |
| logarithm | glszm | GrayLevelNonUniformityNormalized |  |
| logarithm | glszm | GrayLevelVariance |  |
| logarithm | glszm | HighGrayLevelZoneEmphasis |  |
| logarithm | glszm | LargeAreaEmphasis |  |
| logarithm | glszm | LargeAreaHighGrayLevelEmphasis |  |
| logarithm | glszm | LargeAreaLowGrayLevelEmphasis |  |
| logarithm | glszm | LowGrayLevelZoneEmphasis |  |
| logarithm | glszm | SizeZoneNonUniformity |  |
| logarithm | glszm | SizeZoneNonUniformityNormalized |  |
| logarithm | glszm | SmallAreaEmphasis |  |
| logarithm | glszm | SmallAreaHighGrayLevelEmphasis |  |
| logarithm | glszm | SmallAreaLowGrayLevelEmphasis |  |
| logarithm | glszm | ZoneEntropy |  |
| logarithm | glszm | ZonePercentage |  |
| logarithm | glszm | ZoneVariance |  |
| logarithm | gldm | DependenceEntropy |  |
| logarithm | gldm | DependenceNonUniformity |  |
| logarithm | gldm | DependenceNonUniformityNormalized |  |
| logarithm | gldm | DependenceVariance |  |
| logarithm | gldm | GrayLevelNonUniformity |  |
| logarithm | gldm | GrayLevelVariance |  |
| logarithm | gldm | HighGrayLevelEmphasis |  |
| logarithm | gldm | LargeDependenceEmphasis |  |
| logarithm | gldm | LargeDependenceHighGrayLevelEmphasis |  |
| logarithm | gldm | LargeDependenceLowGrayLevelEmphasis |  |
| logarithm | gldm | LowGrayLevelEmphasis |  |
| logarithm | gldm | SmallDependenceEmphasis |  |
| logarithm | gldm | SmallDependenceHighGrayLevelEmphasis |  |
| logarithm | gldm | SmallDependenceLowGrayLevelEmphasis |  |
| logarithm | ngtdm | Busyness |  |
| logarithm | ngtdm | Coarseness |  |
| logarithm | ngtdm | Complexity |  |
| logarithm | ngtdm | Contrast |  |
| logarithm | ngtdm | Strength |  |
| exponential | glrlm | GrayLevelNonUniformity |  |
| exponential | glrlm | LongRunEmphasis |  |
| exponential | glrlm | LongRunHighGrayLevelEmphasis |  |
| exponential | glrlm | LongRunLowGrayLevelEmphasis |  |
| exponential | glrlm | RunEntropy |  |
| exponential | glrlm | RunLengthNonUniformity |  |
| exponential | glrlm | RunLengthNonUniformityNormalized |  |
| exponential | glrlm | RunPercentage |  |
| exponential | glrlm | RunVariance |  |
| exponential | glrlm | ShortRunEmphasis |  |
| exponential | glrlm | ShortRunHighGrayLevelEmphasis |  |
| exponential | glrlm | ShortRunLowGrayLevelEmphasis |  |
| exponential | firstorder | 10Percentile |  |
| exponential | firstorder | 90Percentile |  |
| exponential | firstorder | Energy |  |
| exponential | firstorder | InterquartileRange |  |
| exponential | firstorder | Kurtosis |  |
| exponential | firstorder | Maximum |  |
| exponential | firstorder | MeanAbsoluteDeviation |  |
| exponential | firstorder | Mean |  |
| exponential | firstorder | Median |  |
| exponential | firstorder | Minimum |  |
| exponential | firstorder | Range |  |
| exponential | firstorder | RobustMeanAbsoluteDeviation |  |
| exponential | firstorder | RootMeanSquared |  |
| exponential | firstorder | Skewness |  |
| exponential | firstorder | TotalEnergy |  |
| exponential | firstorder | Variance |  |
| exponential | glszm | GrayLevelNonUniformity |  |
| exponential | glszm | LargeAreaEmphasis |  |
| exponential | glszm | LargeAreaHighGrayLevelEmphasis |  |
| exponential | glszm | LargeAreaLowGrayLevelEmphasis |  |
| exponential | glszm | SizeZoneNonUniformity |  |
| exponential | glszm | SizeZoneNonUniformityNormalized |  |
| exponential | glszm | SmallAreaEmphasis |  |
| exponential | glszm | SmallAreaHighGrayLevelEmphasis |  |
| exponential | glszm | SmallAreaLowGrayLevelEmphasis |  |
| exponential | glszm | ZoneEntropy |  |
| exponential | glszm | ZonePercentage |  |
| exponential | glszm | ZoneVariance |  |
| exponential | gldm | DependenceEntropy |  |
| exponential | gldm | DependenceNonUniformity |  |
| exponential | gldm | DependenceNonUniformityNormalized |  |
| exponential | gldm | DependenceVariance |  |
| exponential | gldm | GrayLevelNonUniformity |  |
| exponential | gldm | LargeDependenceEmphasis |  |
| exponential | gldm | LargeDependenceHighGrayLevelEmphasis |  |
| exponential | gldm | LargeDependenceLowGrayLevelEmphasis |  |
| exponential | gldm | SmallDependenceEmphasis |  |
| exponential | gldm | SmallDependenceHighGrayLevelEmphasis |  |
| exponential | gldm | SmallDependenceLowGrayLevelEmphasis |  |
| gradient | glrlm | GrayLevelNonUniformity |  |
| gradient | glrlm | LongRunEmphasis |  |
| gradient | glrlm | LongRunHighGrayLevelEmphasis |  |
| gradient | glrlm | LongRunLowGrayLevelEmphasis |  |
| gradient | glrlm | RunEntropy |  |
| gradient | glrlm | RunLengthNonUniformity |  |
| gradient | glrlm | RunLengthNonUniformityNormalized |  |
| gradient | glrlm | RunPercentage |  |
| gradient | glrlm | RunVariance |  |
| gradient | glrlm | ShortRunEmphasis |  |
| gradient | glrlm | ShortRunHighGrayLevelEmphasis |  |
| gradient | glrlm | ShortRunLowGrayLevelEmphasis |  |
| gradient | firstorder | 10Percentile |  |
| gradient | firstorder | 90Percentile |  |
| gradient | firstorder | Energy |  |
| gradient | firstorder | InterquartileRange |  |
| gradient | firstorder | Kurtosis |  |
| gradient | firstorder | Maximum |  |
| gradient | firstorder | MeanAbsoluteDeviation |  |
| gradient | firstorder | Mean |  |
| gradient | firstorder | Median |  |
| gradient | firstorder | Minimum |  |
| gradient | firstorder | Range |  |
| gradient | firstorder | RobustMeanAbsoluteDeviation |  |
| gradient | firstorder | RootMeanSquared |  |
| gradient | firstorder | Skewness |  |
| gradient | firstorder | TotalEnergy |  |
| gradient | firstorder | Variance |  |
| gradient | glszm | GrayLevelNonUniformity |  |
| gradient | glszm | LargeAreaEmphasis |  |
| gradient | glszm | LargeAreaHighGrayLevelEmphasis |  |
| gradient | glszm | LargeAreaLowGrayLevelEmphasis |  |
| gradient | glszm | SizeZoneNonUniformity |  |
| gradient | glszm | SizeZoneNonUniformityNormalized |  |
| gradient | glszm | SmallAreaEmphasis |  |
| gradient | glszm | SmallAreaHighGrayLevelEmphasis |  |
| gradient | glszm | SmallAreaLowGrayLevelEmphasis |  |
| gradient | glszm | ZoneEntropy |  |
| gradient | glszm | ZonePercentage |  |
| gradient | glszm | ZoneVariance |  |
| gradient | gldm | DependenceEntropy |  |
| gradient | gldm | DependenceNonUniformity |  |
| gradient | gldm | DependenceNonUniformityNormalized |  |
| gradient | gldm | DependenceVariance |  |
| gradient | gldm | GrayLevelNonUniformity |  |
| gradient | gldm | LargeDependenceEmphasis |  |
| gradient | gldm | LargeDependenceHighGrayLevelEmphasis |  |
| gradient | gldm | LargeDependenceLowGrayLevelEmphasis |  |
| gradient | gldm | SmallDependenceEmphasis |  |
| gradient | gldm | SmallDependenceHighGrayLevelEmphasis |  |
| gradient | gldm | SmallDependenceLowGrayLevelEmphasis |  |
| lbp-2D | glrlm | GrayLevelNonUniformity |  |
| lbp-2D | glrlm | LongRunEmphasis |  |
| lbp-2D | glrlm | LongRunHighGrayLevelEmphasis |  |
| lbp-2D | glrlm | LongRunLowGrayLevelEmphasis |  |
| lbp-2D | glrlm | RunEntropy |  |
| lbp-2D | glrlm | RunLengthNonUniformity |  |
| lbp-2D | glrlm | RunLengthNonUniformityNormalized |  |
| lbp-2D | glrlm | RunPercentage |  |
| lbp-2D | glrlm | RunVariance |  |
| lbp-2D | glrlm | ShortRunEmphasis |  |
| lbp-2D | glrlm | ShortRunHighGrayLevelEmphasis |  |
| lbp-2D | glrlm | ShortRunLowGrayLevelEmphasis |  |
| lbp-2D | firstorder | 10Percentile |  |
| lbp-2D | firstorder | Energy |  |
| lbp-2D | firstorder | InterquartileRange |  |
| lbp-2D | firstorder | Kurtosis |  |
| lbp-2D | firstorder | MeanAbsoluteDeviation |  |
| lbp-2D | firstorder | Mean |  |
| lbp-2D | firstorder | Median |  |
| lbp-2D | firstorder | RobustMeanAbsoluteDeviation |  |
| lbp-2D | firstorder | RootMeanSquared |  |
| lbp-2D | firstorder | Skewness |  |
| lbp-2D | firstorder | TotalEnergy |  |
| lbp-2D | firstorder | Variance |  |
| lbp-2D | glszm | GrayLevelNonUniformity |  |
| lbp-2D | glszm | LargeAreaEmphasis |  |
| lbp-2D | glszm | LargeAreaHighGrayLevelEmphasis |  |
| lbp-2D | glszm | LargeAreaLowGrayLevelEmphasis |  |
| lbp-2D | glszm | SizeZoneNonUniformity |  |
| lbp-2D | glszm | SizeZoneNonUniformityNormalized |  |
| lbp-2D | glszm | SmallAreaEmphasis |  |
| lbp-2D | glszm | SmallAreaHighGrayLevelEmphasis |  |
| lbp-2D | glszm | SmallAreaLowGrayLevelEmphasis |  |
| lbp-2D | glszm | ZoneEntropy |  |
| lbp-2D | glszm | ZonePercentage |  |
| lbp-2D | glszm | ZoneVariance |  |
| lbp-2D | gldm | DependenceEntropy |  |
| lbp-2D | gldm | DependenceNonUniformity |  |
| lbp-2D | gldm | DependenceNonUniformityNormalized |  |
| lbp-2D | gldm | DependenceVariance |  |
| lbp-2D | gldm | GrayLevelNonUniformity |  |
| lbp-2D | gldm | LargeDependenceEmphasis |  |
| lbp-2D | gldm | LargeDependenceHighGrayLevelEmphasis |  |
| lbp-2D | gldm | LargeDependenceLowGrayLevelEmphasis |  |
| lbp-2D | gldm | SmallDependenceEmphasis |  |
| lbp-2D | gldm | SmallDependenceHighGrayLevelEmphasis |  |
| lbp-2D | gldm | SmallDependenceLowGrayLevelEmphasis |  |
| lbp-3D-m1 | glrlm | GrayLevelNonUniformity |  |
| lbp-3D-m1 | glrlm | LongRunEmphasis |  |
| lbp-3D-m1 | glrlm | LongRunHighGrayLevelEmphasis |  |
| lbp-3D-m1 | glrlm | LongRunLowGrayLevelEmphasis |  |
| lbp-3D-m1 | glrlm | RunEntropy |  |
| lbp-3D-m1 | glrlm | RunLengthNonUniformity |  |
| lbp-3D-m1 | glrlm | RunLengthNonUniformityNormalized |  |
| lbp-3D-m1 | glrlm | RunPercentage |  |
| lbp-3D-m1 | glrlm | RunVariance |  |
| lbp-3D-m1 | glrlm | ShortRunEmphasis |  |
| lbp-3D-m1 | glrlm | ShortRunHighGrayLevelEmphasis |  |
| lbp-3D-m1 | glrlm | ShortRunLowGrayLevelEmphasis |  |
| lbp-3D-m1 | firstorder | 10Percentile |  |
| lbp-3D-m1 | firstorder | 90Percentile |  |
| lbp-3D-m1 | firstorder | Energy |  |
| lbp-3D-m1 | firstorder | InterquartileRange |  |
| lbp-3D-m1 | firstorder | Kurtosis |  |
| lbp-3D-m1 | firstorder | MeanAbsoluteDeviation |  |
| lbp-3D-m1 | firstorder | Mean |  |
| lbp-3D-m1 | firstorder | Median |  |
| lbp-3D-m1 | firstorder | RobustMeanAbsoluteDeviation |  |
| lbp-3D-m1 | firstorder | RootMeanSquared |  |
| lbp-3D-m1 | firstorder | Skewness |  |
| lbp-3D-m1 | firstorder | TotalEnergy |  |
| lbp-3D-m1 | firstorder | Variance |  |
| lbp-3D-m1 | glszm | GrayLevelNonUniformity |  |
| lbp-3D-m1 | glszm | LargeAreaEmphasis |  |
| lbp-3D-m1 | glszm | LargeAreaHighGrayLevelEmphasis |  |
| lbp-3D-m1 | glszm | LargeAreaLowGrayLevelEmphasis |  |
| lbp-3D-m1 | glszm | SizeZoneNonUniformity |  |
| lbp-3D-m1 | glszm | SizeZoneNonUniformityNormalized |  |
| lbp-3D-m1 | glszm | SmallAreaEmphasis |  |
| lbp-3D-m1 | glszm | SmallAreaHighGrayLevelEmphasis |  |
| lbp-3D-m1 | glszm | SmallAreaLowGrayLevelEmphasis |  |
| lbp-3D-m1 | glszm | ZoneEntropy |  |
| lbp-3D-m1 | glszm | ZonePercentage |  |
| lbp-3D-m1 | glszm | ZoneVariance |  |
| lbp-3D-m1 | gldm | DependenceEntropy |  |
| lbp-3D-m1 | gldm | DependenceNonUniformity |  |
| lbp-3D-m1 | gldm | DependenceNonUniformityNormalized |  |
| lbp-3D-m1 | gldm | DependenceVariance |  |
| lbp-3D-m1 | gldm | GrayLevelNonUniformity |  |
| lbp-3D-m1 | gldm | LargeDependenceEmphasis |  |
| lbp-3D-m1 | gldm | LargeDependenceHighGrayLevelEmphasis |  |
| lbp-3D-m1 | gldm | LargeDependenceLowGrayLevelEmphasis |  |
| lbp-3D-m1 | gldm | SmallDependenceEmphasis |  |
| lbp-3D-m1 | gldm | SmallDependenceHighGrayLevelEmphasis |  |
| lbp-3D-m1 | gldm | SmallDependenceLowGrayLevelEmphasis |  |
| lbp-3D-m2 | glrlm | GrayLevelNonUniformity |  |
| lbp-3D-m2 | glrlm | LongRunEmphasis |  |
| lbp-3D-m2 | glrlm | LongRunHighGrayLevelEmphasis |  |
| lbp-3D-m2 | glrlm | LongRunLowGrayLevelEmphasis |  |
| lbp-3D-m2 | glrlm | RunEntropy |  |
| lbp-3D-m2 | glrlm | RunLengthNonUniformity |  |
| lbp-3D-m2 | glrlm | RunLengthNonUniformityNormalized |  |
| lbp-3D-m2 | glrlm | RunPercentage |  |
| lbp-3D-m2 | glrlm | RunVariance |  |
| lbp-3D-m2 | glrlm | ShortRunEmphasis |  |
| lbp-3D-m2 | glrlm | ShortRunHighGrayLevelEmphasis |  |
| lbp-3D-m2 | glrlm | ShortRunLowGrayLevelEmphasis |  |
| lbp-3D-m2 | firstorder | 10Percentile |  |
| lbp-3D-m2 | firstorder | 90Percentile |  |
| lbp-3D-m2 | firstorder | Energy |  |
| lbp-3D-m2 | firstorder | InterquartileRange |  |
| lbp-3D-m2 | firstorder | Kurtosis |  |
| lbp-3D-m2 | firstorder | Maximum |  |
| lbp-3D-m2 | firstorder | MeanAbsoluteDeviation |  |
| lbp-3D-m2 | firstorder | Mean |  |
| lbp-3D-m2 | firstorder | Median |  |
| lbp-3D-m2 | firstorder | Range |  |
| lbp-3D-m2 | firstorder | RobustMeanAbsoluteDeviation |  |
| lbp-3D-m2 | firstorder | RootMeanSquared |  |
| lbp-3D-m2 | firstorder | Skewness |  |
| lbp-3D-m2 | firstorder | TotalEnergy |  |
| lbp-3D-m2 | firstorder | Variance |  |
| lbp-3D-m2 | glszm | GrayLevelNonUniformity |  |
| lbp-3D-m2 | glszm | LargeAreaEmphasis |  |
| lbp-3D-m2 | glszm | LargeAreaHighGrayLevelEmphasis |  |
| lbp-3D-m2 | glszm | LargeAreaLowGrayLevelEmphasis |  |
| lbp-3D-m2 | glszm | SizeZoneNonUniformity |  |
| lbp-3D-m2 | glszm | SizeZoneNonUniformityNormalized |  |
| lbp-3D-m2 | glszm | SmallAreaEmphasis |  |
| lbp-3D-m2 | glszm | SmallAreaHighGrayLevelEmphasis |  |
| lbp-3D-m2 | glszm | SmallAreaLowGrayLevelEmphasis |  |
| lbp-3D-m2 | glszm | ZoneEntropy |  |
| lbp-3D-m2 | glszm | ZonePercentage |  |
| lbp-3D-m2 | glszm | ZoneVariance |  |
| lbp-3D-m2 | gldm | DependenceEntropy |  |
| lbp-3D-m2 | gldm | DependenceNonUniformity |  |
| lbp-3D-m2 | gldm | DependenceNonUniformityNormalized |  |
| lbp-3D-m2 | gldm | DependenceVariance |  |
| lbp-3D-m2 | gldm | GrayLevelNonUniformity |  |
| lbp-3D-m2 | gldm | LargeDependenceEmphasis |  |
| lbp-3D-m2 | gldm | LargeDependenceHighGrayLevelEmphasis |  |
| lbp-3D-m2 | gldm | LargeDependenceLowGrayLevelEmphasis |  |
| lbp-3D-m2 | gldm | SmallDependenceEmphasis |  |
| lbp-3D-m2 | gldm | SmallDependenceHighGrayLevelEmphasis |  |
| lbp-3D-m2 | gldm | SmallDependenceLowGrayLevelEmphasis |  |
| lbp-3D-k | glcm | Autocorrelation |  |
| lbp-3D-k | glcm | ClusterProminence |  |
| lbp-3D-k | glcm | ClusterShade |  |
| lbp-3D-k | glcm | ClusterTendency |  |
| lbp-3D-k | glcm | Contrast |  |
| lbp-3D-k | glcm | Correlation |  |
| lbp-3D-k | glcm | DifferenceAverage |  |
| lbp-3D-k | glcm | DifferenceEntropy |  |
| lbp-3D-k | glcm | DifferenceVariance |  |
| lbp-3D-k | glcm | Id |  |
| lbp-3D-k | glcm | Idm |  |
| lbp-3D-k | glcm | Idmn |  |
| lbp-3D-k | glcm | Idn |  |
| lbp-3D-k | glcm | Imc1 |  |
| lbp-3D-k | glcm | Imc2 |  |
| lbp-3D-k | glcm | InverseVariance |  |
| lbp-3D-k | glcm | JointAverage |  |
| lbp-3D-k | glcm | JointEnergy |  |
| lbp-3D-k | glcm | JointEntropy |  |
| lbp-3D-k | glcm | MCC |  |
| lbp-3D-k | glcm | MaximumProbability |  |
| lbp-3D-k | glcm | SumAverage |  |
| lbp-3D-k | glcm | SumEntropy |  |
| lbp-3D-k | glcm | SumSquares |  |
| lbp-3D-k | glrlm | GrayLevelNonUniformity |  |
| lbp-3D-k | glrlm | GrayLevelNonUniformityNormalized |  |
| lbp-3D-k | glrlm | GrayLevelVariance |  |
| lbp-3D-k | glrlm | HighGrayLevelRunEmphasis |  |
| lbp-3D-k | glrlm | LongRunEmphasis |  |
| lbp-3D-k | glrlm | LongRunHighGrayLevelEmphasis |  |
| lbp-3D-k | glrlm | LongRunLowGrayLevelEmphasis |  |
| lbp-3D-k | glrlm | LowGrayLevelRunEmphasis |  |
| lbp-3D-k | glrlm | RunEntropy |  |
| lbp-3D-k | glrlm | RunLengthNonUniformity |  |
| lbp-3D-k | glrlm | RunLengthNonUniformityNormalized |  |
| lbp-3D-k | glrlm | RunPercentage |  |
| lbp-3D-k | glrlm | RunVariance |  |
| lbp-3D-k | glrlm | ShortRunEmphasis |  |
| lbp-3D-k | glrlm | ShortRunHighGrayLevelEmphasis |  |
| lbp-3D-k | glrlm | ShortRunLowGrayLevelEmphasis |  |
| lbp-3D-k | firstorder | 10Percentile |  |
| lbp-3D-k | firstorder | 90Percentile |  |
| lbp-3D-k | firstorder | Energy |  |
| lbp-3D-k | firstorder | Entropy |  |
| lbp-3D-k | firstorder | InterquartileRange |  |
| lbp-3D-k | firstorder | Kurtosis |  |
| lbp-3D-k | firstorder | Maximum |  |
| lbp-3D-k | firstorder | MeanAbsoluteDeviation |  |
| lbp-3D-k | firstorder | Mean |  |
| lbp-3D-k | firstorder | Median |  |
| lbp-3D-k | firstorder | Minimum |  |
| lbp-3D-k | firstorder | Range |  |
| lbp-3D-k | firstorder | RobustMeanAbsoluteDeviation |  |
| lbp-3D-k | firstorder | RootMeanSquared |  |
| lbp-3D-k | firstorder | Skewness |  |
| lbp-3D-k | firstorder | TotalEnergy |  |
| lbp-3D-k | firstorder | Uniformity |  |
| lbp-3D-k | firstorder | Variance |  |
| lbp-3D-k | glszm | GrayLevelNonUniformity |  |
| lbp-3D-k | glszm | GrayLevelNonUniformityNormalized |  |
| lbp-3D-k | glszm | GrayLevelVariance |  |
| lbp-3D-k | glszm | HighGrayLevelZoneEmphasis |  |
| lbp-3D-k | glszm | LargeAreaEmphasis |  |
| lbp-3D-k | glszm | LargeAreaHighGrayLevelEmphasis |  |
| lbp-3D-k | glszm | LargeAreaLowGrayLevelEmphasis |  |
| lbp-3D-k | glszm | LowGrayLevelZoneEmphasis |  |
| lbp-3D-k | glszm | SizeZoneNonUniformity |  |
| lbp-3D-k | glszm | SizeZoneNonUniformityNormalized |  |
| lbp-3D-k | glszm | SmallAreaEmphasis |  |
| lbp-3D-k | glszm | SmallAreaHighGrayLevelEmphasis |  |
| lbp-3D-k | glszm | SmallAreaLowGrayLevelEmphasis |  |
| lbp-3D-k | glszm | ZoneEntropy |  |
| lbp-3D-k | glszm | ZonePercentage |  |
| lbp-3D-k | glszm | ZoneVariance |  |
| lbp-3D-k | gldm | DependenceEntropy |  |
| lbp-3D-k | gldm | DependenceNonUniformity |  |
| lbp-3D-k | gldm | DependenceNonUniformityNormalized |  |
| lbp-3D-k | gldm | DependenceVariance |  |
| lbp-3D-k | gldm | GrayLevelNonUniformity |  |
| lbp-3D-k | gldm | GrayLevelVariance |  |
| lbp-3D-k | gldm | HighGrayLevelEmphasis |  |
| lbp-3D-k | gldm | LargeDependenceEmphasis |  |
| lbp-3D-k | gldm | LargeDependenceHighGrayLevelEmphasis |  |
| lbp-3D-k | gldm | LargeDependenceLowGrayLevelEmphasis |  |
| lbp-3D-k | gldm | LowGrayLevelEmphasis |  |
| lbp-3D-k | gldm | SmallDependenceEmphasis |  |
| lbp-3D-k | gldm | SmallDependenceHighGrayLevelEmphasis |  |
| lbp-3D-k | gldm | SmallDependenceLowGrayLevelEmphasis |  |
| lbp-3D-k | ngtdm | Busyness |  |
| lbp-3D-k | ngtdm | Coarseness |  |
| lbp-3D-k | ngtdm | Complexity |  |
| lbp-3D-k | ngtdm | Contrast |  |
| lbp-3D-k | ngtdm | Strength |  |

| Supplemental Table 6. T2-radiomics features | | | |
| --- | --- | --- | --- |
|  |  |  |  |
| Filter | **Feature type** | **Feature** |  |
| original | shape | Elongation |  |
| original | shape | Flatness |  |
| original | shape | LeastAxisLength |  |
| original | shape | MajorAxisLength |  |
| original | shape | Maximum2DDiameterColumn |  |
| original | shape | Maximum2DDiameterRow |  |
| original | shape | Maximum2DDiameterSlice |  |
| original | shape | Maximum3DDiameter |  |
| original | shape | MeshVolume |  |
| original | shape | MinorAxisLength |  |
| original | shape | Sphericity |  |
| original | shape | SurfaceArea |  |
| original | shape | SurfaceVolumeRatio |  |
| original | shape | VoxelVolume |  |
| original | glcm | Autocorrelation |  |
| original | glcm | ClusterProminence |  |
| original | glcm | ClusterShade |  |
| original | glcm | ClusterTendency |  |
| original | glcm | Contrast |  |
| original | glcm | Correlation |  |
| original | glcm | DifferenceAverage |  |
| original | glcm | DifferenceEntropy |  |
| original | glcm | DifferenceVariance |  |
| original | glcm | Id |  |
| original | glcm | Idm |  |
| original | glcm | Idmn |  |
| original | glcm | Idn |  |
| original | glcm | Imc1 |  |
| original | glcm | Imc2 |  |
| original | glcm | InverseVariance |  |
| original | glcm | JointAverage |  |
| original | glcm | JointEnergy |  |
| original | glcm | JointEntropy |  |
| original | glcm | MCC |  |
| original | glcm | MaximumProbability |  |
| original | glcm | SumAverage |  |
| original | glcm | SumEntropy |  |
| original | glcm | SumSquares |  |
| original | glrlm | GrayLevelNonUniformity |  |
| original | glrlm | GrayLevelNonUniformityNormalized |  |
| original | glrlm | GrayLevelVariance |  |
| original | glrlm | HighGrayLevelRunEmphasis |  |
| original | glrlm | LongRunEmphasis |  |
| original | glrlm | LongRunHighGrayLevelEmphasis |  |
| original | glrlm | LongRunLowGrayLevelEmphasis |  |
| original | glrlm | LowGrayLevelRunEmphasis |  |
| original | glrlm | RunEntropy |  |
| original | glrlm | RunLengthNonUniformity |  |
| original | glrlm | RunLengthNonUniformityNormalized |  |
| original | glrlm | RunPercentage |  |
| original | glrlm | RunVariance |  |
| original | glrlm | ShortRunEmphasis |  |
| original | glrlm | ShortRunHighGrayLevelEmphasis |  |
| original | glrlm | ShortRunLowGrayLevelEmphasis |  |
| original | firstorder | 10Percentile |  |
| original | firstorder | 90Percentile |  |
| original | firstorder | Energy |  |
| original | firstorder | Entropy |  |
| original | firstorder | InterquartileRange |  |
| original | firstorder | Kurtosis |  |
| original | firstorder | Maximum |  |
| original | firstorder | MeanAbsoluteDeviation |  |
| original | firstorder | Mean |  |
| original | firstorder | Median |  |
| original | firstorder | Minimum |  |
| original | firstorder | Range |  |
| original | firstorder | RobustMeanAbsoluteDeviation |  |
| original | firstorder | RootMeanSquared |  |
| original | firstorder | Skewness |  |
| original | firstorder | TotalEnergy |  |
| original | firstorder | Uniformity |  |
| original | firstorder | Variance |  |
| original | glszm | GrayLevelNonUniformity |  |
| original | glszm | GrayLevelNonUniformityNormalized |  |
| original | glszm | GrayLevelVariance |  |
| original | glszm | HighGrayLevelZoneEmphasis |  |
| original | glszm | LargeAreaEmphasis |  |
| original | glszm | LargeAreaHighGrayLevelEmphasis |  |
| original | glszm | LargeAreaLowGrayLevelEmphasis |  |
| original | glszm | LowGrayLevelZoneEmphasis |  |
| original | glszm | SizeZoneNonUniformity |  |
| original | glszm | SizeZoneNonUniformityNormalized |  |
| original | glszm | SmallAreaEmphasis |  |
| original | glszm | SmallAreaHighGrayLevelEmphasis |  |
| original | glszm | SmallAreaLowGrayLevelEmphasis |  |
| original | glszm | ZoneEntropy |  |
| original | glszm | ZonePercentage |  |
| original | glszm | ZoneVariance |  |
| original | gldm | DependenceEntropy |  |
| original | gldm | DependenceNonUniformity |  |
| original | gldm | DependenceNonUniformityNormalized |  |
| original | gldm | DependenceVariance |  |
| original | gldm | GrayLevelNonUniformity |  |
| original | gldm | GrayLevelVariance |  |
| original | gldm | HighGrayLevelEmphasis |  |
| original | gldm | LargeDependenceEmphasis |  |
| original | gldm | LargeDependenceHighGrayLevelEmphasis |  |
| original | gldm | LargeDependenceLowGrayLevelEmphasis |  |
| original | gldm | LowGrayLevelEmphasis |  |
| original | gldm | SmallDependenceEmphasis |  |
| original | gldm | SmallDependenceHighGrayLevelEmphasis |  |
| original | gldm | SmallDependenceLowGrayLevelEmphasis |  |
| original | ngtdm | Busyness |  |
| original | ngtdm | Coarseness |  |
| original | ngtdm | Complexity |  |
| original | ngtdm | Contrast |  |
| original | ngtdm | Strength |  |
| wavelet-LH | glcm | Autocorrelation |  |
| wavelet-LH | glcm | ClusterProminence |  |
| wavelet-LH | glcm | ClusterShade |  |
| wavelet-LH | glcm | ClusterTendency |  |
| wavelet-LH | glcm | Contrast |  |
| wavelet-LH | glcm | Correlation |  |
| wavelet-LH | glcm | DifferenceAverage |  |
| wavelet-LH | glcm | DifferenceEntropy |  |
| wavelet-LH | glcm | DifferenceVariance |  |
| wavelet-LH | glcm | Id |  |
| wavelet-LH | glcm | Idm |  |
| wavelet-LH | glcm | Idmn |  |
| wavelet-LH | glcm | Idn |  |
| wavelet-LH | glcm | Imc1 |  |
| wavelet-LH | glcm | Imc2 |  |
| wavelet-LH | glcm | InverseVariance |  |
| wavelet-LH | glcm | JointAverage |  |
| wavelet-LH | glcm | JointEnergy |  |
| wavelet-LH | glcm | JointEntropy |  |
| wavelet-LH | glcm | MCC |  |
| wavelet-LH | glcm | MaximumProbability |  |
| wavelet-LH | glcm | SumAverage |  |
| wavelet-LH | glcm | SumEntropy |  |
| wavelet-LH | glcm | SumSquares |  |
| wavelet-LH | glrlm | GrayLevelNonUniformity |  |
| wavelet-LH | glrlm | GrayLevelNonUniformityNormalized |  |
| wavelet-LH | glrlm | GrayLevelVariance |  |
| wavelet-LH | glrlm | HighGrayLevelRunEmphasis |  |
| wavelet-LH | glrlm | LongRunEmphasis |  |
| wavelet-LH | glrlm | LongRunHighGrayLevelEmphasis |  |
| wavelet-LH | glrlm | LongRunLowGrayLevelEmphasis |  |
| wavelet-LH | glrlm | LowGrayLevelRunEmphasis |  |
| wavelet-LH | glrlm | RunEntropy |  |
| wavelet-LH | glrlm | RunLengthNonUniformity |  |
| wavelet-LH | glrlm | RunLengthNonUniformityNormalized |  |
| wavelet-LH | glrlm | RunPercentage |  |
| wavelet-LH | glrlm | RunVariance |  |
| wavelet-LH | glrlm | ShortRunEmphasis |  |
| wavelet-LH | glrlm | ShortRunHighGrayLevelEmphasis |  |
| wavelet-LH | glrlm | ShortRunLowGrayLevelEmphasis |  |
| wavelet-LH | firstorder | 10Percentile |  |
| wavelet-LH | firstorder | 90Percentile |  |
| wavelet-LH | firstorder | Energy |  |
| wavelet-LH | firstorder | Entropy |  |
| wavelet-LH | firstorder | InterquartileRange |  |
| wavelet-LH | firstorder | Kurtosis |  |
| wavelet-LH | firstorder | Maximum |  |
| wavelet-LH | firstorder | MeanAbsoluteDeviation |  |
| wavelet-LH | firstorder | Mean |  |
| wavelet-LH | firstorder | Median |  |
| wavelet-LH | firstorder | Minimum |  |
| wavelet-LH | firstorder | Range |  |
| wavelet-LH | firstorder | RobustMeanAbsoluteDeviation |  |
| wavelet-LH | firstorder | RootMeanSquared |  |
| wavelet-LH | firstorder | Skewness |  |
| wavelet-LH | firstorder | TotalEnergy |  |
| wavelet-LH | firstorder | Uniformity |  |
| wavelet-LH | firstorder | Variance |  |
| wavelet-LH | glszm | GrayLevelNonUniformity |  |
| wavelet-LH | glszm | GrayLevelNonUniformityNormalized |  |
| wavelet-LH | glszm | GrayLevelVariance |  |
| wavelet-LH | glszm | HighGrayLevelZoneEmphasis |  |
| wavelet-LH | glszm | LargeAreaEmphasis |  |
| wavelet-LH | glszm | LargeAreaHighGrayLevelEmphasis |  |
| wavelet-LH | glszm | LargeAreaLowGrayLevelEmphasis |  |
| wavelet-LH | glszm | LowGrayLevelZoneEmphasis |  |
| wavelet-LH | glszm | SizeZoneNonUniformity |  |
| wavelet-LH | glszm | SizeZoneNonUniformityNormalized |  |
| wavelet-LH | glszm | SmallAreaEmphasis |  |
| wavelet-LH | glszm | SmallAreaHighGrayLevelEmphasis |  |
| wavelet-LH | glszm | SmallAreaLowGrayLevelEmphasis |  |
| wavelet-LH | glszm | ZoneEntropy |  |
| wavelet-LH | glszm | ZonePercentage |  |
| wavelet-LH | glszm | ZoneVariance |  |
| wavelet-LH | gldm | DependenceEntropy |  |
| wavelet-LH | gldm | DependenceNonUniformity |  |
| wavelet-LH | gldm | DependenceNonUniformityNormalized |  |
| wavelet-LH | gldm | DependenceVariance |  |
| wavelet-LH | gldm | GrayLevelNonUniformity |  |
| wavelet-LH | gldm | GrayLevelVariance |  |
| wavelet-LH | gldm | HighGrayLevelEmphasis |  |
| wavelet-LH | gldm | LargeDependenceEmphasis |  |
| wavelet-LH | gldm | LargeDependenceHighGrayLevelEmphasis |  |
| wavelet-LH | gldm | LargeDependenceLowGrayLevelEmphasis |  |
| wavelet-LH | gldm | LowGrayLevelEmphasis |  |
| wavelet-LH | gldm | SmallDependenceEmphasis |  |
| wavelet-LH | gldm | SmallDependenceHighGrayLevelEmphasis |  |
| wavelet-LH | gldm | SmallDependenceLowGrayLevelEmphasis |  |
| wavelet-LH | ngtdm | Busyness |  |
| wavelet-LH | ngtdm | Coarseness |  |
| wavelet-LH | ngtdm | Complexity |  |
| wavelet-LH | ngtdm | Contrast |  |
| wavelet-LH | ngtdm | Strength |  |
| wavelet-HL | glcm | Autocorrelation |  |
| wavelet-HL | glcm | ClusterProminence |  |
| wavelet-HL | glcm | ClusterShade |  |
| wavelet-HL | glcm | ClusterTendency |  |
| wavelet-HL | glcm | Contrast |  |
| wavelet-HL | glcm | Correlation |  |
| wavelet-HL | glcm | DifferenceAverage |  |
| wavelet-HL | glcm | DifferenceEntropy |  |
| wavelet-HL | glcm | DifferenceVariance |  |
| wavelet-HL | glcm | Id |  |
| wavelet-HL | glcm | Idm |  |
| wavelet-HL | glcm | Idmn |  |
| wavelet-HL | glcm | Idn |  |
| wavelet-HL | glcm | Imc1 |  |
| wavelet-HL | glcm | Imc2 |  |
| wavelet-HL | glcm | InverseVariance |  |
| wavelet-HL | glcm | JointAverage |  |
| wavelet-HL | glcm | JointEnergy |  |
| wavelet-HL | glcm | JointEntropy |  |
| wavelet-HL | glcm | MCC |  |
| wavelet-HL | glcm | MaximumProbability |  |
| wavelet-HL | glcm | SumAverage |  |
| wavelet-HL | glcm | SumEntropy |  |
| wavelet-HL | glcm | SumSquares |  |
| wavelet-HL | glrlm | GrayLevelNonUniformity |  |
| wavelet-HL | glrlm | GrayLevelNonUniformityNormalized |  |
| wavelet-HL | glrlm | GrayLevelVariance |  |
| wavelet-HL | glrlm | HighGrayLevelRunEmphasis |  |
| wavelet-HL | glrlm | LongRunEmphasis |  |
| wavelet-HL | glrlm | LongRunHighGrayLevelEmphasis |  |
| wavelet-HL | glrlm | LongRunLowGrayLevelEmphasis |  |
| wavelet-HL | glrlm | LowGrayLevelRunEmphasis |  |
| wavelet-HL | glrlm | RunEntropy |  |
| wavelet-HL | glrlm | RunLengthNonUniformity |  |
| wavelet-HL | glrlm | RunLengthNonUniformityNormalized |  |
| wavelet-HL | glrlm | RunPercentage |  |
| wavelet-HL | glrlm | RunVariance |  |
| wavelet-HL | glrlm | ShortRunEmphasis |  |
| wavelet-HL | glrlm | ShortRunHighGrayLevelEmphasis |  |
| wavelet-HL | glrlm | ShortRunLowGrayLevelEmphasis |  |
| wavelet-HL | firstorder | 10Percentile |  |
| wavelet-HL | firstorder | 90Percentile |  |
| wavelet-HL | firstorder | Energy |  |
| wavelet-HL | firstorder | Entropy |  |
| wavelet-HL | firstorder | InterquartileRange |  |
| wavelet-HL | firstorder | Kurtosis |  |
| wavelet-HL | firstorder | Maximum |  |
| wavelet-HL | firstorder | MeanAbsoluteDeviation |  |
| wavelet-HL | firstorder | Mean |  |
| wavelet-HL | firstorder | Median |  |
| wavelet-HL | firstorder | Minimum |  |
| wavelet-HL | firstorder | Range |  |
| wavelet-HL | firstorder | RobustMeanAbsoluteDeviation |  |
| wavelet-HL | firstorder | RootMeanSquared |  |
| wavelet-HL | firstorder | Skewness |  |
| wavelet-HL | firstorder | TotalEnergy |  |
| wavelet-HL | firstorder | Uniformity |  |
| wavelet-HL | firstorder | Variance |  |
| wavelet-HL | glszm | GrayLevelNonUniformity |  |
| wavelet-HL | glszm | GrayLevelNonUniformityNormalized |  |
| wavelet-HL | glszm | GrayLevelVariance |  |
| wavelet-HL | glszm | HighGrayLevelZoneEmphasis |  |
| wavelet-HL | glszm | LargeAreaEmphasis |  |
| wavelet-HL | glszm | LargeAreaHighGrayLevelEmphasis |  |
| wavelet-HL | glszm | LargeAreaLowGrayLevelEmphasis |  |
| wavelet-HL | glszm | LowGrayLevelZoneEmphasis |  |
| wavelet-HL | glszm | SizeZoneNonUniformity |  |
| wavelet-HL | glszm | SizeZoneNonUniformityNormalized |  |
| wavelet-HL | glszm | SmallAreaEmphasis |  |
| wavelet-HL | glszm | SmallAreaHighGrayLevelEmphasis |  |
| wavelet-HL | glszm | SmallAreaLowGrayLevelEmphasis |  |
| wavelet-HL | glszm | ZoneEntropy |  |
| wavelet-HL | glszm | ZonePercentage |  |
| wavelet-HL | glszm | ZoneVariance |  |
| wavelet-HL | gldm | DependenceEntropy |  |
| wavelet-HL | gldm | DependenceNonUniformity |  |
| wavelet-HL | gldm | DependenceNonUniformityNormalized |  |
| wavelet-HL | gldm | DependenceVariance |  |
| wavelet-HL | gldm | GrayLevelNonUniformity |  |
| wavelet-HL | gldm | GrayLevelVariance |  |
| wavelet-HL | gldm | HighGrayLevelEmphasis |  |
| wavelet-HL | gldm | LargeDependenceEmphasis |  |
| wavelet-HL | gldm | LargeDependenceHighGrayLevelEmphasis |  |
| wavelet-HL | gldm | LargeDependenceLowGrayLevelEmphasis |  |
| wavelet-HL | gldm | LowGrayLevelEmphasis |  |
| wavelet-HL | gldm | SmallDependenceEmphasis |  |
| wavelet-HL | gldm | SmallDependenceHighGrayLevelEmphasis |  |
| wavelet-HL | gldm | SmallDependenceLowGrayLevelEmphasis |  |
| wavelet-HL | ngtdm | Busyness |  |
| wavelet-HL | ngtdm | Coarseness |  |
| wavelet-HL | ngtdm | Complexity |  |
| wavelet-HL | ngtdm | Contrast |  |
| wavelet-HL | ngtdm | Strength |  |
| wavelet-HH | glcm | Autocorrelation |  |
| wavelet-HH | glcm | ClusterProminence |  |
| wavelet-HH | glcm | ClusterShade |  |
| wavelet-HH | glcm | ClusterTendency |  |
| wavelet-HH | glcm | Contrast |  |
| wavelet-HH | glcm | Correlation |  |
| wavelet-HH | glcm | DifferenceAverage |  |
| wavelet-HH | glcm | DifferenceEntropy |  |
| wavelet-HH | glcm | DifferenceVariance |  |
| wavelet-HH | glcm | Id |  |
| wavelet-HH | glcm | Idm |  |
| wavelet-HH | glcm | Idmn |  |
| wavelet-HH | glcm | Idn |  |
| wavelet-HH | glcm | Imc1 |  |
| wavelet-HH | glcm | Imc2 |  |
| wavelet-HH | glcm | InverseVariance |  |
| wavelet-HH | glcm | JointAverage |  |
| wavelet-HH | glcm | JointEnergy |  |
| wavelet-HH | glcm | JointEntropy |  |
| wavelet-HH | glcm | MCC |  |
| wavelet-HH | glcm | MaximumProbability |  |
| wavelet-HH | glcm | SumAverage |  |
| wavelet-HH | glcm | SumEntropy |  |
| wavelet-HH | glcm | SumSquares |  |
| wavelet-HH | glrlm | GrayLevelNonUniformity |  |
| wavelet-HH | glrlm | GrayLevelNonUniformityNormalized |  |
| wavelet-HH | glrlm | GrayLevelVariance |  |
| wavelet-HH | glrlm | HighGrayLevelRunEmphasis |  |
| wavelet-HH | glrlm | LongRunEmphasis |  |
| wavelet-HH | glrlm | LongRunHighGrayLevelEmphasis |  |
| wavelet-HH | glrlm | LongRunLowGrayLevelEmphasis |  |
| wavelet-HH | glrlm | LowGrayLevelRunEmphasis |  |
| wavelet-HH | glrlm | RunEntropy |  |
| wavelet-HH | glrlm | RunLengthNonUniformity |  |
| wavelet-HH | glrlm | RunLengthNonUniformityNormalized |  |
| wavelet-HH | glrlm | RunPercentage |  |
| wavelet-HH | glrlm | RunVariance |  |
| wavelet-HH | glrlm | ShortRunEmphasis |  |
| wavelet-HH | glrlm | ShortRunHighGrayLevelEmphasis |  |
| wavelet-HH | glrlm | ShortRunLowGrayLevelEmphasis |  |
| wavelet-HH | firstorder | 10Percentile |  |
| wavelet-HH | firstorder | 90Percentile |  |
| wavelet-HH | firstorder | Energy |  |
| wavelet-HH | firstorder | Entropy |  |
| wavelet-HH | firstorder | InterquartileRange |  |
| wavelet-HH | firstorder | Kurtosis |  |
| wavelet-HH | firstorder | Maximum |  |
| wavelet-HH | firstorder | MeanAbsoluteDeviation |  |
| wavelet-HH | firstorder | Mean |  |
| wavelet-HH | firstorder | Median |  |
| wavelet-HH | firstorder | Minimum |  |
| wavelet-HH | firstorder | Range |  |
| wavelet-HH | firstorder | RobustMeanAbsoluteDeviation |  |
| wavelet-HH | firstorder | RootMeanSquared |  |
| wavelet-HH | firstorder | Skewness |  |
| wavelet-HH | firstorder | TotalEnergy |  |
| wavelet-HH | firstorder | Uniformity |  |
| wavelet-HH | firstorder | Variance |  |
| wavelet-HH | glszm | GrayLevelNonUniformity |  |
| wavelet-HH | glszm | GrayLevelNonUniformityNormalized |  |
| wavelet-HH | glszm | GrayLevelVariance |  |
| wavelet-HH | glszm | HighGrayLevelZoneEmphasis |  |
| wavelet-HH | glszm | LargeAreaEmphasis |  |
| wavelet-HH | glszm | LargeAreaHighGrayLevelEmphasis |  |
| wavelet-HH | glszm | LargeAreaLowGrayLevelEmphasis |  |
| wavelet-HH | glszm | LowGrayLevelZoneEmphasis |  |
| wavelet-HH | glszm | SizeZoneNonUniformity |  |
| wavelet-HH | glszm | SizeZoneNonUniformityNormalized |  |
| wavelet-HH | glszm | SmallAreaEmphasis |  |
| wavelet-HH | glszm | SmallAreaHighGrayLevelEmphasis |  |
| wavelet-HH | glszm | SmallAreaLowGrayLevelEmphasis |  |
| wavelet-HH | glszm | ZoneEntropy |  |
| wavelet-HH | glszm | ZonePercentage |  |
| wavelet-HH | glszm | ZoneVariance |  |
| wavelet-HH | gldm | DependenceEntropy |  |
| wavelet-HH | gldm | DependenceNonUniformity |  |
| wavelet-HH | gldm | DependenceNonUniformityNormalized |  |
| wavelet-HH | gldm | DependenceVariance |  |
| wavelet-HH | gldm | GrayLevelNonUniformity |  |
| wavelet-HH | gldm | GrayLevelVariance |  |
| wavelet-HH | gldm | HighGrayLevelEmphasis |  |
| wavelet-HH | gldm | LargeDependenceEmphasis |  |
| wavelet-HH | gldm | LargeDependenceHighGrayLevelEmphasis |  |
| wavelet-HH | gldm | LargeDependenceLowGrayLevelEmphasis |  |
| wavelet-HH | gldm | LowGrayLevelEmphasis |  |
| wavelet-HH | gldm | SmallDependenceEmphasis |  |
| wavelet-HH | gldm | SmallDependenceHighGrayLevelEmphasis |  |
| wavelet-HH | gldm | SmallDependenceLowGrayLevelEmphasis |  |
| wavelet-HH | ngtdm | Busyness |  |
| wavelet-HH | ngtdm | Coarseness |  |
| wavelet-HH | ngtdm | Complexity |  |
| wavelet-HH | ngtdm | Contrast |  |
| wavelet-HH | ngtdm | Strength |  |
| wavelet-LL | glcm | Autocorrelation |  |
| wavelet-LL | glcm | ClusterProminence |  |
| wavelet-LL | glcm | ClusterShade |  |
| wavelet-LL | glcm | ClusterTendency |  |
| wavelet-LL | glcm | Contrast |  |
| wavelet-LL | glcm | Correlation |  |
| wavelet-LL | glcm | DifferenceAverage |  |
| wavelet-LL | glcm | DifferenceEntropy |  |
| wavelet-LL | glcm | DifferenceVariance |  |
| wavelet-LL | glcm | Id |  |
| wavelet-LL | glcm | Idm |  |
| wavelet-LL | glcm | Idmn |  |
| wavelet-LL | glcm | Idn |  |
| wavelet-LL | glcm | Imc1 |  |
| wavelet-LL | glcm | Imc2 |  |
| wavelet-LL | glcm | InverseVariance |  |
| wavelet-LL | glcm | JointAverage |  |
| wavelet-LL | glcm | JointEnergy |  |
| wavelet-LL | glcm | JointEntropy |  |
| wavelet-LL | glcm | MCC |  |
| wavelet-LL | glcm | MaximumProbability |  |
| wavelet-LL | glcm | SumAverage |  |
| wavelet-LL | glcm | SumEntropy |  |
| wavelet-LL | glcm | SumSquares |  |
| wavelet-LL | glrlm | GrayLevelNonUniformity |  |
| wavelet-LL | glrlm | GrayLevelNonUniformityNormalized |  |
| wavelet-LL | glrlm | GrayLevelVariance |  |
| wavelet-LL | glrlm | HighGrayLevelRunEmphasis |  |
| wavelet-LL | glrlm | LongRunEmphasis |  |
| wavelet-LL | glrlm | LongRunHighGrayLevelEmphasis |  |
| wavelet-LL | glrlm | LongRunLowGrayLevelEmphasis |  |
| wavelet-LL | glrlm | LowGrayLevelRunEmphasis |  |
| wavelet-LL | glrlm | RunEntropy |  |
| wavelet-LL | glrlm | RunLengthNonUniformity |  |
| wavelet-LL | glrlm | RunLengthNonUniformityNormalized |  |
| wavelet-LL | glrlm | RunPercentage |  |
| wavelet-LL | glrlm | RunVariance |  |
| wavelet-LL | glrlm | ShortRunEmphasis |  |
| wavelet-LL | glrlm | ShortRunHighGrayLevelEmphasis |  |
| wavelet-LL | glrlm | ShortRunLowGrayLevelEmphasis |  |
| wavelet-LL | firstorder | 10Percentile |  |
| wavelet-LL | firstorder | 90Percentile |  |
| wavelet-LL | firstorder | Energy |  |
| wavelet-LL | firstorder | Entropy |  |
| wavelet-LL | firstorder | InterquartileRange |  |
| wavelet-LL | firstorder | Kurtosis |  |
| wavelet-LL | firstorder | Maximum |  |
| wavelet-LL | firstorder | MeanAbsoluteDeviation |  |
| wavelet-LL | firstorder | Mean |  |
| wavelet-LL | firstorder | Median |  |
| wavelet-LL | firstorder | Minimum |  |
| wavelet-LL | firstorder | Range |  |
| wavelet-LL | firstorder | RobustMeanAbsoluteDeviation |  |
| wavelet-LL | firstorder | RootMeanSquared |  |
| wavelet-LL | firstorder | Skewness |  |
| wavelet-LL | firstorder | TotalEnergy |  |
| wavelet-LL | firstorder | Uniformity |  |
| wavelet-LL | firstorder | Variance |  |
| wavelet-LL | glszm | GrayLevelNonUniformity |  |
| wavelet-LL | glszm | GrayLevelNonUniformityNormalized |  |
| wavelet-LL | glszm | GrayLevelVariance |  |
| wavelet-LL | glszm | HighGrayLevelZoneEmphasis |  |
| wavelet-LL | glszm | LargeAreaEmphasis |  |
| wavelet-LL | glszm | LargeAreaHighGrayLevelEmphasis |  |
| wavelet-LL | glszm | LargeAreaLowGrayLevelEmphasis |  |
| wavelet-LL | glszm | LowGrayLevelZoneEmphasis |  |
| wavelet-LL | glszm | SizeZoneNonUniformity |  |
| wavelet-LL | glszm | SizeZoneNonUniformityNormalized |  |
| wavelet-LL | glszm | SmallAreaEmphasis |  |
| wavelet-LL | glszm | SmallAreaHighGrayLevelEmphasis |  |
| wavelet-LL | glszm | SmallAreaLowGrayLevelEmphasis |  |
| wavelet-LL | glszm | ZoneEntropy |  |
| wavelet-LL | glszm | ZonePercentage |  |
| wavelet-LL | glszm | ZoneVariance |  |
| wavelet-LL | gldm | DependenceEntropy |  |
| wavelet-LL | gldm | DependenceNonUniformity |  |
| wavelet-LL | gldm | DependenceNonUniformityNormalized |  |
| wavelet-LL | gldm | DependenceVariance |  |
| wavelet-LL | gldm | GrayLevelNonUniformity |  |
| wavelet-LL | gldm | GrayLevelVariance |  |
| wavelet-LL | gldm | HighGrayLevelEmphasis |  |
| wavelet-LL | gldm | LargeDependenceEmphasis |  |
| wavelet-LL | gldm | LargeDependenceHighGrayLevelEmphasis |  |
| wavelet-LL | gldm | LargeDependenceLowGrayLevelEmphasis |  |
| wavelet-LL | gldm | LowGrayLevelEmphasis |  |
| wavelet-LL | gldm | SmallDependenceEmphasis |  |
| wavelet-LL | gldm | SmallDependenceHighGrayLevelEmphasis |  |
| wavelet-LL | gldm | SmallDependenceLowGrayLevelEmphasis |  |
| wavelet-LL | ngtdm | Busyness |  |
| wavelet-LL | ngtdm | Coarseness |  |
| wavelet-LL | ngtdm | Complexity |  |
| wavelet-LL | ngtdm | Contrast |  |
| wavelet-LL | ngtdm | Strength |  |
| square | glrlm | GrayLevelNonUniformity |  |
| square | glrlm | LongRunEmphasis |  |
| square | glrlm | LongRunHighGrayLevelEmphasis |  |
| square | glrlm | LongRunLowGrayLevelEmphasis |  |
| square | glrlm | RunEntropy |  |
| square | glrlm | RunLengthNonUniformity |  |
| square | glrlm | RunLengthNonUniformityNormalized |  |
| square | glrlm | RunPercentage |  |
| square | glrlm | RunVariance |  |
| square | glrlm | ShortRunEmphasis |  |
| square | glrlm | ShortRunHighGrayLevelEmphasis |  |
| square | glrlm | ShortRunLowGrayLevelEmphasis |  |
| square | firstorder | 10Percentile |  |
| square | firstorder | 90Percentile |  |
| square | firstorder | Energy |  |
| square | firstorder | InterquartileRange |  |
| square | firstorder | Kurtosis |  |
| square | firstorder | Maximum |  |
| square | firstorder | MeanAbsoluteDeviation |  |
| square | firstorder | Mean |  |
| square | firstorder | Median |  |
| square | firstorder | Minimum |  |
| square | firstorder | Range |  |
| square | firstorder | RobustMeanAbsoluteDeviation |  |
| square | firstorder | RootMeanSquared |  |
| square | firstorder | Skewness |  |
| square | firstorder | TotalEnergy |  |
| square | firstorder | Variance |  |
| square | glszm | GrayLevelNonUniformity |  |
| square | glszm | LargeAreaEmphasis |  |
| square | glszm | LargeAreaHighGrayLevelEmphasis |  |
| square | glszm | LargeAreaLowGrayLevelEmphasis |  |
| square | glszm | SizeZoneNonUniformity |  |
| square | glszm | SizeZoneNonUniformityNormalized |  |
| square | glszm | SmallAreaEmphasis |  |
| square | glszm | SmallAreaHighGrayLevelEmphasis |  |
| square | glszm | SmallAreaLowGrayLevelEmphasis |  |
| square | glszm | ZoneEntropy |  |
| square | glszm | ZonePercentage |  |
| square | glszm | ZoneVariance |  |
| square | gldm | DependenceEntropy |  |
| square | gldm | DependenceNonUniformity |  |
| square | gldm | DependenceNonUniformityNormalized |  |
| square | gldm | DependenceVariance |  |
| square | gldm | GrayLevelNonUniformity |  |
| square | gldm | LargeDependenceEmphasis |  |
| square | gldm | LargeDependenceHighGrayLevelEmphasis |  |
| square | gldm | LargeDependenceLowGrayLevelEmphasis |  |
| square | gldm | SmallDependenceEmphasis |  |
| square | gldm | SmallDependenceHighGrayLevelEmphasis |  |
| square | gldm | SmallDependenceLowGrayLevelEmphasis |  |
| squareroot | glcm | Autocorrelation |  |
| squareroot | glcm | ClusterProminence |  |
| squareroot | glcm | ClusterShade |  |
| squareroot | glcm | ClusterTendency |  |
| squareroot | glcm | Contrast |  |
| squareroot | glcm | Correlation |  |
| squareroot | glcm | DifferenceAverage |  |
| squareroot | glcm | DifferenceEntropy |  |
| squareroot | glcm | DifferenceVariance |  |
| squareroot | glcm | Id |  |
| squareroot | glcm | Idm |  |
| squareroot | glcm | Idmn |  |
| squareroot | glcm | Idn |  |
| squareroot | glcm | Imc1 |  |
| squareroot | glcm | Imc2 |  |
| squareroot | glcm | InverseVariance |  |
| squareroot | glcm | JointAverage |  |
| squareroot | glcm | JointEnergy |  |
| squareroot | glcm | JointEntropy |  |
| squareroot | glcm | MCC |  |
| squareroot | glcm | MaximumProbability |  |
| squareroot | glcm | SumAverage |  |
| squareroot | glcm | SumEntropy |  |
| squareroot | glcm | SumSquares |  |
| squareroot | glrlm | GrayLevelNonUniformity |  |
| squareroot | glrlm | GrayLevelNonUniformityNormalized |  |
| squareroot | glrlm | GrayLevelVariance |  |
| squareroot | glrlm | HighGrayLevelRunEmphasis |  |
| squareroot | glrlm | LongRunEmphasis |  |
| squareroot | glrlm | LongRunHighGrayLevelEmphasis |  |
| squareroot | glrlm | LongRunLowGrayLevelEmphasis |  |
| squareroot | glrlm | LowGrayLevelRunEmphasis |  |
| squareroot | glrlm | RunEntropy |  |
| squareroot | glrlm | RunLengthNonUniformity |  |
| squareroot | glrlm | RunLengthNonUniformityNormalized |  |
| squareroot | glrlm | RunPercentage |  |
| squareroot | glrlm | RunVariance |  |
| squareroot | glrlm | ShortRunEmphasis |  |
| squareroot | glrlm | ShortRunHighGrayLevelEmphasis |  |
| squareroot | glrlm | ShortRunLowGrayLevelEmphasis |  |
| squareroot | firstorder | 10Percentile |  |
| squareroot | firstorder | 90Percentile |  |
| squareroot | firstorder | Energy |  |
| squareroot | firstorder | Entropy |  |
| squareroot | firstorder | InterquartileRange |  |
| squareroot | firstorder | Kurtosis |  |
| squareroot | firstorder | Maximum |  |
| squareroot | firstorder | MeanAbsoluteDeviation |  |
| squareroot | firstorder | Mean |  |
| squareroot | firstorder | Median |  |
| squareroot | firstorder | Minimum |  |
| squareroot | firstorder | Range |  |
| squareroot | firstorder | RobustMeanAbsoluteDeviation |  |
| squareroot | firstorder | RootMeanSquared |  |
| squareroot | firstorder | Skewness |  |
| squareroot | firstorder | TotalEnergy |  |
| squareroot | firstorder | Uniformity |  |
| squareroot | firstorder | Variance |  |
| squareroot | glszm | GrayLevelNonUniformity |  |
| squareroot | glszm | GrayLevelNonUniformityNormalized |  |
| squareroot | glszm | GrayLevelVariance |  |
| squareroot | glszm | HighGrayLevelZoneEmphasis |  |
| squareroot | glszm | LargeAreaEmphasis |  |
| squareroot | glszm | LargeAreaHighGrayLevelEmphasis |  |
| squareroot | glszm | LargeAreaLowGrayLevelEmphasis |  |
| squareroot | glszm | LowGrayLevelZoneEmphasis |  |
| squareroot | glszm | SizeZoneNonUniformity |  |
| squareroot | glszm | SizeZoneNonUniformityNormalized |  |
| squareroot | glszm | SmallAreaEmphasis |  |
| squareroot | glszm | SmallAreaHighGrayLevelEmphasis |  |
| squareroot | glszm | SmallAreaLowGrayLevelEmphasis |  |
| squareroot | glszm | ZoneEntropy |  |
| squareroot | glszm | ZonePercentage |  |
| squareroot | glszm | ZoneVariance |  |
| squareroot | gldm | DependenceEntropy |  |
| squareroot | gldm | DependenceNonUniformity |  |
| squareroot | gldm | DependenceNonUniformityNormalized |  |
| squareroot | gldm | DependenceVariance |  |
| squareroot | gldm | GrayLevelNonUniformity |  |
| squareroot | gldm | GrayLevelVariance |  |
| squareroot | gldm | HighGrayLevelEmphasis |  |
| squareroot | gldm | LargeDependenceEmphasis |  |
| squareroot | gldm | LargeDependenceHighGrayLevelEmphasis |  |
| squareroot | gldm | LargeDependenceLowGrayLevelEmphasis |  |
| squareroot | gldm | LowGrayLevelEmphasis |  |
| squareroot | gldm | SmallDependenceEmphasis |  |
| squareroot | gldm | SmallDependenceHighGrayLevelEmphasis |  |
| squareroot | gldm | SmallDependenceLowGrayLevelEmphasis |  |
| squareroot | ngtdm | Busyness |  |
| squareroot | ngtdm | Coarseness |  |
| squareroot | ngtdm | Complexity |  |
| squareroot | ngtdm | Contrast |  |
| squareroot | ngtdm | Strength |  |
| logarithm | glcm | Autocorrelation |  |
| logarithm | glcm | ClusterProminence |  |
| logarithm | glcm | ClusterShade |  |
| logarithm | glcm | ClusterTendency |  |
| logarithm | glcm | Contrast |  |
| logarithm | glcm | Correlation |  |
| logarithm | glcm | DifferenceAverage |  |
| logarithm | glcm | DifferenceEntropy |  |
| logarithm | glcm | DifferenceVariance |  |
| logarithm | glcm | Id |  |
| logarithm | glcm | Idm |  |
| logarithm | glcm | Idmn |  |
| logarithm | glcm | Idn |  |
| logarithm | glcm | Imc1 |  |
| logarithm | glcm | Imc2 |  |
| logarithm | glcm | InverseVariance |  |
| logarithm | glcm | JointAverage |  |
| logarithm | glcm | JointEnergy |  |
| logarithm | glcm | JointEntropy |  |
| logarithm | glcm | MCC |  |
| logarithm | glcm | MaximumProbability |  |
| logarithm | glcm | SumAverage |  |
| logarithm | glcm | SumEntropy |  |
| logarithm | glcm | SumSquares |  |
| logarithm | glrlm | GrayLevelNonUniformity |  |
| logarithm | glrlm | GrayLevelNonUniformityNormalized |  |
| logarithm | glrlm | GrayLevelVariance |  |
| logarithm | glrlm | HighGrayLevelRunEmphasis |  |
| logarithm | glrlm | LongRunEmphasis |  |
| logarithm | glrlm | LongRunHighGrayLevelEmphasis |  |
| logarithm | glrlm | LongRunLowGrayLevelEmphasis |  |
| logarithm | glrlm | LowGrayLevelRunEmphasis |  |
| logarithm | glrlm | RunEntropy |  |
| logarithm | glrlm | RunLengthNonUniformity |  |
| logarithm | glrlm | RunLengthNonUniformityNormalized |  |
| logarithm | glrlm | RunPercentage |  |
| logarithm | glrlm | RunVariance |  |
| logarithm | glrlm | ShortRunEmphasis |  |
| logarithm | glrlm | ShortRunHighGrayLevelEmphasis |  |
| logarithm | glrlm | ShortRunLowGrayLevelEmphasis |  |
| logarithm | firstorder | 10Percentile |  |
| logarithm | firstorder | 90Percentile |  |
| logarithm | firstorder | Energy |  |
| logarithm | firstorder | Entropy |  |
| logarithm | firstorder | InterquartileRange |  |
| logarithm | firstorder | Kurtosis |  |
| logarithm | firstorder | Maximum |  |
| logarithm | firstorder | MeanAbsoluteDeviation |  |
| logarithm | firstorder | Mean |  |
| logarithm | firstorder | Median |  |
| logarithm | firstorder | Minimum |  |
| logarithm | firstorder | Range |  |
| logarithm | firstorder | RobustMeanAbsoluteDeviation |  |
| logarithm | firstorder | RootMeanSquared |  |
| logarithm | firstorder | Skewness |  |
| logarithm | firstorder | TotalEnergy |  |
| logarithm | firstorder | Uniformity |  |
| logarithm | firstorder | Variance |  |
| logarithm | glszm | GrayLevelNonUniformity |  |
| logarithm | glszm | GrayLevelNonUniformityNormalized |  |
| logarithm | glszm | GrayLevelVariance |  |
| logarithm | glszm | HighGrayLevelZoneEmphasis |  |
| logarithm | glszm | LargeAreaEmphasis |  |
| logarithm | glszm | LargeAreaHighGrayLevelEmphasis |  |
| logarithm | glszm | LargeAreaLowGrayLevelEmphasis |  |
| logarithm | glszm | LowGrayLevelZoneEmphasis |  |
| logarithm | glszm | SizeZoneNonUniformity |  |
| logarithm | glszm | SizeZoneNonUniformityNormalized |  |
| logarithm | glszm | SmallAreaEmphasis |  |
| logarithm | glszm | SmallAreaHighGrayLevelEmphasis |  |
| logarithm | glszm | SmallAreaLowGrayLevelEmphasis |  |
| logarithm | glszm | ZoneEntropy |  |
| logarithm | glszm | ZonePercentage |  |
| logarithm | glszm | ZoneVariance |  |
| logarithm | gldm | DependenceEntropy |  |
| logarithm | gldm | DependenceNonUniformity |  |
| logarithm | gldm | DependenceNonUniformityNormalized |  |
| logarithm | gldm | DependenceVariance |  |
| logarithm | gldm | GrayLevelNonUniformity |  |
| logarithm | gldm | GrayLevelVariance |  |
| logarithm | gldm | HighGrayLevelEmphasis |  |
| logarithm | gldm | LargeDependenceEmphasis |  |
| logarithm | gldm | LargeDependenceHighGrayLevelEmphasis |  |
| logarithm | gldm | LargeDependenceLowGrayLevelEmphasis |  |
| logarithm | gldm | LowGrayLevelEmphasis |  |
| logarithm | gldm | SmallDependenceEmphasis |  |
| logarithm | gldm | SmallDependenceHighGrayLevelEmphasis |  |
| logarithm | gldm | SmallDependenceLowGrayLevelEmphasis |  |
| logarithm | ngtdm | Busyness |  |
| logarithm | ngtdm | Coarseness |  |
| logarithm | ngtdm | Complexity |  |
| logarithm | ngtdm | Contrast |  |
| logarithm | ngtdm | Strength |  |
| exponential | glrlm | GrayLevelNonUniformity |  |
| exponential | glrlm | LongRunEmphasis |  |
| exponential | glrlm | LongRunHighGrayLevelEmphasis |  |
| exponential | glrlm | LongRunLowGrayLevelEmphasis |  |
| exponential | glrlm | RunEntropy |  |
| exponential | glrlm | RunLengthNonUniformity |  |
| exponential | glrlm | RunLengthNonUniformityNormalized |  |
| exponential | glrlm | RunPercentage |  |
| exponential | glrlm | RunVariance |  |
| exponential | glrlm | ShortRunEmphasis |  |
| exponential | glrlm | ShortRunHighGrayLevelEmphasis |  |
| exponential | glrlm | ShortRunLowGrayLevelEmphasis |  |
| exponential | firstorder | 10Percentile |  |
| exponential | firstorder | 90Percentile |  |
| exponential | firstorder | Energy |  |
| exponential | firstorder | InterquartileRange |  |
| exponential | firstorder | Kurtosis |  |
| exponential | firstorder | Maximum |  |
| exponential | firstorder | MeanAbsoluteDeviation |  |
| exponential | firstorder | Mean |  |
| exponential | firstorder | Median |  |
| exponential | firstorder | Minimum |  |
| exponential | firstorder | Range |  |
| exponential | firstorder | RobustMeanAbsoluteDeviation |  |
| exponential | firstorder | RootMeanSquared |  |
| exponential | firstorder | Skewness |  |
| exponential | firstorder | TotalEnergy |  |
| exponential | firstorder | Variance |  |
| exponential | glszm | GrayLevelNonUniformity |  |
| exponential | glszm | LargeAreaEmphasis |  |
| exponential | glszm | LargeAreaHighGrayLevelEmphasis |  |
| exponential | glszm | LargeAreaLowGrayLevelEmphasis |  |
| exponential | glszm | SizeZoneNonUniformity |  |
| exponential | glszm | SizeZoneNonUniformityNormalized |  |
| exponential | glszm | SmallAreaEmphasis |  |
| exponential | glszm | SmallAreaHighGrayLevelEmphasis |  |
| exponential | glszm | SmallAreaLowGrayLevelEmphasis |  |
| exponential | glszm | ZoneEntropy |  |
| exponential | glszm | ZonePercentage |  |
| exponential | glszm | ZoneVariance |  |
| exponential | gldm | DependenceEntropy |  |
| exponential | gldm | DependenceNonUniformity |  |
| exponential | gldm | DependenceNonUniformityNormalized |  |
| exponential | gldm | DependenceVariance |  |
| exponential | gldm | GrayLevelNonUniformity |  |
| exponential | gldm | LargeDependenceEmphasis |  |
| exponential | gldm | LargeDependenceHighGrayLevelEmphasis |  |
| exponential | gldm | LargeDependenceLowGrayLevelEmphasis |  |
| exponential | gldm | SmallDependenceEmphasis |  |
| exponential | gldm | SmallDependenceHighGrayLevelEmphasis |  |
| exponential | gldm | SmallDependenceLowGrayLevelEmphasis |  |
| gradient | glrlm | GrayLevelNonUniformity |  |
| gradient | glrlm | LongRunEmphasis |  |
| gradient | glrlm | LongRunHighGrayLevelEmphasis |  |
| gradient | glrlm | LongRunLowGrayLevelEmphasis |  |
| gradient | glrlm | RunEntropy |  |
| gradient | glrlm | RunLengthNonUniformity |  |
| gradient | glrlm | RunLengthNonUniformityNormalized |  |
| gradient | glrlm | RunPercentage |  |
| gradient | glrlm | RunVariance |  |
| gradient | glrlm | ShortRunEmphasis |  |
| gradient | glrlm | ShortRunHighGrayLevelEmphasis |  |
| gradient | glrlm | ShortRunLowGrayLevelEmphasis |  |
| gradient | firstorder | 10Percentile |  |
| gradient | firstorder | 90Percentile |  |
| gradient | firstorder | Energy |  |
| gradient | firstorder | InterquartileRange |  |
| gradient | firstorder | Kurtosis |  |
| gradient | firstorder | Maximum |  |
| gradient | firstorder | MeanAbsoluteDeviation |  |
| gradient | firstorder | Mean |  |
| gradient | firstorder | Median |  |
| gradient | firstorder | Minimum |  |
| gradient | firstorder | Range |  |
| gradient | firstorder | RobustMeanAbsoluteDeviation |  |
| gradient | firstorder | RootMeanSquared |  |
| gradient | firstorder | Skewness |  |
| gradient | firstorder | TotalEnergy |  |
| gradient | firstorder | Variance |  |
| gradient | glszm | GrayLevelNonUniformity |  |
| gradient | glszm | LargeAreaEmphasis |  |
| gradient | glszm | LargeAreaHighGrayLevelEmphasis |  |
| gradient | glszm | LargeAreaLowGrayLevelEmphasis |  |
| gradient | glszm | SizeZoneNonUniformity |  |
| gradient | glszm | SizeZoneNonUniformityNormalized |  |
| gradient | glszm | SmallAreaEmphasis |  |
| gradient | glszm | SmallAreaHighGrayLevelEmphasis |  |
| gradient | glszm | SmallAreaLowGrayLevelEmphasis |  |
| gradient | glszm | ZoneEntropy |  |
| gradient | glszm | ZonePercentage |  |
| gradient | glszm | ZoneVariance |  |
| gradient | gldm | DependenceEntropy |  |
| gradient | gldm | DependenceNonUniformity |  |
| gradient | gldm | DependenceNonUniformityNormalized |  |
| gradient | gldm | DependenceVariance |  |
| gradient | gldm | GrayLevelNonUniformity |  |
| gradient | gldm | LargeDependenceEmphasis |  |
| gradient | gldm | LargeDependenceHighGrayLevelEmphasis |  |
| gradient | gldm | LargeDependenceLowGrayLevelEmphasis |  |
| gradient | gldm | SmallDependenceEmphasis |  |
| gradient | gldm | SmallDependenceHighGrayLevelEmphasis |  |
| gradient | gldm | SmallDependenceLowGrayLevelEmphasis |  |
| lbp-2D | glrlm | GrayLevelNonUniformity |  |
| lbp-2D | glrlm | LongRunEmphasis |  |
| lbp-2D | glrlm | LongRunHighGrayLevelEmphasis |  |
| lbp-2D | glrlm | LongRunLowGrayLevelEmphasis |  |
| lbp-2D | glrlm | RunEntropy |  |
| lbp-2D | glrlm | RunLengthNonUniformity |  |
| lbp-2D | glrlm | RunLengthNonUniformityNormalized |  |
| lbp-2D | glrlm | RunPercentage |  |
| lbp-2D | glrlm | RunVariance |  |
| lbp-2D | glrlm | ShortRunEmphasis |  |
| lbp-2D | glrlm | ShortRunHighGrayLevelEmphasis |  |
| lbp-2D | glrlm | ShortRunLowGrayLevelEmphasis |  |
| lbp-2D | firstorder | 10Percentile |  |
| lbp-2D | firstorder | 90Percentile |  |
| lbp-2D | firstorder | Energy |  |
| lbp-2D | firstorder | InterquartileRange |  |
| lbp-2D | firstorder | Kurtosis |  |
| lbp-2D | firstorder | MeanAbsoluteDeviation |  |
| lbp-2D | firstorder | Mean |  |
| lbp-2D | firstorder | Median |  |
| lbp-2D | firstorder | RobustMeanAbsoluteDeviation |  |
| lbp-2D | firstorder | RootMeanSquared |  |
| lbp-2D | firstorder | Skewness |  |
| lbp-2D | firstorder | TotalEnergy |  |
| lbp-2D | firstorder | Variance |  |
| lbp-2D | glszm | GrayLevelNonUniformity |  |
| lbp-2D | glszm | LargeAreaEmphasis |  |
| lbp-2D | glszm | LargeAreaHighGrayLevelEmphasis |  |
| lbp-2D | glszm | LargeAreaLowGrayLevelEmphasis |  |
| lbp-2D | glszm | SizeZoneNonUniformity |  |
| lbp-2D | glszm | SizeZoneNonUniformityNormalized |  |
| lbp-2D | glszm | SmallAreaEmphasis |  |
| lbp-2D | glszm | SmallAreaHighGrayLevelEmphasis |  |
| lbp-2D | glszm | SmallAreaLowGrayLevelEmphasis |  |
| lbp-2D | glszm | ZoneEntropy |  |
| lbp-2D | glszm | ZonePercentage |  |
| lbp-2D | glszm | ZoneVariance |  |
| lbp-2D | gldm | DependenceEntropy |  |
| lbp-2D | gldm | DependenceNonUniformity |  |
| lbp-2D | gldm | DependenceNonUniformityNormalized |  |
| lbp-2D | gldm | DependenceVariance |  |
| lbp-2D | gldm | GrayLevelNonUniformity |  |
| lbp-2D | gldm | LargeDependenceEmphasis |  |
| lbp-2D | gldm | LargeDependenceHighGrayLevelEmphasis |  |
| lbp-2D | gldm | LargeDependenceLowGrayLevelEmphasis |  |
| lbp-2D | gldm | SmallDependenceEmphasis |  |
| lbp-2D | gldm | SmallDependenceHighGrayLevelEmphasis |  |
| lbp-2D | gldm | SmallDependenceLowGrayLevelEmphasis |  |
| lbp-3D-m1 | glrlm | GrayLevelNonUniformity |  |
| lbp-3D-m1 | glrlm | LongRunEmphasis |  |
| lbp-3D-m1 | glrlm | LongRunHighGrayLevelEmphasis |  |
| lbp-3D-m1 | glrlm | LongRunLowGrayLevelEmphasis |  |
| lbp-3D-m1 | glrlm | RunEntropy |  |
| lbp-3D-m1 | glrlm | RunLengthNonUniformity |  |
| lbp-3D-m1 | glrlm | RunLengthNonUniformityNormalized |  |
| lbp-3D-m1 | glrlm | RunPercentage |  |
| lbp-3D-m1 | glrlm | RunVariance |  |
| lbp-3D-m1 | glrlm | ShortRunEmphasis |  |
| lbp-3D-m1 | glrlm | ShortRunHighGrayLevelEmphasis |  |
| lbp-3D-m1 | glrlm | ShortRunLowGrayLevelEmphasis |  |
| lbp-3D-m1 | firstorder | 10Percentile |  |
| lbp-3D-m1 | firstorder | 90Percentile |  |
| lbp-3D-m1 | firstorder | Energy |  |
| lbp-3D-m1 | firstorder | InterquartileRange |  |
| lbp-3D-m1 | firstorder | Kurtosis |  |
| lbp-3D-m1 | firstorder | MeanAbsoluteDeviation |  |
| lbp-3D-m1 | firstorder | Mean |  |
| lbp-3D-m1 | firstorder | RobustMeanAbsoluteDeviation |  |
| lbp-3D-m1 | firstorder | RootMeanSquared |  |
| lbp-3D-m1 | firstorder | Skewness |  |
| lbp-3D-m1 | firstorder | TotalEnergy |  |
| lbp-3D-m1 | firstorder | Variance |  |
| lbp-3D-m1 | glszm | GrayLevelNonUniformity |  |
| lbp-3D-m1 | glszm | LargeAreaEmphasis |  |
| lbp-3D-m1 | glszm | LargeAreaHighGrayLevelEmphasis |  |
| lbp-3D-m1 | glszm | LargeAreaLowGrayLevelEmphasis |  |
| lbp-3D-m1 | glszm | SizeZoneNonUniformity |  |
| lbp-3D-m1 | glszm | SizeZoneNonUniformityNormalized |  |
| lbp-3D-m1 | glszm | SmallAreaEmphasis |  |
| lbp-3D-m1 | glszm | SmallAreaHighGrayLevelEmphasis |  |
| lbp-3D-m1 | glszm | SmallAreaLowGrayLevelEmphasis |  |
| lbp-3D-m1 | glszm | ZoneEntropy |  |
| lbp-3D-m1 | glszm | ZonePercentage |  |
| lbp-3D-m1 | glszm | ZoneVariance |  |
| lbp-3D-m1 | gldm | DependenceEntropy |  |
| lbp-3D-m1 | gldm | DependenceNonUniformity |  |
| lbp-3D-m1 | gldm | DependenceNonUniformityNormalized |  |
| lbp-3D-m1 | gldm | DependenceVariance |  |
| lbp-3D-m1 | gldm | GrayLevelNonUniformity |  |
| lbp-3D-m1 | gldm | LargeDependenceEmphasis |  |
| lbp-3D-m1 | gldm | LargeDependenceHighGrayLevelEmphasis |  |
| lbp-3D-m1 | gldm | LargeDependenceLowGrayLevelEmphasis |  |
| lbp-3D-m1 | gldm | SmallDependenceEmphasis |  |
| lbp-3D-m1 | gldm | SmallDependenceHighGrayLevelEmphasis |  |
| lbp-3D-m1 | gldm | SmallDependenceLowGrayLevelEmphasis |  |
| lbp-3D-m2 | glrlm | GrayLevelNonUniformity |  |
| lbp-3D-m2 | glrlm | LongRunEmphasis |  |
| lbp-3D-m2 | glrlm | LongRunHighGrayLevelEmphasis |  |
| lbp-3D-m2 | glrlm | LongRunLowGrayLevelEmphasis |  |
| lbp-3D-m2 | glrlm | RunEntropy |  |
| lbp-3D-m2 | glrlm | RunLengthNonUniformity |  |
| lbp-3D-m2 | glrlm | RunLengthNonUniformityNormalized |  |
| lbp-3D-m2 | glrlm | RunPercentage |  |
| lbp-3D-m2 | glrlm | RunVariance |  |
| lbp-3D-m2 | glrlm | ShortRunEmphasis |  |
| lbp-3D-m2 | glrlm | ShortRunHighGrayLevelEmphasis |  |
| lbp-3D-m2 | glrlm | ShortRunLowGrayLevelEmphasis |  |
| lbp-3D-m2 | firstorder | 10Percentile |  |
| lbp-3D-m2 | firstorder | 90Percentile |  |
| lbp-3D-m2 | firstorder | Energy |  |
| lbp-3D-m2 | firstorder | InterquartileRange |  |
| lbp-3D-m2 | firstorder | Kurtosis |  |
| lbp-3D-m2 | firstorder | Maximum |  |
| lbp-3D-m2 | firstorder | MeanAbsoluteDeviation |  |
| lbp-3D-m2 | firstorder | Mean |  |
| lbp-3D-m2 | firstorder | Median |  |
| lbp-3D-m2 | firstorder | Range |  |
| lbp-3D-m2 | firstorder | RobustMeanAbsoluteDeviation |  |
| lbp-3D-m2 | firstorder | RootMeanSquared |  |
| lbp-3D-m2 | firstorder | Skewness |  |
| lbp-3D-m2 | firstorder | TotalEnergy |  |
| lbp-3D-m2 | firstorder | Variance |  |
| lbp-3D-m2 | glszm | GrayLevelNonUniformity |  |
| lbp-3D-m2 | glszm | LargeAreaEmphasis |  |
| lbp-3D-m2 | glszm | LargeAreaHighGrayLevelEmphasis |  |
| lbp-3D-m2 | glszm | LargeAreaLowGrayLevelEmphasis |  |
| lbp-3D-m2 | glszm | SizeZoneNonUniformity |  |
| lbp-3D-m2 | glszm | SizeZoneNonUniformityNormalized |  |
| lbp-3D-m2 | glszm | SmallAreaEmphasis |  |
| lbp-3D-m2 | glszm | SmallAreaHighGrayLevelEmphasis |  |
| lbp-3D-m2 | glszm | SmallAreaLowGrayLevelEmphasis |  |
| lbp-3D-m2 | glszm | ZoneEntropy |  |
| lbp-3D-m2 | glszm | ZonePercentage |  |
| lbp-3D-m2 | glszm | ZoneVariance |  |
| lbp-3D-m2 | gldm | DependenceEntropy |  |
| lbp-3D-m2 | gldm | DependenceNonUniformity |  |
| lbp-3D-m2 | gldm | DependenceNonUniformityNormalized |  |
| lbp-3D-m2 | gldm | DependenceVariance |  |
| lbp-3D-m2 | gldm | GrayLevelNonUniformity |  |
| lbp-3D-m2 | gldm | LargeDependenceEmphasis |  |
| lbp-3D-m2 | gldm | LargeDependenceHighGrayLevelEmphasis |  |
| lbp-3D-m2 | gldm | LargeDependenceLowGrayLevelEmphasis |  |
| lbp-3D-m2 | gldm | SmallDependenceEmphasis |  |
| lbp-3D-m2 | gldm | SmallDependenceHighGrayLevelEmphasis |  |
| lbp-3D-m2 | gldm | SmallDependenceLowGrayLevelEmphasis |  |
| lbp-3D-k | glcm | Autocorrelation |  |
| lbp-3D-k | glcm | ClusterProminence |  |
| lbp-3D-k | glcm | ClusterShade |  |
| lbp-3D-k | glcm | ClusterTendency |  |
| lbp-3D-k | glcm | Contrast |  |
| lbp-3D-k | glcm | Correlation |  |
| lbp-3D-k | glcm | DifferenceAverage |  |
| lbp-3D-k | glcm | DifferenceEntropy |  |
| lbp-3D-k | glcm | DifferenceVariance |  |
| lbp-3D-k | glcm | Id |  |
| lbp-3D-k | glcm | Idm |  |
| lbp-3D-k | glcm | Idmn |  |
| lbp-3D-k | glcm | Idn |  |
| lbp-3D-k | glcm | Imc1 |  |
| lbp-3D-k | glcm | Imc2 |  |
| lbp-3D-k | glcm | InverseVariance |  |
| lbp-3D-k | glcm | JointAverage |  |
| lbp-3D-k | glcm | JointEnergy |  |
| lbp-3D-k | glcm | JointEntropy |  |
| lbp-3D-k | glcm | MCC |  |
| lbp-3D-k | glcm | MaximumProbability |  |
| lbp-3D-k | glcm | SumAverage |  |
| lbp-3D-k | glcm | SumEntropy |  |
| lbp-3D-k | glcm | SumSquares |  |
| lbp-3D-k | glrlm | GrayLevelNonUniformity |  |
| lbp-3D-k | glrlm | GrayLevelNonUniformityNormalized |  |
| lbp-3D-k | glrlm | GrayLevelVariance |  |
| lbp-3D-k | glrlm | HighGrayLevelRunEmphasis |  |
| lbp-3D-k | glrlm | LongRunEmphasis |  |
| lbp-3D-k | glrlm | LongRunHighGrayLevelEmphasis |  |
| lbp-3D-k | glrlm | LongRunLowGrayLevelEmphasis |  |
| lbp-3D-k | glrlm | LowGrayLevelRunEmphasis |  |
| lbp-3D-k | glrlm | RunEntropy |  |
| lbp-3D-k | glrlm | RunLengthNonUniformity |  |
| lbp-3D-k | glrlm | RunLengthNonUniformityNormalized |  |
| lbp-3D-k | glrlm | RunPercentage |  |
| lbp-3D-k | glrlm | RunVariance |  |
| lbp-3D-k | glrlm | ShortRunEmphasis |  |
| lbp-3D-k | glrlm | ShortRunHighGrayLevelEmphasis |  |
| lbp-3D-k | glrlm | ShortRunLowGrayLevelEmphasis |  |
| lbp-3D-k | firstorder | 10Percentile |  |
| lbp-3D-k | firstorder | 90Percentile |  |
| lbp-3D-k | firstorder | Energy |  |
| lbp-3D-k | firstorder | Entropy |  |
| lbp-3D-k | firstorder | InterquartileRange |  |
| lbp-3D-k | firstorder | Kurtosis |  |
| lbp-3D-k | firstorder | Maximum |  |
| lbp-3D-k | firstorder | MeanAbsoluteDeviation |  |
| lbp-3D-k | firstorder | Mean |  |
| lbp-3D-k | firstorder | Median |  |
| lbp-3D-k | firstorder | Minimum |  |
| lbp-3D-k | firstorder | Range |  |
| lbp-3D-k | firstorder | RobustMeanAbsoluteDeviation |  |
| lbp-3D-k | firstorder | RootMeanSquared |  |
| lbp-3D-k | firstorder | Skewness |  |
| lbp-3D-k | firstorder | TotalEnergy |  |
| lbp-3D-k | firstorder | Uniformity |  |
| lbp-3D-k | firstorder | Variance |  |
| lbp-3D-k | glszm | GrayLevelNonUniformity |  |
| lbp-3D-k | glszm | GrayLevelNonUniformityNormalized |  |
| lbp-3D-k | glszm | GrayLevelVariance |  |
| lbp-3D-k | glszm | HighGrayLevelZoneEmphasis |  |
| lbp-3D-k | glszm | LargeAreaEmphasis |  |
| lbp-3D-k | glszm | LargeAreaHighGrayLevelEmphasis |  |
| lbp-3D-k | glszm | LargeAreaLowGrayLevelEmphasis |  |
| lbp-3D-k | glszm | LowGrayLevelZoneEmphasis |  |
| lbp-3D-k | glszm | SizeZoneNonUniformity |  |
| lbp-3D-k | glszm | SizeZoneNonUniformityNormalized |  |
| lbp-3D-k | glszm | SmallAreaEmphasis |  |
| lbp-3D-k | glszm | SmallAreaHighGrayLevelEmphasis |  |
| lbp-3D-k | glszm | SmallAreaLowGrayLevelEmphasis |  |
| lbp-3D-k | glszm | ZoneEntropy |  |
| lbp-3D-k | glszm | ZonePercentage |  |
| lbp-3D-k | glszm | ZoneVariance |  |
| lbp-3D-k | gldm | DependenceEntropy |  |
| lbp-3D-k | gldm | DependenceNonUniformity |  |
| lbp-3D-k | gldm | DependenceNonUniformityNormalized |  |
| lbp-3D-k | gldm | DependenceVariance |  |
| lbp-3D-k | gldm | GrayLevelNonUniformity |  |
| lbp-3D-k | gldm | GrayLevelVariance |  |
| lbp-3D-k | gldm | HighGrayLevelEmphasis |  |
| lbp-3D-k | gldm | LargeDependenceEmphasis |  |
| lbp-3D-k | gldm | LargeDependenceHighGrayLevelEmphasis |  |
| lbp-3D-k | gldm | LargeDependenceLowGrayLevelEmphasis |  |
| lbp-3D-k | gldm | LowGrayLevelEmphasis |  |
| lbp-3D-k | gldm | SmallDependenceEmphasis |  |
| lbp-3D-k | gldm | SmallDependenceHighGrayLevelEmphasis |  |
| lbp-3D-k | gldm | SmallDependenceLowGrayLevelEmphasis |  |
| lbp-3D-k | ngtdm | Busyness |  |
| lbp-3D-k | ngtdm | Coarseness |  |
| lbp-3D-k | ngtdm | Complexity |  |
| lbp-3D-k | ngtdm | Contrast |  |
| lbp-3D-k | ngtdm | Strength |  |
